# Cell encapsulated biomaterials for translational medicine

**DOI:** 10.1016/j.bioactmat.2025.10.021

**Published:** 2025-10-27

**Authors:** Mayakrishnan Arumugam, Yunyang Zhang, Ying Huang, Ramesh Kannan Perumal, Ting Zhang, Xiangdong Kong, Ruibo Zhao

**Affiliations:** a Institute of Smart Biomedical Materials, School of Materials Science & Engineering, Zhejiang Sci-Tech University, Hangzhou, 310018, China; b Zhejiang-Mauritius Joint Research Center for Biomaterials and Tissue Engineering, Zhejiang Sci-Tech University, Hangzhou, 310018, China; c State Key Laboratory of Bio-based Fiber Materials, Zhejiang Sci-Tech University, Hangzhou, 310018, China; d Shanghai Ninth People's Hospital, Shanghai Jiao Tong University School of Medicine, Shanghai, 200000, China

**Keywords:** Biomaterials, Cell encapsulation, Immune cell, Tissue regeneration, Cancer therapy

## Abstract

Biomaterial supported cell encapsulation matrices have demonstrated superior properties for enhancing biological functionality, making them highly significant for translational medicine across multiple therapeutic applications. This review examined how biomaterials interact with cellular therapies, including stem cells, immune cells, and fibroblasts across single-cell, multicellular, and core-shell structures. The biomaterial capsule plays a key role in improving cell viability, immune protection, and supporting tissue-specific interactions. Furthermore, this review highlights current trends in microfluidics, 3D printing, in situ preparation, and electrospraying self-assembly, each method offering different advantages for cell encapsulation matrices. Microfluidics allows precise control of capsule size and uniformity, making it suitable for single-cell and core-shell encapsulation. The 3D printing technologies empower accurate cell placement to build multicellular structures that mimic native tissue organization. In situ preparation directly encapsulates cells within the target tissue. Collectively, these techniques significantly influence the physical, chemical, and biological properties of encapsulated cells. Additionally, we discuss various biomaterials including natural proteins, polysaccharides, and synthetic polymers, each material offers unique benefits in terms of biocompatibility and biodegradability. The integration of living cells with biomaterial matrix cell encapsulation systems greatly exhibits mechanical strength, high porosity, and controlled drug release. Importantly, this review emphasises the dual role of the biomaterial capsule in cancer therapy, which enhances anti-tumor immune responses and promotes tissue regeneration, with a focus on bone, skin, neural tissue, liver, vascular structures, and skeletal muscle repair. In conclusion, cell-encapsulated biomaterials are a versatile platform supporting both cancer immunotherapy and regenerative medicine, underscoring their wide range of biomedical applications.

## Highlights

• Cell capsules are an alternative living biomaterial demonstrating significant potential for translational medicine.
• Cell encapsulation techniques play a crucial role in various biomedical applications.
• Biomaterials interact with cellular therapies, including stem cells, immune cells, and fibroblast cells.
• Cell encapsulated biomaterials are more supportive of cancer immunotherapy and tissue regeneration.

## Graphical abstract

**Figure:**
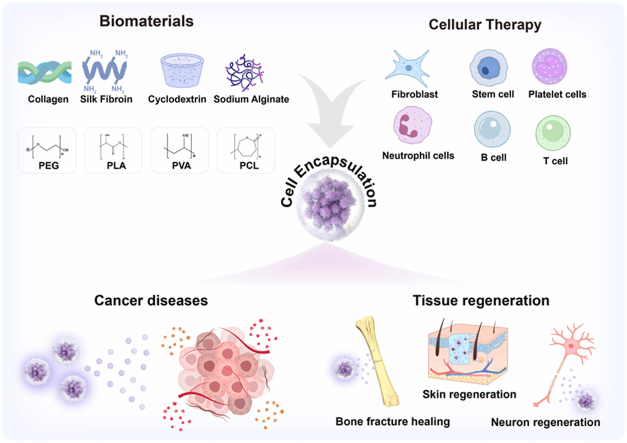

## 1 Introduction

Cell encapsulation is a powerful approach in translational medicine, offering innovative solutions for delivering therapeutic agents [1,2]. This technique creates a new environment that preserves cell viability, shields cells from immunological rejection, and enables the sustained release of therapeutic molecules, semi-permeable materials, and live cells, thereby protecting their functional properties for various biomedical applications [3,4]. The cell encapsulation matrix involves reactive oxygen species (ROS), autophagy, and the mTOR pathway in crucial processes that regulate cellular function [5,6]. Additionally, protecting the encapsulated biomaterial capsules can provide a controlled release of therapeutic agents, such as insulin or growth factors, which are essential for treating chronic conditions like diabetes and cancer [19,20]. For instance, encapsulated donor cells have been utilized in tissue engineering, such as bone marrow mononuclear cells, which promote tissue regeneration by recruiting host cells and forming vascular grafts [22]. This dynamic release of therapeutic molecules offers a significant advantage over traditional drug delivery systems, which may not provide side effects. Moreover, cell encapsulation techniques are highly sensitive to the physical and chemical properties, including cell proliferation and differentiation [23]. Ultimately, the cellular behaviour of encapsulation materials in line with therapeutic agents is of great significance in personalised medicine. Additionally, these cell encapsulation strategies play a crucial role in regulating the cellular activity, enhancing the therapeutic efficacy, and are supported by various biomedical applications [24,25].

**Fig. 1:**
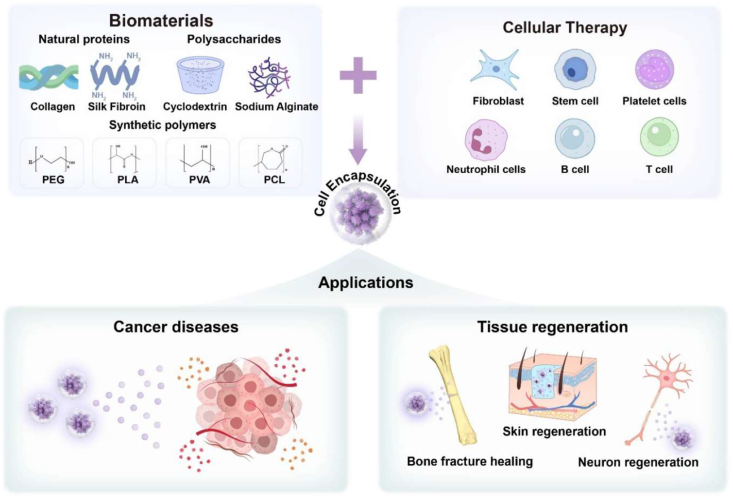
Schematic illustration of a biomaterial-based cell encapsulation platform designed for dual applications in cancer therapy and tissue regeneration. The diagram presents a multifunctional therapeutic system in bioactive cells (fibroblast cell, stem cell, platelet cells, neutrophil cell, B cell, and T cell) encapsulated with polymer matrix (collagen, silk fibroin, cyclodextrin, sodium alginate, polyethylene glycol (PEG), polylactic acid (PLA), polyvinyl alcohol (PVA), and polycaprolactone (PCL)). This encapsulated system serves two primary purposes. The left-side platform facilitates cancer therapy by delivering therapeutic agents that inhibit tumor growth and kill the cancer cells. The right side platform supports tissue regeneration of various tissues, including bone grafts, skin tissue, and neuronal network regeneration. (Scheme of the diagram created by bioself using Cinema 4D and Adobe Illustrator software).

Biomaterial-based cell encapsulation is more suitable for different biomedical fields, including cancer therapy [7], wound healing [8], tissue regeneration [9], and drug delivery [10]. The biomaterials are natural proteins (silk fibroin, collagen, gelatin, keratin, and elastin), polysaccharides (chitosan, sodium alginate, sodium hyaluronate, cellulose, and cyclodextrin), and synthetic polymers (polyethylene glycol (PEG), polylactic acid (PLA), polyvinyl alcohol (PVA), and polycaprolactone (PCL)), which offer significant advantages due to their superior properties for a wide range of biomedical applications [26,27]. These biomaterials are typically non-toxic to cells and maintain their biocompatibility during the degradation process, making them ideal materials for use as encapsulation matrices [30]. Moreover, encapsulation biomaterials require careful consideration of critical factors, including chemical composition, surface morphology, mechanical and chemical stability, and porosity [31,32]. Among these, porosity is particularly significant in facilitating nutrient diffusion and releasing biological activity from encapsulated cells. This type of material has a high porosity structure that facilitates access to nutrients and enables the release of metabolic products, while also supporting the cellular functional activity [14,16]. A key advantage of these biomaterial combinations is their high thermal and mechanical stability, excellent drug encapsulation capabilities, strong biocompatibility, and their essential role in maintaining encapsulated cell integrity, which is highly enhanced in the biological environments [35,36]. The schematic diagram of biomaterial combined cell encapsulation for treating the dual application of cancer diseases and tissue regeneration, including bone grafts, skin tissue, and neuronal network regeneration (Fig. 1). This diagram represents a multifunctional therapeutic system in bioactive cells (fibroblast cell, stem cell, platelet cells, neutrophil cell, B cell, and T cell) encapsulated with polymer matrix collagen, silk fibroin, cyclodextrin, sodium alginate, polyethylene glycol (PEG), polylactic acid (PLA), polyvinyl alcohol (PVA), and polycaprolactone (PCL). This encapsulated system has two primary applications such as cancer therapy by delivering therapeutic agents that inhibit tumor growth and kill cancer cells, and it promotes tissue regeneration in various tissues, including bone grafts, skin, and neuronal networks.

The encapsulation of immune cells, including T cells, natural killer (NK) cells, neutrophils, and macrophages, has become a significant approach in cancer therapy for destroying the tumor cells. For example, encapsulating T cells, particularly chimeric antigen receptor T (CAR-T) cells, has significantly enhanced their therapeutic effectiveness [37]. This encapsulation of CAR-T cells selectively targets to destroy cancer cells while offering additional benefits, such as prolonged persistence, improved functionality, and protection from the immunosuppressive tumor microenvironment. This approach has the potential to control the release of therapeutic agents, thereby directly enhancing their anti-tumor activity within the tumor microenvironment [[38], [39], [40]]. Similarly, NK cells naturally target tumors without prior sensitization and benefit from the encapsulation matrix, which improves their survival, ensures the sustained release of cytotoxic agents, and offers a promising treatment for cancers resistant to conventional therapies [41]. Neutrophils naturally infiltrate inflamed or malignant tissues; they can be encapsulated to deliver anticancer molecules directly to tumor sites. Additionally, macrophages with an anti-tumor phenotype can be encapsulated to sustain the release of pro-inflammatory cytokines or directly phagocytose tumor cells, thereby amplifying their therapeutic impact [42]. The encapsulation of mesenchymal stem cells (MSCs), induced pluripotent stem cells (iPSCs), pancreatic islet cells, hepatocytes, and endothelial cells has the potential to deliver regenerative therapeutic factors that modulate immune responses [43,44]. Still, the encapsulation matrix preserves their cell viability and functionality, while protecting them from immune rejection, making it an essential tool for tissue repair and regeneration. For instance, encapsulated MSCs and iPSCs have been shown to significantly improve damaged tissues, including cartilage, bone, and cardiac tissue [45,46]. Similarly, encapsulating functional cells, such as pancreatic islet cells or hepatocytes, facilitates the sustained release of therapeutic factors. The encapsulated pancreatic islet cells can continuously secrete insulin, providing a viable treatment for diabetes, while encapsulated hepatocytes support liver regeneration. This approach has long-term survival and functionality, minimizes immune rejection, and enhances its therapeutic efficacy [47].

The objective of this review discussed how biomaterials are utilized in cell encapsulation for translational medicine. We specifically discuss various types of biomaterials applied in cellular therapies involving immune cells, fibroblasts, stem cells, red blood cells, platelets, and neutrophils. Natural polymers, polysaccharides, and synthetic polymers are highlighted for their biocompatibility, mechanical strength, and capacity to form protective barriers that preserve cell functionality. In addition, this review evaluates different encapsulation techniques, including microfluidics, 3D printing, in situ preparation, and the electrospraying self-assembly method, which have proven effective in maintaining cell viability, optimizing cellular functionality, and adapting to biological environments. Importantly, these approaches transform multiple medical fields, particularly cancer therapy and tissue regeneration. Their effectiveness is examined through *in vitro*, *ex vivo*, and *in vivo* models, with special emphasis on their relevance to personalised therapies for bone, skin, liver, neural repair, skeletal muscle, and vascular tissue regeneration. Overall, this review highlights recent trends in biomaterials-based cell encapsulation technologies for various biomedical applications.

## 2 Cell capsules

**Fig. 2:**
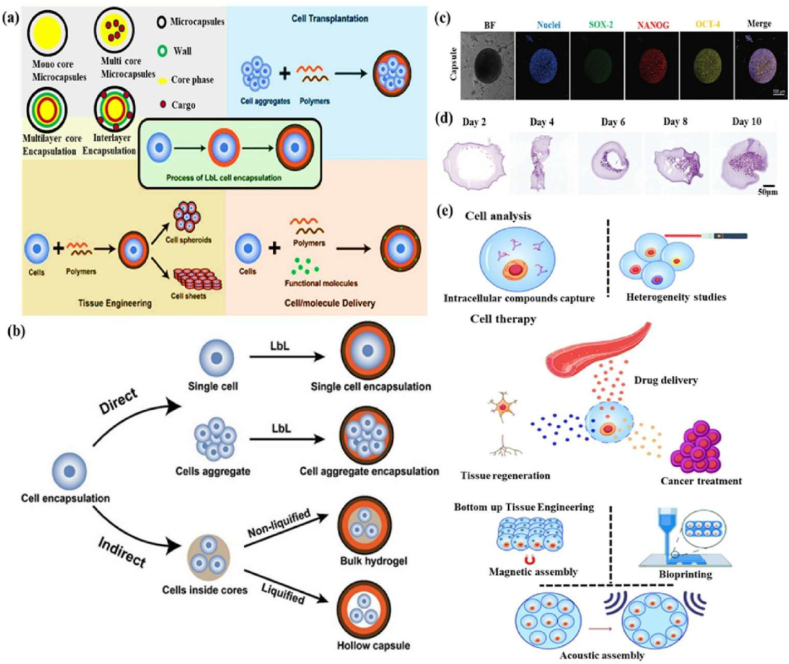
(a&b) Depicts the cells interacting with a polymer during the cell encapsulation process using a layer-by-layer (LbL) self-assembly method. Reproduced with permission from Ref. [240], Copyright 2018, Wiley-VCH. (c) Immunofluorescence microscopy images of iPSC capsules, labelled with Hoechst (blue), SOX-2 antibody (green), NANOG antibody (red), and OCT-4 antibody (yellow). Scale bar 100 μm. Reproduced with permission from Ref. [241], Copyright 2024, Wiley-VCH. (d) Histological analysis of iPSC capsules was performed at days 2, 4, 6, 8, and 10 post-encapsulation, and structural changes were evaluated by hematoxylin and eosin (H&E) staining. Scale bar 50 μm. Reproduced with permission from Ref. [241], Copyright 2024, Wiley-VCH. (e) Shows the single-cell strategies of cell analysis in intracellular compound capture and heterogeneity study of different biomedical applications, Reproduced from Ref. [242], Copyright 2024, Wiley-VCH.

Cell capsules are micro-to-nanoscale structures designed to encapsulate living cells or bioactive compounds [50]. The potential applications of cell capsules span a wide range of fields, including medicine, biotechnology, materials science, and environmental science [51,52]. In biomedical science, cell capsules are especially significant due to their multifunctional design, which is specifically tailored to protect and sustain the viability of encapsulated cells [53]. Typically made from biocompatible materials such as polysaccharides, proteins, or synthetic polymers, these capsules feature a protective outer shell cellular structure [54]. Their applications span various areas, including drug delivery, tissue engineering, and microbial immobilization, offering advantages such as targeted delivery, enhanced stability, and controlled release. In drug delivery, cell capsules enable the controlled and sustained release of therapeutic agents at targeted sites, improving the efficiency and precision of treatments [55]. In tissue engineering, they serve as scaffolds for cell growth and differentiation, supporting tissue repair and regeneration. Furthermore, cell capsules have been beneficial for microbial immobilization, enhancing the stability and effectiveness of these microbes in processes such as fermentation, bioremediation, and probiotic formulations [56]. Schematic diagram of cells interacting with the polymer in the cell encapsulation process using the layer-by-layer (LbL) self-assembly method (Fig. 2 a&b). The immunofluorescence microscopy images of iPSC capsules, which were fluorescently labelled with different colors of Hoechst (blue), SOX-2 antibody (green), NANOG antibody (red), and OCT-4 antibody (yellow), are shown in Fig. 2c. Histological analysis of iPSC capsules was performed at days 2, 4, 6, 8, and 10 post-encapsulation, and structural changes were evaluated by hematoxylin and eosin (H&E) staining (Fig. 2d).

### 2.1 Types of cell capsules

#### 2.1.1 Single-cell capsule

The efficiency of single-cell encapsulation in conventional droplet microfluidic devices is largely determined by the cell density in the aqueous phase medium, and ensuring effective chip fabrication approaches. In recent years, single-cell encapsulation has gained significant attention due to its potential in precise drug delivery, bioprinting, and tissue engineering [57]. Compared with multicellular encapsulation strategies, single-cell capsules offer improved circulation in the bloodstream and reduce the risk of entrapment. On the other hand, droplet microfluidics has emerged as a transformative platform for single-cell encapsulation, providing precise control over the microenvironment of individual cells. By producing uniform droplets in the microliter-to-nanoliter range, this technology enables high-throughput isolation of single cells within biocompatible matrices. The resulting microcapsules establish well-defined functions that preserve cell viability while permitting fine-tuned modulation of cellular behaviour, making them especially valuable in precision medicine. In regenerative medicine and immunotherapy, droplet microfluidics enables encapsulation of stem cells, engineered immune cells, or pancreatic islets within tailored microenvironments that enhance their survival and functional activity [34]. Such microcapsules can be further engineered to release paracrine factors, immunomodulatory cues, or extracellular matrix to amplify the therapeutic potential of individual cells. Beyond regenerative applications, droplet microfluidics offers powerful tools for investigating immune cell dynamics at the single-cell level. Encapsulation enables the real-time monitoring of cytokine secretion, antigen recognition, and clonal expansion, thereby revealing functional heterogeneity within immune populations. Also, droplet-based methods enable the generation of diverse single-cell units, including those designed for T-cell therapies [58]. The single-cell strategies of cell analysis in intracellular compound capture and heterogeneity study of different biomedical applications (Fig. 2e). The functionality of single-cell capsules depends strongly on their material composition, which often includes polysaccharides, proteins, or polypeptides. Polysaccharide capsules are classified into two main types: exopolysaccharides (EPS) and capsular polysaccharides (CPS) [59,60]. The polypeptide systems have recently gained significant attention in biomedical applications. They offer distinct advantages as smart drug delivery vehicles, with the ability to respond to physiological signals such as pH, temperature, or enzymatic activity [61]. This adaptability enables more personalised treatments, minimizes long-term accumulation, and facilitates targeted drug delivery, particularly by releasing therapeutic agents directly at tumor sites.

#### 2.1.2 Multicell capsules

**Fig. 3:**
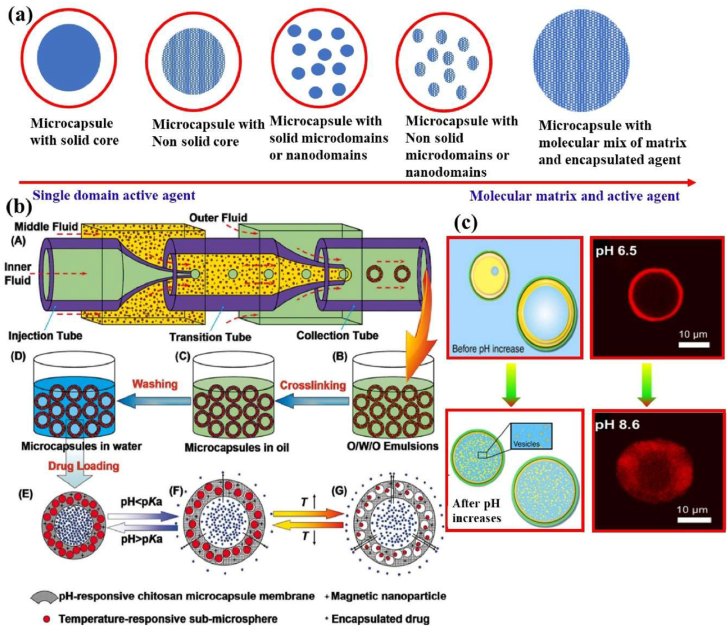
(a) Description of the multicore microcapsule system of a single domain and molecule mixed active agent of cell encapsulated structure. Reproduced with permission from Ref. [243], Copyright 2023 Elsevier. (b) Schematic diagram of microcapsule (A–D) multi-stimuli-responsive microcapsules with customizable controlled-release, (*E*–G) schematic diagram of pH and temperature-based fabrication method of controlled-drug release mechanism. Reproduced with permission from Ref. [243], Copyright 2023 Elsevier. (c) Microcapsules analysis of different pH levels (6.5–8.6) medium with changes in morphological structure. Scale bar 10 μm. Reproduced with permission from Ref. [243], Copyright 2023 Elsevier.

A multicell capsule is a transformative system of advanced technology in the field of drug delivery, offering a sophisticated platform for precise medicine. The multicell capsule design enables improved control over drug release kinetics, enhanced payload protection, and increased biocompatibility factors that are crucial for achieving optimal therapeutic outcomes [64,65]. These capsules can be engineered with diverse properties, including variations in wall thickness, mechanical strength, permeability, and responsiveness to external stimuli. The multicore microcapsule system of a single domain and molecule mixed active agent of cell encapsulated structure (Fig. 3a). This modularity enables the simultaneous or sequential release of several therapeutic agents. The fabrication of multi-stimuli-responsive microcapsules, which can respond to changes in environmental conditions such as temperature, pH, or enzymatic activity (Fig. 3b). These capsules are capable of encapsulating a wide range of therapeutic agents, including small-molecule drugs, bioactive enzymes, and even complex structures like liquid crystal droplets. This responsiveness makes them particularly suitable for targeting disease microenvironments, such as the acidic milieu of tumor tissues or the inflamed sites of infection. Furthermore, multilayer capsules offer the advantage of integrating diverse functional components organic dyes for imaging, inorganic nanoparticles for photothermal effects, carbon nanotubes for mechanical reinforcement, or antibodies for active targeting. Additionally, the integration of therapeutic and diagnostic functions enables real-time monitoring of treatment efficacy. The morphological changes of microcapsules under varying pH levels (6.5–8.6) highlight their potential in pH-sensitive drug release (Fig. 3c). This adaptability is particularly useful in targeting diseases with localized pH variations, such as cancerous tissues, where the extracellular pH tends to be more acidic than normal tissues. The ability to encapsulate a diverse array of molecules, including peptides, proteins, and genetic materials, further underscores the utility of multicell capsules in personalised medicine. Their design minimizes long-term accumulation in the body, which is a common concern with traditional drug delivery systems. This approach reduces the risk of chronic toxicity and supports the development of safer, more efficient long-term therapies. In applications such as cancer therapy, these multicell capsules enable the staged delivery of chemotherapeutic agents, improving therapeutic index and reducing systemic side effects [66]. This capability is vital for achieving sustained drug action while minimizing peak-trough fluctuations that can compromise efficacy or safety. The multicell capsule system represents a robust and adaptable platform for next-generation drug delivery. Its ability to provide precise control over release profiles, target-specific delivery, and multifunctional payload integration makes it a powerful tool in the advancement of biomedical therapeutics. As research continues to evolve, multicell capsules are poised to play a pivotal role in personalised medicine of targeted therapies.

#### 2.1.3 Core-shell structure

The core-shell microcapsules can be classified into single-core shells (inner core) and multi-core shells (outer core). The inner core typically contains the primary active component, while the outer shell acts as a protective functional barrier [67]. The core-shell structure exhibits unique properties by combining different materials, and playing multiple roles, such as shielding the encapsulated material from external stressors, enabling the sustained release of bioactive compounds, and allowing selective permeability for essential nutrients [68]. These features ensure controlled drug release, protect cells from environmental stress, and help maintain optimal cell health and functionality. The author Huihua Huang et al. [69] describe gellan gum-based microcapsules with core-shell structures. In these systems, it generally serves as the active component, while providing mechanical stability and ensuring controlled release. Fabricating core-shell structures involves diverse techniques, such as high-temperature evaporation, dispersion polymerization, laser ablation, carbothermal reduction, and hydrothermal methods [70]. These methods enable the creation of porous and bioactive core-shell capsules that can immobilize various microorganisms. Compared to single-component particles, core-shell structures exhibit superior chemical and physical properties, making them particularly effective in designing nano-delivery systems for the controlled release of therapeutic agents, including drugs and vitamins [71]. These microcapsules, with larger core volumes, also display higher drug-loading capacities and delivery efficiency [72]. The applications of core-shell structures extend across drug delivery, cosmetics, tissue engineering, and microbial immobilization, making them multipurpose in various industries.

### 2.2 Preparation method of cell capsules

#### 2.2.1 Microfluidics

**Fig. 4:**
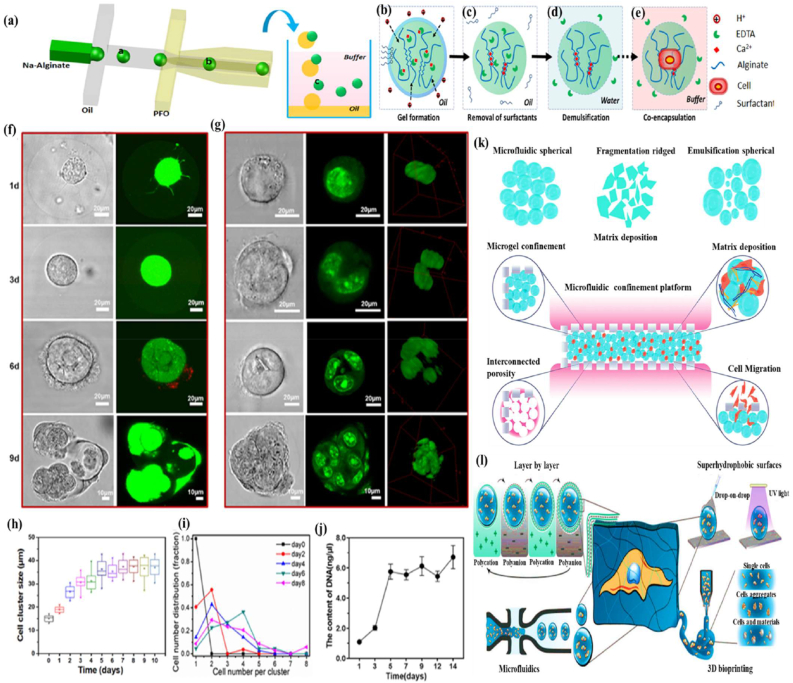
(a) The design of the microfluidic device in the schematic illustration of the PDMS-based microfluidic device used for fabricating alginate microgels. Reproduced with permission from Ref. [245]. Copyright 2020, Elsevier. (b) Formation of cell-encapsulating microgel in a laminar flow of alginate solution is disrupted into droplets via a flow-focusing junction. Acetic acid present in the oil phase diffuses into these aqueous droplets, triggering the release of Ca^2+^ ions from Ca-EDTA complexes, which in turn initiates alginate gelation. Reproduced with permission from Ref. [245], Copyright 2020, Elsevier. (c) The addition of PFO to the oil phase removes surfactants, destabilising the droplet interface. Reproduced with permission from Ref. [245], Copyright 2020, Elsevier. (d) Alginate microgels are then transferred into an aqueous phase. Reproduced with permission from Ref. [245], Copyright 2020, Elsevier. (e) Rapid gelation driven by Ca^2+^ cross-linking enables the encapsulation of cells within the microgels. By promptly collecting the encapsulated cells, high cell viability is maintained by minimizing prolonged exposure to acidic conditions. Reproduced with permission from Ref. [245]. Copyright 2020, Elsevier. (f) Confocal microscopy images at high magnification show MSCs encapsulated in RGD-modified alginate microgels and cultured in proliferation medium. Live and dead cells were visualised using calcein and ethidium homodimer staining, respectively. The encapsulated MSCs proliferated over time and eventually migrated out of the microgels. Despite proliferation, cells retained a spherical shape due to the stiffness of the alginate matrix. Reproduced with permission from Ref. [245], Copyright 2020, Elsevier. (g) Confocal images of MSCs stained with Syto 9 nuclei dye show continued proliferation within the microgels, indicated by increasing cell numbers. Reproduced with permission from Ref. [245], Copyright 2020, Elsevier. (h) Quantification of the average diameter of cell clusters within the microgels over the culture period. Reproduced with permission from Ref. [245]. Copyright 2020, Elsevier. (i&j) Cell distribution and DNA analysis of MSCs encapsulated within the microgels were denoted at different time points. Reproduced with permission from Ref. [245]. Copyright 2020, Elsevier. (k) Microfluidic confinement platform for cell invasion into various types of granular materials, such as microfluidic spherical, fragmentation-ridged, and emulsification spherical structures. Reproduced with permission from Ref. [244]. Copyright 2024, Wiley-VCH. (l) The layer-by-layer microfluidics, superhydrophobic surfaces, and 3D bioprinting utilise cell encapsulation systems. Reproduced with permission from Ref. [246]. Copyright 2020, Wiley-VCH.

A microfluidic cell capsule is a tiny, precisely designed structure created using microfluidic technology to encapsulate individual cells or the core of the cells. It can be categorised into hydrodynamics, interfacial, and electrohydrodynamics, each tailored to specific applications, offering unique advantages. Hydrodynamics-based microfluidics is widely used for producing materials due to its high throughput and precision. This method involves a continuous phase flowing alongside a dispersed, immiscible phase, allowing precise control over the behaviour of the dispersed fluid [75]. The interfacial-based microfluidics is a simpler, cost-effective approach, typically using an adjustable vibration motor, a continuous-phase vessel, and a capillary nozzle to produce different types such as microemulsions, microparticles, and microfibers. Electrohydrodynamic microfluidics, an established platform, is particularly effective for producing submicron to nanoscale capsules. Generally, this method used to prepare the capsules that obtain a high surface area-to-volume ratio, which enhances properties such as adsorption, catalysis, and controlled drug release [76]. A relatively simple setup allows the production of nanocomposites that serve as fillers or coatings in composite materials, which have been developed for multi-drug encapsulation in advanced drug delivery systems. These capsules are typically made from biocompatible materials that replicate the natural microenvironment. By utilising advanced microfluidic techniques, researchers can create highly precise micro-to-nanoscale structures. Design of the microfluidic device in the schematic illustration of PDMS-based microfluidic device used for fabricating alginate microgels (Fig. 4a). Formation of cell-encapsulating microgel in a laminar flow of alginate solution is disrupted into droplets via a flow-focusing junction. Acetic acid present in the oil phase diffuses into these aqueous droplets, triggering the release of Ca^2+^ ions from Ca-EDTA complexes, which in turn initiates alginate gelation (Fig. 4b). The addition of PFO to the oil phase removes surfactants, destabilising the droplet interface (Fig. 4c). Alginate microgels are then transferred into an aqueous phase (Fig. 4d). Rapid gelation driven by Ca^2+^ cross-linking enables the encapsulation of cells within the microgels (Fig. 4e). Confocal microscopy images of MSCs encapsulated in RGD-modified alginate microgels and cultured in proliferation medium. Live and dead cells were visualised using live/dead staining (Fig. 4f). The encapsulated MSCs proliferated over time and eventually migrated out of the microgels. Despite proliferation, cells retained a spherical shape due to the stiffness of the alginate matrix. Confocal images of MSCs stained with Syto 9 nuclei dye show continued proliferation within the microgels, indicated by increasing cell numbers (Fig. 4g). Quantification of the average diameter of cell clusters within the microgels over the culture period (Fig. 4h). The cell distribution and DNA analysis of MSCs encapsulated within the microgels were denoted at different time points (Fig. 4(i and j). Illustrates the microfluidic confinement platform for cell invasion into various types of granular materials, such as microfluidic spherical, fragmentation-ridged, and emulsification spherical structures (Fig. 4k). The layer-by-layer microfluidics, superhydrophobic surfaces, and 3D bioprinting utilise cell encapsulation systems represented (Fig. 4 l). Moreover, microfluidics-based encapsulation offers exceptional control over capsule size, shape, and composition, making it highly effective for various applications, including cancer cell therapy [73], targeted drug delivery [74], and tissue engineering [77]. The microfluid encapsulation functions as an independent immunoprotective solution or as part of a combined strategy alongside other immunomodulatory approaches. However, microcapsules and macroencapsulation devices without immunosuppressants have yet to achieve complete insulin independence following islet implantation. For example, while semipermeable coatings and membranes block the entry of immune cells, they also restrict the transport of nutrients and oxygen to the islets. Additionally, these barriers may fail to fully contain small antigens secreted by encapsulated cells, potentially triggering indirect immune responses [287]. Another challenge is the interaction between the host tissue and the implanted biomaterial, which can lead to fibrotic overgrowth on the encapsulating surface. This fibrosis obstructs vascular access, further compromising islet survival. Moreover, micro and nanocapsule strategies are typically designed for individual islets or small clusters, whereas macroencapsulation devices can accommodate hundreds or thousands of islets. These approaches rely on semipermeable biomaterials such as hydrogels in microcapsules or porous membranes in macroencapsulation to physically isolate islets while allowing for selective molecular exchange. Otherwise, macroencapsulation devices are often implanted in the subcutaneous tissue due to their ease of access for both implantation and retrieval. Additionally, these biomaterials should prevent immune cell infiltration and block harmful cytokines, while allowing the diffusion of essential biomolecules, such as glucose, insulin, and nutrients, to sustain islet function [288,289]. Furthermore, hydrogel capsules may create additional barriers to oxygen diffusion, thereby supporting long-term islet survival and regulating fibrotic responses.

#### 2.2.2 3D printing capsule

**Fig. 5:**
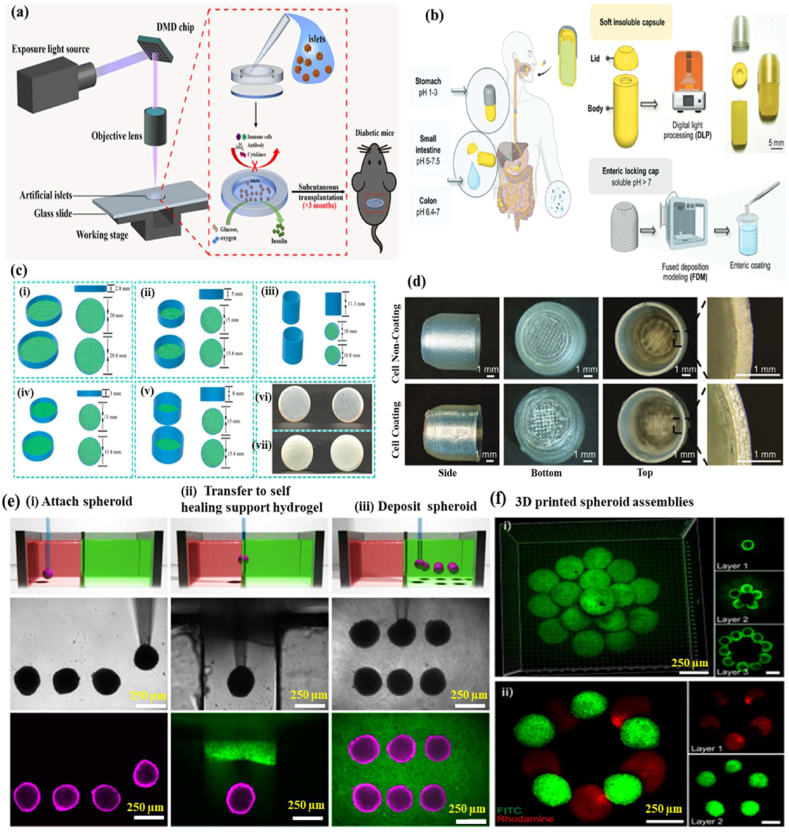
(a) 3D printed capsule device designed for islet delivery to treat diabetic mice without the use of immunosuppressants. Reproduced with permission from Ref. [247], Copyright 2022, ACS. (b) Schematic diagram of gastrointestinal targeting capsules filled with an aqueous solution. These capsules protect their contents during gastrointestinal transit in the stomach, early intestine (pH < 7), and late intestine or colon (pH > 7). The insoluble body and lid are made using DLP 3D printing, while the soluble enteric locking cap is produced via FDM 3D printing with a water-soluble filament. Reproduced from Ref. [248], Copyright 2024, Wiley-VCH. (c) Schematic representation (i–v) Different capsule structures, (vi-vii) Capsules photographs images. Reproduced from Ref. [249], Copyright 2022, Frontiers. (d) Capsule surface images with and without cell coating, including side, bottom, and top views of the FDM-based 3D printed capsule structure. Reproduced from Ref. [248], Copyright 2024, Wiley-VCH. (e) 3D printing of cell capsule in self-healing hydrogel elongation (top), brightfield images (middle), and fluorescent images (bottom) (i) Attachment of MSC spheroids in a media reservoir, (ii) Transfer of spheroids into a self-healing hydrogel, (iii) Deposition of spheroids within the hydrogel by releasing vacuum from the micropipette tip. Scale bars 250 μm. Reproduced from Ref. [285], Copyright 2021, Nature communications. (f) 3D printed spheroids assemble (i) Multi-layer cone-shaped geometry (FITC-labelled spheroids), (ii) Layered rings of distinct MSC spheroid populations (FITC and rhodamine-labelled). Scale bars 250 μm. Reproduced from Ref. [285], Copyright 2021, Nature communications.

3D printing technology, also known as additive manufacturing, is a process that creates three-dimensional structures from computer-aided design models. The 3D printing capsules are made from complex cell materials and represent a cutting-edge advancement in biomedical technology. The capsules combine living cells with biocompatible materials to form structures that support cell survival and functionality. Additionally, it can be precisely engineered to include core-shell structures, which help protect sensitive cells or bioactive substances. Furthermore, 3D printing capsules are more advanced in the biomedical field because these properties are highly suitable for various applications, including drug delivery, tissue engineering, and regenerative medicine [78]. The 3D-printed capsule is designed for islet delivery to treat diabetic mice without the use of immunosuppressants (Fig. 5a). The combination of customizable design, cell compatibility, and controlled release mechanisms highlights its transformative potential in the field of healthcare innovation. This innovative approach has shown immense potential in biomedical research, developing into an interdisciplinary field that integrates bioengineering and pharmaceutical sciences [79]. In recent years, 3D printing has made significant strides in capsule production, enabling the creation of customised functional capsules with intricate internal architectures. Schematic diagram of gastrointestinal targeting capsules filled with an aqueous solution. These capsules protect their contents during gastrointestinal transit in the stomach, early intestine (pH < 7), and late intestine or colon (pH > 7). The insoluble body and lid are made using DLP 3D printing, while the soluble enteric locking cap is produced via FDM 3D printing with a water-soluble filament (Fig. 5b). Moreover, capsules produced through 3D printing can now be precisely controlled in size, shape, and porosity, offering tailored properties that enhance the release, containment, and flow of reagents or catalysts. This technology not only boosts production efficiency but also reduces manufacturing costs. Nowadays, 3D-printed drug delivery systems have revolutionized the sustained and controlled release of therapeutic compounds in the pharmaceutical industry. The schematic representation of different capsule structures (i-v) and photographed images of the capsules (vi-vii) as shown in Fig. 5c. These capsules, often designed as solid oral dosage forms, encapsulate life-saving drugs, vitamins, minerals, and other therapeutic agents, ensuring precise dosing, portability, and improved patient compliance [80,81]. Additionally, 3D printing provides innovative solutions for challenging formulations, including liquids, powders, and pastes. The manufacturing process typically begins with preparing polymer filaments customized to meet specific design requirements [82]. The capsule surface images with and without cell coating, including side, bottom, and top views of the FDM-based 3D printed capsule structure, are shown in Fig. 5d. Moreover, the personalised medicine enabled by 3D printing technology continues to evolve, offering significant potential to advance healthcare and drive economic growth. For instance, Maroni et al. [83] developed a capsule with two distinct compartments made of polyvinyl alcohol, providing an innovative and convenient solution for drug delivery systems.

Otherwise, a cell spheroid is a three-dimensional aggregation of cells that self-assemble *in vitro*, closely mimicking the natural cellular microenvironment found in tissues. In tissue engineering and regenerative medicine, human pluripotent stem cells (hPSCs) make a significant contribution to cell differentiation into functioning adult tissues and must continue to exhibit robust and scalable functionality. These forms exist because of the innate ability of cells to adhere to or interact with the extracellular matrix. Unlike traditional 2D cell cultures, spheroids more accurately replicate the biochemical and mechanical cues of *in vivo* conditions, making them valuable models for studying cell behaviour, disease progression, and drug response [290]. The 3D printing of spheroids in self-healing support hydrogels. (i) Attachment of MSC spheroids in a media reservoir, (ii) Transfer of spheroids into a self-healing support hydrogel, (iii) Deposition of spheroids within the hydrogel by releasing vacuum from the micropipette tip shown in Fig. 5e. In 3D printed spheroids assemble (i) multi-layer cone-shaped geometry (FITC-labelled spheroids), (ii) layered rings of distinct MSC spheroid populations (FITC and rhodamine-labelled) as shown in Fig. 5f. Moreover, the spheroids exhibit a remarkable capacity for self-organisation, including the formation of specialized organoid structures derived from stem cells. These spheroids serve as advanced models for replicating the physiological and functional properties of organs such as the intestine, liver, kidney, brain, and heart, making them invaluable for studying human development and disease. Their structural and functional superiority over traditional monolayer cultures also makes them promising platforms for drug screening [291]. The organotypic cell densities in spheroids enhance ECM interactions, which are essential for maintaining cellular differentiation and phenotype interactions that are significantly limited in conventional 2D cultures. Moreover, high cell densities within spheroids are crucial for accurately modelling pathological conditions such as cancer and fibrosis, where disrupted cell-to-cell interactions play a fundamental role in disease progression [292]. Despite their potential, challenges remain in controlling the spatial patterning of spheroids across larger tissue structures and in replicating the heterogeneity required for functional tissue engineering. Traditionally, biofabrication technologies have relied on embedding cells within hydrogels, which restricts direct cell-cell interactions and results in low-density constructs. To overcome these limitations, 3D bioprinting approaches have been developed to facilitate the fusion of spheroids into larger tissue strands, which can then be extruded through a microcapsule to create more complex and functional tissue architectures.

A comparative analysis of microfluidic and 3D printing techniques for cell encapsulation reveals distinct advantages and limitations across several key parameters. Microfluidic systems offer exceptional control over droplet size and monodispersity, with encapsulation efficiencies typically exceeding 90 % for single-cell encapsulation. These systems are well-suited for capsule production, making them ideal for applications that require a uniform size. However, the initial setup cost of microfluidic platforms ranges from $5000 to $20,000, depending on performance and functionality. Moreover, microfluidic techniques are compatible with suspension and non-adherent cell types, such as hematopoietic stem cells and immune cells [305].

In contrast, 3D bioprinting enables the fabrication of microcapsules, as well as customizable 3D structures with greater flexibility in spatial patterning and cell distribution. Depending on the printing modality, encapsulation efficiencies typically range between 70 % and 85 %. Additionally, 3D bioprinting supports a broader range of biomaterials, including higher-viscosity hydrogels, which are more suitable for adherent cells like fibroblasts, mesenchymal stem cells, and epithelial cells. However, 3D bioprinting generally offers lower throughput and longer fabrication times, with the production of large tissue constructs potentially requiring several hours. The cost of bioprinters varies widely, ranging from approximately $10,000 to over $100,000, depending on resolution and functionality. In contrast, both technologies offer high precision and customizability, but also have certain limitations. Despite producing highly uniform capsules, microfluidic encapsulation can have scalability issues, particularly in maintaining sterility. Moreover, 3D printing technique more suitable for designing intricate, tissue-like architectures suitable for tissue engineering applications, but face limitations in printing resolution, material biocompatibility, and the risk of cell damage due to shear stress during extrusion [306]. Among the common challenges for both methods are achieving adequate vascularization within constructs, an essential factor for long-term cell survival and function. Ultimately, the choice of microfluidics and 3D printing depends on the specific application requirements, whether high-throughput uniform encapsulation or complex tissue architecture. Together, these developments represent a significant advancement in regenerative medicine and targeted therapeutic delivery.

#### 2.2.3 In-situ preparation

The in-situ preparation of cell capsules creates a protective environment around live cells directly at the targeted site. This method combines cells with a biomaterial that polymerizes or gels upon injection, stable within the permeable matrix. In-situ encapsulation offers several advantages: it minimizes cell handling and mechanical stress, ensures immediate compatibility with the surrounding environment, and allows for precise control over cell placement [84,85]. These approaches are used to enhance cell viability and functionality, enabling encapsulated cells to adapt smoothly to physiological conditions and improving integration with therapeutic effectiveness. However, challenges remain, such as ensuring that the biopolymer material achieves uniform encapsulation with long-term stability, supports cell viability and functionality, and maintains biocompatibility [86]. The in-situ polymerization process is carefully engineered to create a porous membrane that allows for the exchange of oxygen and nutrients while protecting encapsulated cells from immune system attacks after transplantation. Cell encapsulation methods are generally categorised into macro and micro platforms. The macro platform methods use large-scale devices to encapsulate cells within hollow fibers or bulk hydrogels, while micro-platform methods involve microparticles or microfibers, often prepared in situ. It could be encapsulated cells that deliver therapeutic agents in a controlled manner and serve as functional tissue constructs, essential for repairing or replacing damaged tissues and organs [84]. Besides, *in-situ* cell encapsulation is an innovative technique with significant potential to enhance the biological benefits of therapeutic applications.

#### 2.2.4 Electrospraying and electrostatic self-assembly

Electrospraying and electrostatic self-assembly methods have emerged as platforms for cell encapsulation technology, each method offering unique advantages. Electrospraying, also known as electrohydrodynamic atomization, is a multipurpose technique for generating micro-to nanoscale capsules. Using coaxial or triple-coaxial configurations, multi-layered capsules can be fabricated with flexibility for various therapeutic applications [11]. This method achieved precise size control, reduced reagent consumption, and greater efficiency in droplet formation. This method leverages the scalability of biomaterial capsule production, ensuring high uniformity and suitability for a large-scale manufacturing process. The reproducibility of this method ensures that various parameters, including voltage, flow rate, and nozzle size, as well as environmental conditions such as humidity and temperature, are securely regulated. In contrast, the electrostatic self-assembly method is typically achieved through layer-by-layer (LbL) deposition, which allows nanoscale precision over capsule thickness, porosity, and permeability, thereby regulating nutrient diffusion, oxyge transport, and immune isolation. Conducted in aqueous buffers under physiological conditions, LbL assembly minimizes cellular stress and preserves the viability of various populations, including stem cells, pancreatic islets, and probiotic bacteria. Both electro-spraying and electrostatic self-assembly methods are highly suitable for clinical translation because they enable the design of customizable encapsulation systems [12]. Moreover, electro-spraying has been applied in cartilage and bone regeneration, wound healing, and sustained delivery of bioactive molecules. At the same time, electrostatic self-assembly has shown promise in enhancing graft survival, providing immunoprotection, and reducing fibrotic encapsulation of implants. Together, these approaches offer scalable, reproducible, and versatile solutions with significant potential in regenerative medicine, cell therapy, and implantable medical devices.

## 3 Biomaterials

**Fig. 6:**
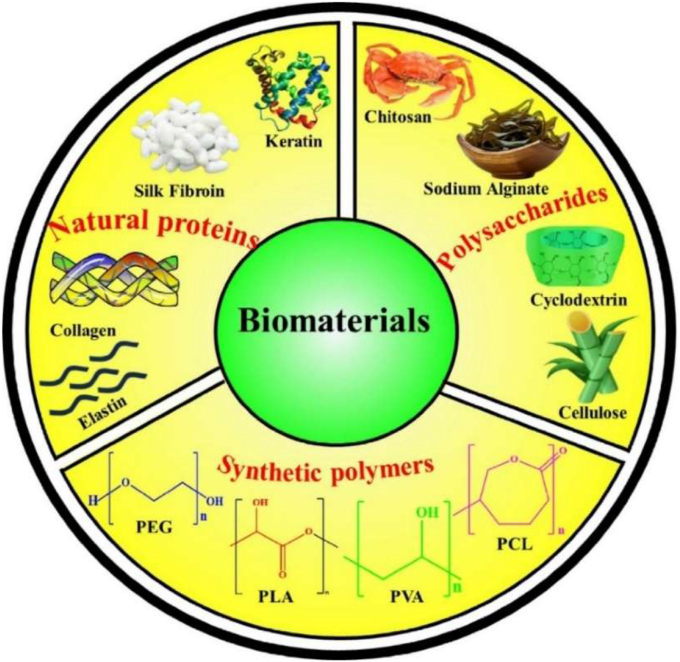
Different types of biomaterials including natural proteins, polysaccharides, and synthetic polymers.

**Fig. 7:**
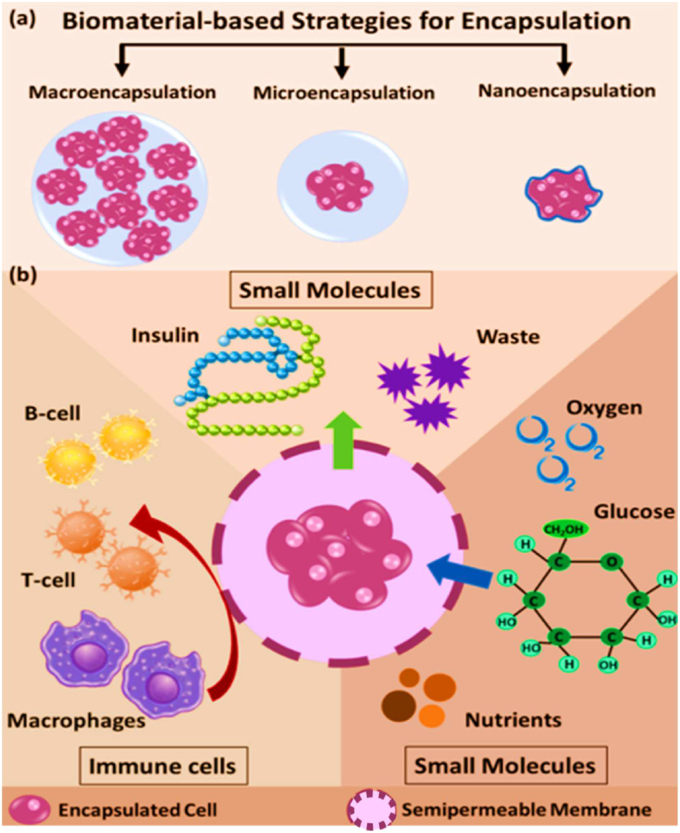
(a&b) Biomaterial-based different sizes of cell encapsulation such as macroencapsulation, microencapsulation, nanoencapsulation, and small molecules interact with cell encapsulation. Reproduced from Ref. [252] Copyright 2023 Elsevier.

Biomaterials are essential for preserving cell function and ensuring survival in the host environment [98]. They are used for therapeutic purposes, provide robust support for encapsulated cells, enable the controlled release of bioactive molecules, and protect cells from immune rejection while maintaining their functionality [94]. The different types of biomaterials are denoted as natural proteins, polysaccharides, and synthetic polymers (Fig. 6). These biomaterials significantly enhance the cellular microenvironment and serve as platforms for drug delivery, tissue engineering, and cell therapy [96,97]. Protein-based materials (e.g., collagen, gelatin, silk protein, keratin, and elastin) are increasingly used to mimic the natural extracellular matrix (ECM), enhancing biocompatibility and supporting proper cell growth and functionality. Polysaccharide-based materials (e.g., sodium hyaluronate, sodium alginate, chitosan, cellulose, and cyclodextrin) exhibit excellent properties, including biocompatibility, biodegradability, and the ability to form hydrogels with high mechanical strength. Synthetic polymer-based materials (e.g., polyethylene glycol (PEG), polylactic acid (PLA), polyvinyl alcohol (PVA), and polycaprolactone (PCL)) offer precise control over mechanical properties, degradation rates, and functionality, making them versatile for diverse applications. Moreover, these biomaterials not only provide structural support for encapsulated cells but also enable the sustained and controlled release of bioactive molecules [[99], [100], [101]]. Biomaterial-based different sizes of cell encapsulation such as macroencapsulation, microencapsulation, and nanoencapsulation (Fig. 7a). The small molecules interact with cell encapsulation (Fig. 7b). The summarises of various biomaterial-based cell encapsulation strategies, including their applications, advantages, and challenges (Table 1). Comparison of different biomaterials that regulate cell type, proliferation, differentiation, function, and multiple approaches (Table 2). This review highlights the potential of biomaterial-supported cell encapsulation, offering new possibilities for efficient and innovative treatments in regenerative medicine. Overall, biomaterial capsules have been employed in various strategies to enhance cell survival, functionality, and integration across a wide range of biomedical applications.

**Table 1:** Biomaterials-based different strategies of cell encapsulation methods, applications, advantages and challenges.

Categories	Types of Materials	Encapsulation Method	Applications	Advantages	Challenges	Refs
Natural Proteins	Collagen, Gelatin,	Droplet-based encapsulation	Regenerative medicine,	Excellent biocompatibility and biodegradability,	Limited mechanical strength	[108,112,120]
	Silk fibroin, keratin	Biofabrication of matrices with direct assembly in the cells	Cancer therapy, Wound healing	Promote cell adhesion and proliferation, Support natural extracellular matrix		
	Elastin					
Polysaccharides	Sodium hyaluronate,	Composite encapsulation methods (e.g. Microfluidics	Immune isolation, drug delivery	Biocompatible and biodegradable, supports nerve regeneration and hydrophilicity	Rapid degradation in physiological conditions	[139,142,151]
	Sodium alginate, Chitosan, Cellulose, Cyclodextrin	3D Printing, *In Situ* Preparation)				
Synthetic polymers	Polyethylene glycol, Polylactic acid, Polyvinyl alcohol, Polycaprolactone	Chemical or radical polymerization techniques	Controlled release systems, Tissue engineering	Tunable mechanical and degradation properties, Scalable production, Can be modified for controlled drug release, long-term stability	Potential cytotoxicity, lack of bioactivity	[[158], [159], [160], [161], [162], [163], [164]]
Hybrid Biomaterials	Hydrogel-polymer composites	Composite encapsulation	Multifunctional scaffolds, Drug delivery	Synergistic properties, Versatility	Complex fabrication cost efficiency	[131]
Microcapsules	Natural proteins, Polysaccharides, and Synthetic polymers	Physical methods (spray-drying and freeze-drying), Physicochemical methods (complex coacervation, ionic gelation and electrostatic layer-by-layer deposition), Chemical methods (interfacial polymerization)	Cancer therapy, Controlled drug/cell delivery Tissue regeneration Wound healing	High surface-to-volume ratio, Scalability	Size control, Burst release risks	[64,73]
3D Printing	Natural and Synthetic polymer	Layer-by-layer extrusion	Personalised medicine for all biomedical industry	Precise control, Customizable designs	Printing resolution, Cell viability	[79]
Hydrogel-Based Encapsulation	Natural proteins, polysaccharides, and synthetic polymers	Droplet-based encapsulation of physical and chemical method	Cell therapy	Biocompatibility, Tunable properties	Immune rejection, Limited mechanical strength	[93]
Bioactive smart Biomaterials	Hydrogel, Scaffolds, Nanofiber, Nanofilm, Microsphere, etc	Electrospining, Solution casting, 3D printing, and Freeze-drying	Tissue regeneration, Cancer therapy	Cell adhesion, Biofunctionality	Degradation kinetics, Immune response	[97]
Functionalization of bioactive Materials	Stimuli-responsive polymers (e.g., pH and temperature)	*In situ* gelation, Responsive coatings	Targeted delivery, Dynamic therapies	Adaptive behaviour, Specific targeting	Complexity, Limited clinical validation	[98]

**Table 2:** Biomaterials comparison of cellular regulation of cell type, cell proliferation, cell differentiation, cell function and approaches.

Materials	Cell types	Cell Proliferation	Cell Differentiation	Cell Function and Applications	Approach	Refs
Silk Fibroin	Mammalian cells	High, supports adhesion	Induce osteogenesis and neurogenesis	Supports ECM-like functions	Blending with polymers, functionalization with RGD peptides	[104]
Collagen	Mammalian cells	Excellent, mimics ECM	Enhances mesenchymal stem cell (MSC) differentiation	Promotes tissue regeneration	Cross-linking, electrospinning, enzyme treatment	[110]
Gelatin	Mammalian cells	Biocompatible	Supports chondrogenic and osteogenic differentiation	Enhances wound healing	Cross-linking with genipin, blending with growth factors	[113]
Keratin	Mammalian cells	Supports cell adhesion	Affects nerve and skin cell differentiation	Promotes wound healing and neural repair	Functionalization with bioactive peptides	[117]
Elastin	Mammalian cells	Moderate, provides elasticity	Assists endothelial and vascular cell differentiation	Enhances elasticity for vascular tissues	Cross-linking, copolymerization with collagen	[123]
Chitosan	Mammalian cells, bacteria	Antibacterial, supports cell attachment	Encourages osteogenic and chondrogenic differentiation	Accelerates wound healing	Chemical grafting, ionic cross-linking	[131]
Sodium Alginate	Mammalian cells, bacteria,	Low, requires modification for adhesion	Used for cartilage and pancreatic cell differentiation	Supports hydrogel-based drug delivery	Ionic gelation, blending with gelatin or collagen	[136]
Sodium Hyaluronate	Mammalian cells	Enhances cell viability and migration	Influences stem cell differentiation	Aids in wound healing and lubrication	Chemical cross-linking, incorporation into hydrogels	[142]
Cellulose	Mammalian cells, or Plant	Low, needs modification	Minimal direct differentiation influence	Supports mechanical integrity	Surface modification, oxidation	[146]
Cyclodextrin	Mammalian cells, bacteria,	Low, primarily used for drug delivery	Minimal direct differentiation influence	Functions as a controlled drug carrier	Functionalization with bioactive molecules	[152]
Polyethylene Glycol (PEG)	Synthetic Extracellular Matrix	Anti-fouling, limits protein adhesion	Poor, typically inert	Used for drug delivery	Grafting bioactive peptides, copolymerization	[156]
Polylactic Acid (PLA)	Bacteria	High, supports cell growth	Induces osteogenic differentiation	Bone and tissue engineering	Blending with bioactive ceramics, surface modification	[162]
Polyvinyl Alcohol (PVA)	Synthetic Extracellular Matrix	Biocompatible, supports adhesion	Limited differentiation support	Hydrogels for controlled release	Cross-linking, blending with collagen	[164]
Polycaprolactone (PCL)	Synthetic Extracellular Matrix	Moderate, supports long-term adhesion	Osteogenic and nerve differentiation	Used in scaffolds for tissue regeneration	Surface etching, blending with natural proteins	[172]

### 3.1 Protein based materials

#### 3.1.1 Silk fibroin

**Fig. 8:**
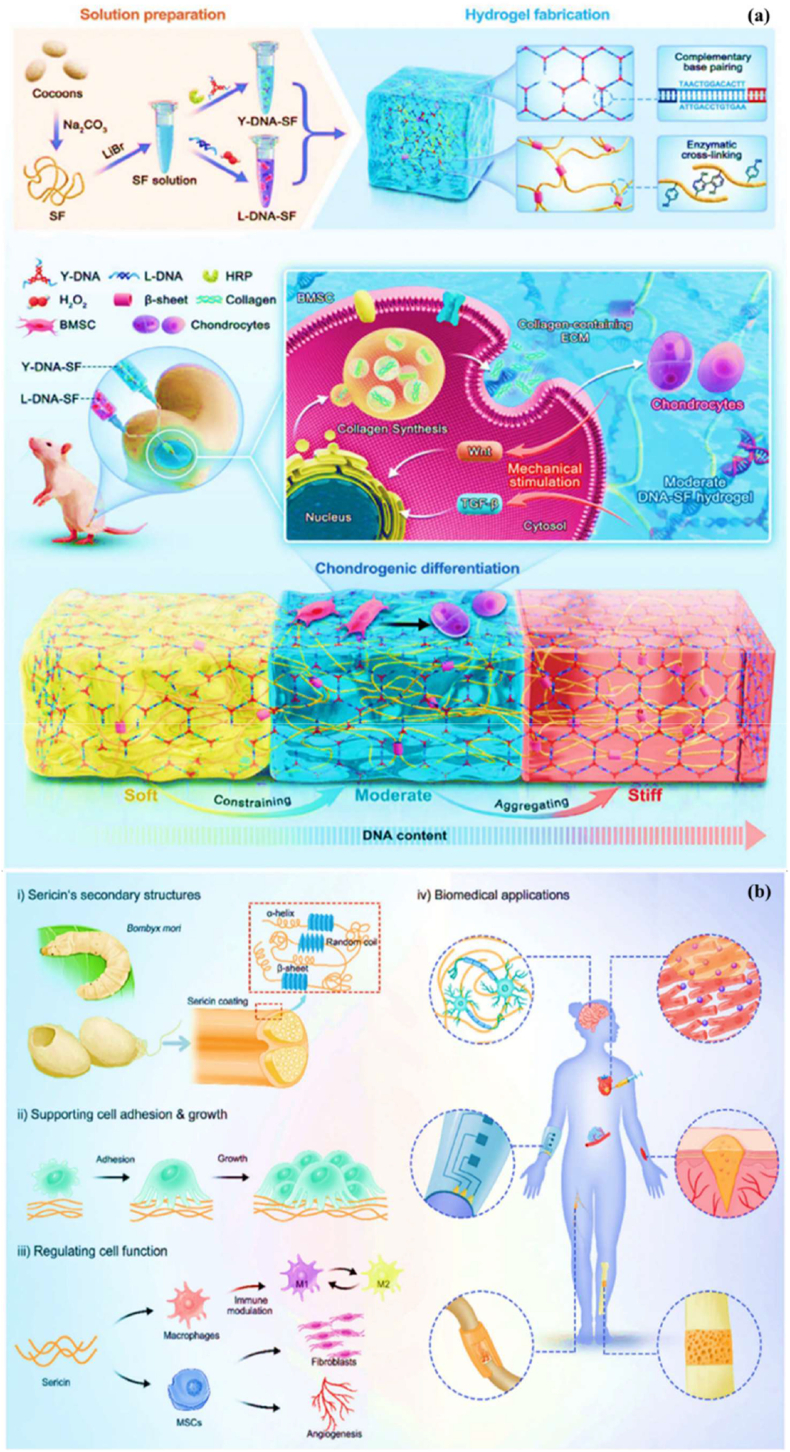
(a) Silk fibroin-based design of dual-network hydrogels with tunable surface rigidity for controlling chondrogenic differentiation in cartilage defect repair with DNA content of soft moderate, and stiff. Reproduced with permission from Ref. [254], Copyright 2024, Wiley-VCH. (b) Sericin-based biomaterial: (i) silk sericin structure of α-helix, β-sheet, and random coil, (ii) cellular adhesion and growth behaviour, (iii) regulation of cell function, and (iv) biomedical applications. Reproduced with permission from Ref. [255], Copyright 2024, Wiley-VCH.

The silk fibroin is a protein-based biomaterial obtained from the *Bombyx mori* silkworm. It consists of two distinct layers: the inner layer, known as fibroin, and the outer layer sericin. Silk fibroin-based design of dual-network hydrogels with tunable surface rigidity for controlling chondrogenic differentiation in cartilage defect repair with DNA content of soft, moderate, and stiff (Fig. 8a). It consists of various amino acids, including glycine, alanine, and serine, which enhance the biological properties, also suitable for textiles, biomedicine, cosmetics, and food industries [102]. The silk fibroin is composed of two subunits: light (L) chains, known as silk I, with a molecular weight of 27 kDa, and heavy (H) chains, referred to as silk II, with a molecular weight of 391 kDa [103,104]. These components combine to form complexes of heavy and light chains arranged in anti-parallel β-sheet structures that are insoluble in water due to their hydrophobic properties [105]. The light chains are covalently linked to the heavy chains through disulfide bridges, which prevent their retention within the endoplasmic reticulum. In the light chains have been unique, non-repetitive sequences, while the heavy chains primarily consist of β-sheets that align with the fiber matrix. The specific interactions of these β-sheets within the crystalline regions influence important material properties, such as nanocrystalline size, intercrystallite distances, and crystallite arrangement [106]. Moreover, silk fibroin contains a range of chemical groups (e.g., amide-I, amide-II, amide-III, alcohols, carboxyls, and thiols), allowing it to interact with biomolecules or antibodies specific to certain cells. The sericin-based biomaterial: (i) silk sericin structure of α-helix, β-sheet, and random coil, (ii) cellular adhesion and growth behaviour, (iii) regulation of cell function, and (iv) biomedical applications as displayed (Fig. 8b). The silk fibroin exhibits low immunogenicity, reducing the risk of adverse reactions. Its customizable properties, such as pore size, degradation rate, and mechanical strength, make it an ideal material for various wound care and skin regeneration applications, regulating moisture and maintaining an optimal environment for tissue regeneration by mimicking the extracellular matrix (ECM) [107,108]. Besides, the key properties of silk fibroins are promising biomaterials for bone tissue engineering and wound healing applications.

#### 3.1.2 Collagen

**Fig. 9:**
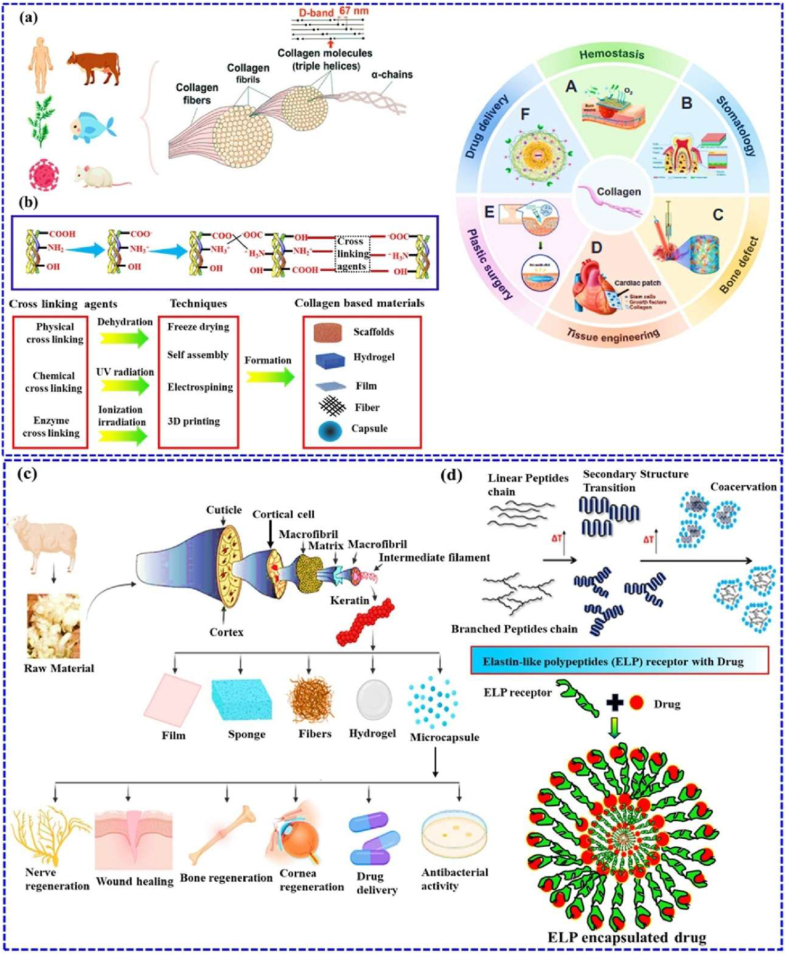
(a&b) Sources and structural characteristics of collagen with the cross-linking process and biomedical applications. Reproduced with permission from Ref. [253], copyright, 2022 Wiley-VCH. (c) Keratin-based different materials designed for tissue engineering applications Reproduced from Ref. [256] Copyright 2022, Elsevier. (d) Elastin-like polypeptide (ELP) illustration of linear and branched peptides for secondary structure transition (intramolecular process) followed by coacervation (intermolecular process) with encapsulated drug. Reproduced with permission from Ref. [257] copyright 2016 ACS.

Collagen is the most abundant protein biomaterial found in animal tissues such as skin, bone, ligaments, cartilage, and various organs [109]. It plays a crucial role in maintaining the structural integrity of tissues. The collagen molecules are presented in various amino acids, and molecular weight of approximately 300 kDa. It is classified into different types. All collagen is a triple-helical structure consisting of three polypeptide α-chains [110]. This triple helix is stabilised by intramolecular and intermolecular covalent bonds, particularly at the C-terminus and N-terminus of the α-chains, which strengthen the collagen fiber matrix. The molecular composition of different amino acids, such as glycine, proline, hydroxyproline, and alanine [111]. These amino acids are crucial for contributing to the structural stability and functional properties of the biomedical fields. Schematic diagram of collagen sources and structural characteristics of the cross-linking process and biomedical applications (Fig. 9 a&b). Type I collagen is also considered a glycoprotein, although its carbohydrate content is relatively low, constituting less <1 %. The type I collagen is highly valued in regenerative medicine due to its excellent biocompatibility and ability to support tissue regeneration. As the primary component of the extracellular matrix in both soft and hard tissues, collagen plays a crucial role in regulating cellular behaviour and maintaining the extracellular microenvironment. Collagen derivatives from gelatin molecules, which support cell adhesion and proliferation [112,113]. It can be processed into various forms, including porous sponges, gels, and fibers, each form offering unique benefits for tissue engineering. These scaffolds promote rapid tissue synthesis and reorganisation during implantation. Naturally, collagen exhibits superior biological properties, including hydrophilicity, low antigenicity, and flexibility, which facilitate the delivery of nutrients or drugs, making it an ideal material for tissue regeneration and wound healing [114]. However, collagen has limited mechanical strength and rapid degradation. To address these limitations, cross-linking techniques have enhanced mechanical strength and stability while improving multiple performances in biomedical applications.

#### 3.1.3 Keratin

Keratin is a protein biopolymer derived from animal hair and nails in their epithelial cells. It's crucial for maintaining structural integrity and supporting various metabolic activities [115]. Keratin-based different materials designed for tissue engineering applications (Fig. 9c). Recently, keratin has gained significant attention in biomedical engineering due to its excellent biocompatibility and biodegradability. The cellular interactions are attributed to specific amino acid sequences within the keratin molecule, also present in the extracellular matrix (ECM) [116]. The keratin differential has two types: acidic medium, represented by type I keratin, and basic medium, represented by type II keratin. In this regard, the secondary structure of keratin is subdivided into α-keratin, β-keratin and γ-keratin. These structures form various shapes, such as polypeptide chains, filament matrices, and sandwich patterns. This matrix contributes to the nail plate's transparent keratinocytes [117]. The acidic α-keratin contains cell-binding motifs, including arginine, glycine, aspartic acid, and leucine-aspartic acid-valine. These motifs are similar to those found in ECM proteins like collagen and fibronectin, and interaction with integrins that stimulate cellular attachment, proliferation, and migration. It is very crucial for the functioning of various cell types, including microvascular endothelial cells, keratinocytes, and fibroblasts [118]. Keratinocytes comprise most epidermal cells and are responsible for the skin's toughness. These cells are obtained in keratin, facilitating cell development and enhancing the skin's ability to protect against bacterial pathogens. The cytoskeleton of epithelial cells consists of microfilaments, microtubules, and intermediate filaments. Approximately 26 % of the genes involved in keratin synthesis are actively expressed in epithelial cells. Each filament type has distinct physicochemical properties, providing excellent cellular interaction and biodegradability [119]. The outer layer of the skin, composed primarily of keratinised cells, forms a nearly impenetrable barrier that protects the body from external infections. Likewise, keratin-based biomaterials are increasingly used in biomedical applications due to their favourable properties.

#### 3.1.4 Elastin

Elastin, derived from tropoelastin, is a crucial component of the elastin-like polypeptide chain of the extracellular matrix (ECM), providing high mechanical strength and extraordinary elasticity. It's a chemically stable and hydrophobic protein with a molecular weight of 67 kDa. The process of elastin formation, known as elastogenesis, begins with the intracellular synthesis of tropoelastin. Elastin-binding protein (EBP) transports tropoelastin to specific sites on the cell surface, preventing premature aggregation and degradation [120]. Elastin-like polypeptide (ELP) illustration of linear and branched peptide chains for secondary structure transition (intramolecular process), followed by coacervation (intermolecular process) with encapsulated drug (Fig. 9d). On the cell surface, galactosugars on microfibrils bind to the lectin-binding site of EBP, releasing tropoelastin into the extracellular environment. There, it undergoes cross-linking and polymerization to form the elastin matrix. In the past two decades, elastin has gained recognition for its unique biomedical properties, especially in dynamic tissues such as the skin, lungs, and arteries [121]. It also plays a vital role in tissue regeneration, often by incorporating exogenous elastin to stimulate endogenous production, thereby supporting tissue repair [122]. However, adult cells are unable to assemble elastic fibers after the neonatal period, thereby reducing elastogenesis. This deficiency impairs tissue repair; elastin is required for maintaining tissue structure and function replenished during the repair process. In addition, elastin plays a structural role in regulating cellular signalling, chemotaxis, proliferation, and proteinase release [123]. These regulatory effects extend to various immune cells, including monocytes, macrophages, neutrophils, and lymphocytes, underscoring elastin's multifunctional role in maintaining tissue integrity and regulating the immune response.

#### 3.1.5 Gelatin

Gelatin is a natural protein biopolymer from denatured collagen in bovine and pig skin. The gelatin is obtained in two different types, such as Type A and Type B. The gelatin comprises several amino acids, the most common of glycine, proline, and hydroxyproline [124]. Other amino acids found are glutamic acid, alanine, arginine, and aspartic acid, connected to the hydrophilic chains. Its structure also covers various polypeptide chains contributing to its unique chemical properties. In the 19th century, the pharmaceutical industry introduced and widely adopted gelatin capsules. Gelatin offers several advantages in food packaging and pharmaceuticals due to its biocompatibility, biodegradability, non-toxicity, and ecological sustainability [[125], [126], [127]]. Likewise, gelatin could be easily crosslinked to integrate other substrates for enhancing the biological properties. It can be processed into various forms, including hydrogels, films, scaffolds, nanofibers, and microspheres, making it adaptable for numerous applications such as gene delivery, drug delivery, interventional therapy, and targeted tumor therapy. Moreover, gelatin supported different superior properties in biomedical applications due to its structural similarity to the native extracellular matrix. The polypeptide chains in gelatin contain sequences, such as arginine-glycine-aspartic acid, which promote cell growth and regulate enzyme degradation, thus supporting tissue regeneration [128]. In pharmaceutical fields, gelatin is a key matrix in intravenous infusions, injectable drug delivery microspheres, and implants. It also plays a crucial role in enhancing bone health and joint function when consumed orally. In hemostasis, gelatin helps control bleeding by providing a scaffold for fibrin clots, restricting blood flow, and forming a stable matrix around the injury. For example, authors Asim et al. discussed the development of multi‐functional gelatin dithiolane hydrogels for therapeutic potential applications [129]. These hydrogels enhance cell therapy for deep wound treatment by delivering cells into deeper tissue layers via a microsyringe needle. This approach reduces invasiveness and minimizes targeted side effects, making it a promising solution for advanced wound care applications.

### 3.2 Polysaccharide materials

#### 3.2.1 Sodium hyaluronate

**Fig. 10:**
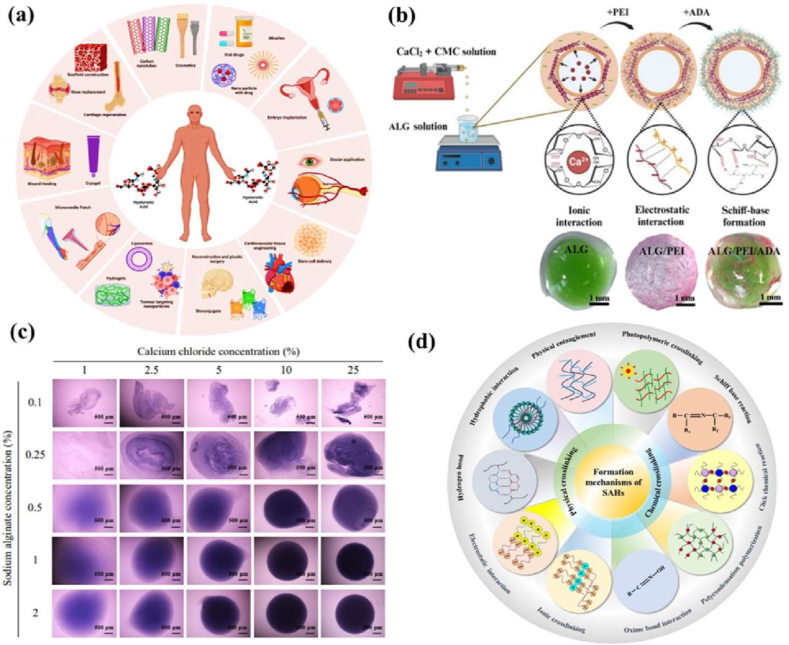
(a) Schematic diagram of hyaluronic acid applications in various fields. Reproduced from Ref. [258], Copyright 2024, Springer. (b) Schematic diagram illustrating the aqueous core-shell capsule formation process, including the cross-linking mechanism and covalent and non-covalent bonds between alginate (ALG), polyethyleneimine (PEI), and alginate dialdehyde (ADA). Light microscopy images of the liquefied capsules are shown the core stained green, ALG and ADA layers being transparent and colourless, and the PEI layer stained pink. Reproduced with permission from Ref. [259], Copyright 2024, ASC. (c) Calcium chloride concentrations of 1 %, 2.5 %, 5 %, 10 %, and 25 % were combined with sodium alginate concentrations of 0.1 %, 0.25 %, 0.5 %, and 1 %. For enhanced visualization, a 0.5 % trypan blue solution was mixed to find the capsule degradation morphology. Fluorescence imaging was then performed on alginate capsules encapsulating 5 × 10^3^ DiI-labelled C3H10T1/2 cells. Scale bars 500 μm. Reproduced from Ref. [260], Copyright 2023, Appl. Sci. (d) Sodium alginate formation of hydrogel physical and chemical cross-linking of various approaches. Reproduced with permission from Ref. [261], Copyright 2025, Elsevier.

Sodium hyaluronate is a derivative of hyaluronic acid, naturally found in the human body, particularly in connective tissues, bones, and skin [140]. Its chemical structure consists of a long-chain polymer made up of repeating disaccharide units containing glucuronic acid and N-acetylglucosamine. These units are linked by alternating β-1,3 and β-1,4 glycosidic bonds, giving them high water solubility and lubricating properties. Beyond its role in hydration, sodium hyaluronate is essential in the pericellular matrix of certain cells. This matrix is crucial for various biochemical interactions, such as cell signalling, adhesion, and immune modulation [141]. The pericellular region, or cell capsule, contains high concentrations of hyaluronic acid, forming a viscoelastic matrix around the cell surface. Additionally, sodium hyaluronate's ability to bind water molecules and cross-link with other matrix proteins, like aggrecan and collagen, helps protect cells. It also maintains osmotic balance, provides mechanical support, and facilitates tissue regeneration. The biological effects of sodium hyaluronate depend on its molecular weight. High molecular weight hyaluronate plays a critical role in the early stages of wound healing by regulating the migration of inflammatory cells and fibroblasts. In contrast, lower molecular weight hyaluronate promotes leukocyte chemotaxis and cytokine production as healing progresses, further enhancing tissue repair [142]. For example, hyaluronic acid injections are used to restore joint lubrication and relieve symptoms of osteoarthritis by mimicking the natural capsule surrounding synovial cells. Additionally, drug delivery systems utilising nanocarrier-based cell capsules improve the targeted delivery of drugs to cancer cells or inflamed tissues. Sodium hyaluronate is also used as a hydrogel, simulating the pericellular environment to promote cell proliferation and matrix deposition, aiding tissue regeneration. Schematic diagram of hyaluronic acid applications across various fields (Fig. 10a). They possess unique properties, including viscoelasticity, biocompatibility, biodegradability, non-immunogenicity, and water retention, making them an invaluable natural polyelectrolyte in numerous bio-industrial applications. These properties facilitate cell signalling pathways and support interactions with specific receptors involved in cell adhesion and cell-matrix interactions. Moreover, sodium hyaluronate is susceptible to degradation by hyaluronidase enzymes and reactive oxygen species, which help regulate its biological functionality.

#### 3.2.2 Sodium alginate

Sodium alginate is a natural polysaccharide derived from brown algae, distinguished by a linear structure composed of repeating units of β-D-mannuronic acid and α-L-guluronic acid. Its unique properties, including excellent biocompatibility, optimal biodegradability, pH sensitivity, non-toxicity, non-immunogenicity, and affordability, make it a highly attractive polymer for various biomedical and pharmaceutical applications, particularly in controlled drug release [136]. This biopolymer backbone is rich in functional groups, such as carboxyl and hydroxyl, which contribute to its ability to undergo hydrophobic modifications. These functional groups also enable the formation of intra and intermolecular hydrogen bonds, playing a crucial role in influencing the material's solubility, viscosity, and crystallinity [137]. Schematic diagram of the alginate with polyethyleneimine used in the aqueous medium for a core-shell capsule (Fig. 10b). These capsules are formed through ionic gelation, where sodium alginate is mixed with a solution containing divalent cations. The resulting gel-like structure encapsulates cells, providing a protective barrier that can be used in various applications. The fluorescence imaging of alginate capsules encapsulating 5 × 10^3^ DiI-labelled C3H10T1/2 cells for capsule degradation morphology (Fig. 10c). Alginate hydrogel formation of physical and chemical cross-linking using various approaches (Fig. 10d). The sodium alginate capsules have proven particularly useful in cell encapsulation, as they create a stable microenvironment that supports cell survival while being biocompatible and biodegradable. The functional groups of sodium alginate also serve as active sites for attaching various side chains, which can be introduced under mild conditions. This ability to retain functional groups allows sodium alginate to interact with biomolecules and cells, facilitating controlled drug release and promoting tissue regeneration. Recent studies have highlighted the positive effects of sodium alginate on *in vitro* cell growth, demonstrating that it significantly influences cellular behaviours such as proliferation, migration, and differentiation. The cells encapsulated in sodium alginate capsules can convert external physical stimuli from their environment into biochemical signals, activating genetic programming and cell function. Also, this ability responds to environmental cues, making sodium alginate an excellent material in cell-based therapies and tissue regeneration. It is commonly incorporated into immediate-release tablets, serving multiple roles, including as a suspending agent, tablet binder, and controlled drug release [138]. Additionally, in soft tablets, sodium alginate functions as an elastically deforming excipient, improving the performance of sensitive drugs and showing great promise in various biomedical and pharmaceutical applications [139].

#### 3.2.3 Chitosan

**Fig. 11:**
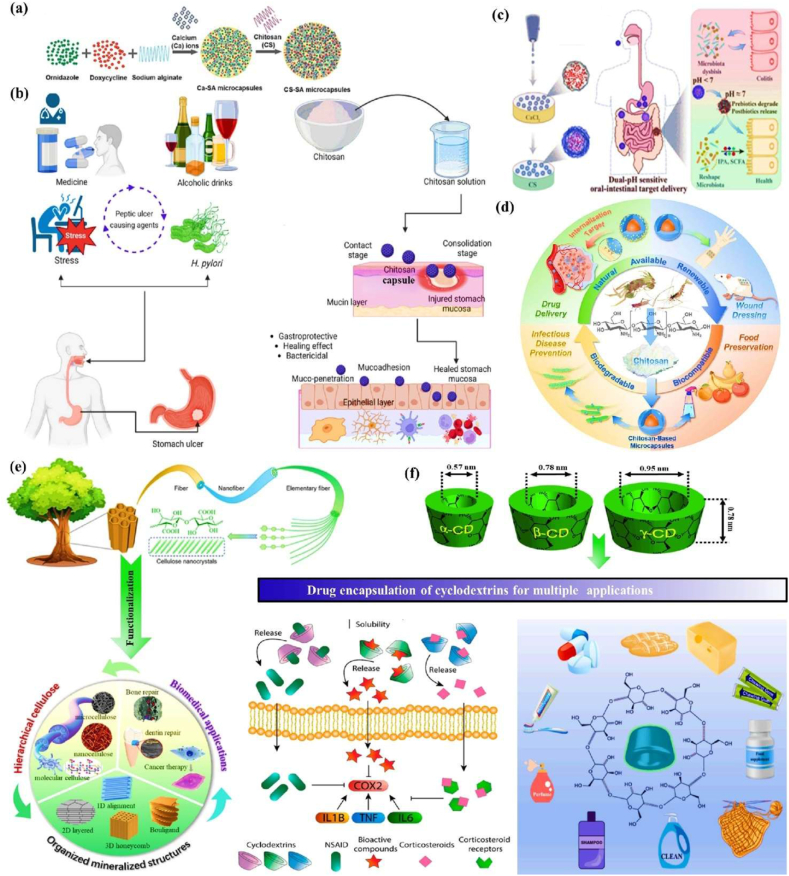
(a) Schematic diagram of the preparation of microcapsules using alginate-chitosan loaded with ornidazole and doxycycline drug molecules. Reproduced with permission from Ref. [262], Copyright 2023, Elsevier. (b) Chitosan capsule directly or indirectly treats the stomach gastric with mucoadhesive properties. Reproduced with permission from Ref. [263], Copyright 2024, Elsevier. (c) Chitosan used prebiotics encapsulation of postbiotic microcapsules for preventing and treating colitis in dual pH-sensitive oral targeted drug delivery. Reproduced with permission from Ref. [262], Copyright 2023, Elsevier. (d) Chitosan-based microcapsule applications in multiple fields. Reproduced with permission from Ref. [262], Copyright 2023, Elsevier. (e) Overview of cellulose structures with functionalization of different processes. Reproduced from Ref. [264], Copyright 2023, RSC. (f) The cyclodextrin structure, applications, and bioactive compounds of anti-inflammatory drugs encapsulated in the cyclodextrin molecules. Reproduced with permission from Ref. [265], Copyright 2024, Elsevier, and Reproduced with permission from Ref. [266], Copyright 2021, Biomolecules.

Chitosan is a semi-crystalline, cationic biopolymer with a linear structure composed of glucosamine and N-acetylglucosamine-linked polysaccharide units [130]. It is derived from chitin through an alkaline deacetylation process. Schematic diagram of the preparation of microcapsules using alginate-chitosan loaded with ornidazole and doxycycline drug molecules (Fig. 11a). Although chitosan-based drug delivery systems often lead to rapid drug release, which contrasts with the desired sustained release, chitosan retains several favourable biological characteristics. Chitosan capsule directly or indirectly treats the stomach and the gastric mucosa with mucoadhesive properties (Fig. 11b). Its biomimetic structure and functional groups impart osteogenic properties and antibacterial activity [131]. Chitosan is used for encapsulating prebiotics and postbiotic microcapsules for preventing and treating colitis, with dual pH sensitivity for oral targeted drug delivery (Fig. 11c). Moreover, chitosan mimics glycosaminoglycans in the extracellular matrix and demonstrates potential antimicrobial, hemostatic, and antioxidant effects. Nowadays, chitosan-based composite matrices have been shown to exhibit enhanced biological activity, including high biodegradability, free radical scavenging, interactions with bacterial cell walls, promotion of cell proliferation, and biocompatibility across various formulations. Chitosan-based microcapsule applications in multiple fields (Fig. 11d). Several biological mechanisms supported the chitosan molecules. First, the electrostatic interaction of amino groups with negatively charged microbial cell membranes leads to intracellular leakage and eventual cell death [132]. Second, low-molecular-weight chitosan can penetrate microbial cells due to its small size, disrupting cellular processes by interacting with anionic components such as nucleic acids and proteins, thereby supporting the metabolism [133]. Third, chitosan acts as a chelating agent, binding essential nutrients and making them unavailable to fungi, inhibiting their growth [134]. Finally, chitosan is regulated to form a nutrient and oxygen barrier, as well as through its interaction with DNA, allowing it to penetrate fungal cell walls and interfere with mRNA synthesis, thereby inhibiting the production of essential proteins and enzymes [135].

#### 3.2.4 Cellulose

Cellulose is the most abundant natural polymer derived from plant materials. It can be obtained from various sources, such as wood, plants, and microbes. Structurally, cellulose is a complex polymer consisting of linearly connected D-glucose molecules linked by β-1,4-glycosidic bonds. The hydrogen bonding between cellulose chains creates regions of both high crystallinity and amorphous areas, imparting strong mechanical stability and chemical resistance. These properties, along with high crystallinity and robust hydrogen bonding, make cellulose insoluble in water and most organic solvents. The cellulose structure contains hydroxyl, aldehyde, and carboxyl groups, which enhance its functional properties, mechanical strength, and high surface area [[143], [144], [145]]. It also exhibits excellent biocompatibility and biodegradability, making it suitable for a range of biological applications. For example, cellulose-based materials are used in cell encapsulation to protect mammalian cells from the immune system. The overview of cellulose structures with functionalization of different processes (Fig. 11e). On the pharmaceutical side, cellulose is essential for forming oral tablets with drug-loaded matrices. Additionally, cellulose is utilized in tissue regeneration, where three-dimensional structures support new tissue growth. Otherwise, cellulose materials play a critical role in tissue engineering and regenerative medicine, offering a scaffold for cell growth, differentiation, and tissue formation, while providing enhanced structural support and biological activity. They are widely used to repair or replace damaged tissues and organs, such as bone, cartilage, skin, and muscle [146]. In this context, bacterial cellulose (BC) has become a popular material for bone tissue scaffolds due to its ability to improve mechanical performance and structural stability [147]. For various applications, nanocellulose materials have been modified into different forms, including hydrogels, sponges, membranes, fibers, and scaffolds [148,149]. Simplifying the design of cellulose-based composite capsules is crucial in advancing the biomedical field.

#### 3.2.5 Cyclodextrin

Cyclodextrin is a cyclic oligosaccharide composed of glucose subunits linked by α-1,4-glycosidic bonds, forming a macrocyclic ring structure. The cyclodextrin structure, application and bioactive compounds of anti-inflammatory drugs encapsulated in the cyclodextrin molecules (Fig. 11f). They are classified into three types, which are denoted by the different number of glucose units such as α-cyclodextrin for 6 units, β-cyclodextrin for 7 units, and γ-cyclodextrin for 8 units [150]. Moreover, cyclodextrins possess a toroidal structure characterised by a hydrophobic inner cavity and a hydrophilic outer surface. The inner cavity, defined by the primary face and broadened at the secondary face, can be further modulated by the free rotation of primary hydroxyl groups. This unique architecture allows cyclodextrins to form stable inclusion complexes with a wide range of guest molecules, making them highly valuable in biomedical applications such as drug delivery and cell encapsulation. In cell encapsulation, cyclodextrins can be integrated with polymers to create hydrogels that act as scaffolds, offering a biocompatible environment for encapsulated cells [151]. These systems can be readily functionalized with targeting ligands, enabling precise delivery to specific sites, such as cancer cells, while also supporting real-time monitoring of therapeutic outcomes. Collectively, these properties improve drug solubility, encapsulation efficiency, and loading capacity, while simultaneously reducing toxicity.

Moreover, cyclodextrin-based drug delivery systems offer controlled release, improving treatment effectiveness for encapsulated cells by enhancing targeting precision and increasing the solubility of drugs and other bioactive compounds [[152], [153], [154]]. In tissue engineering, cyclodextrins are used in scaffolds to provide a supportive cell growth and differentiation environment. In particular, cyclodextrins are widely recognised for their biocompatibility, making them ideal for various biomedical applications. While also, cyclodextrins hold significant promise in drug delivery and cell encapsulation, with their effective use in biological systems.

### 3.3 Synthetic polymer materials

#### 3.3.1 Polyethylene glycol (PEG)

**Fig. 12:**
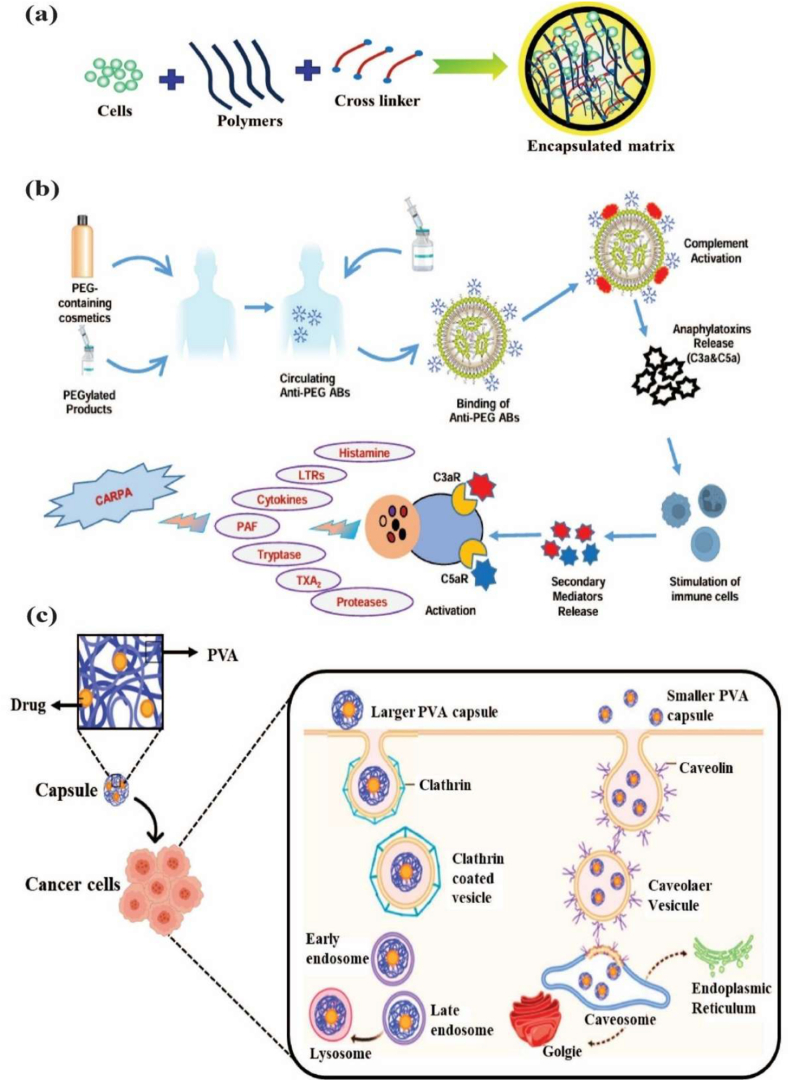
(a) Schematic diagram of cells with polymer-mixed encapsulated matrix. (b) PEG activated circulating system of the human body (Antibodies; C3a and C5a, complement fragments; C3a R and C5a R, complement fragments receptors; LTRs, Leukotrienes; PAF, Platelet activating factor; TXA2, Thromboxane A2; CARPA, complement activation-related pseudoallergy). Reproduced with permission from Ref. [267], Copyright 2022, Elsevier. (c) The PVA capsule interacted with the cancer cells. Reproduced with permission from Ref. [268], Copyright 2021, Elsevier.

Polyethylene glycol (PEG) is a biocompatible and hydrophilic polymer commonly used in biomedical applications due to its versatile properties. Its chemical structure, (CH_2_CH_2_O)_n_, indicates that PEG consists of repeating ethylene glycol units synthesised via anionic polymerization, which also imparts alkaline characteristics. These properties make it ideal for diagnostic and therapeutic uses [155]. Schematic diagram of cells with polymer mixed encapsulated matrix (Fig. 12a). The polyethylene glycol plays a significant role in cell encapsulation by forming semi-permeable membranes that shield living cells from immune cells while permitting the exchange of nutrients and therapeutic agents such as those used in diabetes treatment, cancer therapy, and regenerative medicine [156]. The PEG-activated circulating system of the human body (Antibodies; C3a and C5a, complement fragments; C3a R and C5a R, complement fragments receptors; LTRs, Leukotrienes; PAF, Platelet activating factor; TXA2, Thromboxane A2; CARPA, complement activation-related pseudoallergy) as shown in Fig. 12b. For instance, PEG-encapsulated pancreatic islet cells promise for treating Type 1 diabetes, as the capsules protect the islet cells from immune rejection while allowing them to release insulin. In cancer therapy, PEG encapsulation enables the localized delivery of anti-cancer agents, thereby enhancing treatment efficacy and modulating the immune response. One of the main advantages of PEG is that it can trigger the formation of antibodies, leading to faster clearance from the bloodstream and potentially undermining therapeutic effects. This immune response is mediated through two primary mechanisms: the thymus-dependent pathway, where T cells assist in activating B cells, and the thymus-independent pathway, where PEG nanoparticles directly stimulate B cells to produce antibodies [157,158]. Additionally, PEG-based copolymers such as polylactic acid, poly (amino acid), and poly (ε-caprolactone) can self-assemble into nanoscale drug carriers, advancing the development of novel biomedical treatments. Similarly, provides significant advantages including enhanced stability, prolonged circulation in the bloodstream, and targeted accumulation at tumor sites [159]. Although immune-related challenges remain, polyethylene glycol continues to enhance functional activity at tissue regeneration sites.

#### 3.3.2 Polylactic acid (PLA)

Polylactic acid (PLA) is a semi-crystalline polymer derived from poly-lactide, characterised by aliphatic ester groups that contribute to biodegradability, biocompatibility, and versatility in both clinical and industrial applications [160]. The PLA exists as two stereoisomers: D-lactide and L-lactide. The biocompatibility and biodegradability properties of PLA are suitable for various medical applications, including drug delivery, wound healing, and tissue engineering [161]. This polymer degrades through hydrolysis into lactic acid, which is metabolised by the body. The PLA-based materials have received approval from the Food and Drug Administration (FDA) for several medical uses, including bone fixation devices and controlled drug release systems [162]. The degradation process of PLA is influenced by its crystalline and amorphous phases, with the amorphous regions degrading more rapidly. This characteristic is particularly advantageous for controlled drug delivery. Moreover, PLA has gained significant popularity in 3D printing, especially for biomedical applications, due to its ease of processing and favourable mechanical properties. In tissue engineering, PLA scaffolds can be designed to mimic the native extracellular matrix, providing support for cell attachment, migration, and proliferation. The 3D printing of PLA enables the fabrication of intricate tissue structures, which are essential for creating functional tissues. For instance, PLA is often combined with cells to promote tissue regeneration in applications such as nerve or cartilage repair. The PLA is also used in composite materials combined with biopolymers, inorganic fillers, or nanoparticles, which improve their mechanical strength, bioactivity, and degradation rate. Additionally, PLA-based hydrogels have been explored for drug delivery and wound healing. These hydrogels offer a unique combination of flexibility, water retention, and biocompatibility, making them ideal for wound dressings. They create a protective barrier that shields the wound from external contaminants while maintaining optimal healing conditions, such as moisture retention and oxygen exchange [163]. Furthermore, PLA-based hydrogels support collagen deposition, epithelialization, and angiogenesis, which are the associated critical processes for tissue repair and regeneration. Additionally, surface modification of the biomaterial interface further enhances cellular response and promotes desirable material functionalization.

#### 3.3.3 Polyvinyl alcohol (PVA)

Polyvinyl alcohol (PVA) is a water-soluble thermoplastic polymer derived from polyvinyl acetate. Its linear structure, which contains numerous hydroxyl groups, strengthens the hydrogel matrix by forming both chemical and physical interactions through hydrogen bonding and electrostatic attraction with cross-linking agents. The high mechanical strength, swelling capacity, transparency, biocompatibility, and non-toxicity of PVA make it an ideal material for various biological applications, including antibacterial activity, wound dressing, bone tissue regeneration, and drug delivery [164]. Despite these advantages, PVA exhibits low hydrophilicity, which can limit cell adhesion on its surface. While this property helps prevent unwanted cell growth, it can also hinder cellular interactions. To overcome this limitation, PVA surfaces can be modified to either promote or inhibit cell interactions, depending on the specific requirements of the intended application. In the context of cell encapsulation, PVA offers significant benefits. The PVA capsules interact with cancer cells (Fig. 12c). The ability of PVA allows cells to adhere to the inner surface of the capsule, creating a semipermeable barrier that protects the cells from immune attacks while facilitating the exchange of nutrients and oxygen [[165], [166], [167]]. Moreover, PVA is generally safe for human use and rarely triggers significant immune responses, making it an excellent material for encapsulating living cells. Its thermoplastic nature allows it to undergo structural changes through freeze-thaw cycles, which promote gelation. Ding et al. discussed that PVA/Carboxymethyl chitosan-based hydrogels loaded with taxifolin liposomes promote diabetic wound healing by inhibiting and minimizing inflammatory responses [168]. For instance, encapsulated insulin-producing cells can evade immune system attacks, potentially reducing or eliminating the need for insulin injections and pancreatic cell transplants for diabetes treatment.

#### 3.3.4 Polycaprolactone (PCL)

Polycaprolactone (PCL) is a synthetic polymer derived from caprolactone, distinguished by a unique structure of the ester group and combined with methylene groups. This structural configuration, with remarkable properties such as biodegradability, biocompatibility, and mechanical stability, makes it an excellent candidate for biomedical applications in regenerative medicine, tissue engineering, and controlled drug release, thereby enhancing therapeutic efficacy while minimizing side effects [169]. Nowadays, PCL is widely utilized to synthesise diverse materials, including nanofibers, microspheres, scaffolds, and hydrogels. Each type of material structure offered different advantages. The nanofibers used for drug delivery and tissue scaffolding, nanofibers made from PCL, mimic the extracellular matrix, promoting cell adhesion and growth [170]. The PCL microspheres are highly valued in cancer therapy and other treatments requiring prolonged drug release. Their ability to encapsulate and deliver small molecules, proteins, and nucleic acids in a controlled manner makes them particularly effective [171]. The PCL scaffolds provide structural support for tissue regeneration by offering a framework for cell growth and differentiation. Combining the PCL with hydrogels creates water-absorbing polymer matrices arranged in three-dimensional structures. This combination enhances mechanical strength while enabling the controlled release of therapeutic agents, making it highly effective for wound healing and tissue repair. The ability of PCL to encapsulate both hydrophilic and hydrophobic drugs underscores its versatility [172]. These systems ensure localized and sustained release of therapeutic agents and improve precision in tissue engineering because of their long-term degradation properties of high mechanical strength. It provides a supportive matrix for cell growth and differentiation, crucial for repairing damaged tissues. Inclusive, the polycaprolactone plays a crucial role in regenerative medicine and drug delivery applications.

## 4 Advantages and limitations of natural and synthetic polymers

Natural and synthetic polymers offer distinct advantages and certain limitations for cell encapsulation. Natural polymers, such as collagen, gelatin, silk fibroin, keratin, elastin, sodium alginate, and chitosan, are highly valued for their excellent biocompatibility, biodegradability, and inherent bioactivity, which often promote favourable cell-material interactions while minimizing adverse immune responses [229]. Additionally, their biological origin enables them to support cell adhesion, proliferation, and differentiation, largely due to the presence of intrinsic biological signals that actively guide cellular behaviour and facilitate tissue regeneration. Additionally, appropriately purified natural polymers tend to exhibit low immunogenicity, provoking minimal immune responses. Their hydrophilic character also enhances cell viability by facilitating the diffusion of nutrients and waste products. However, natural polymers have one major drawback is their poor mechanical strength, which restricts their use in load-bearing or structurally demanding environments. Furthermore, batch-to-batch variability, resulting from their biological source, can affect reproducibility and compromise long-term stability. Their unpredictable degradation profiles pose some challenges in various applications where precise degradation timing is very crucial. In contrast, synthetic polymers, including polyethylene glycol, polylactic acid, polyvinyl alcohol, and polycaprolactone, offer greater mechanical strength and controllable degradation rates, making them suitable for applications requiring mechanical integrity and prolonged functional performance. Their chemical tunability and manufacturing consistency also support large-scale production and reproducibility. Nevertheless, synthetic polymers generally lack bioactive cues, often resulting in suboptimal cell responses. Moreover, surface modification is often necessary to enhance cell compatibility and integration with the host tissue [297]. However, these complementary strengths and weaknesses, ultimately leading to the use of composite or hybrid materials that combine both natural and synthetic polymers, have emerged as a promising way to enhance biological performance while maintaining the desired mechanical and chemical properties. Otherwise, the mechanical performance of biomaterials is a critical factor in various biomedical fields. Whether used in tissue scaffolds, implants, or encapsulation systems, these materials must possess mechanical properties that are compatible with the biological environment, supported by various properties such as elasticity, tensile strength, compressive strength, and toughness. For example, materials designed for soft tissue applications must offer flexibility and elasticity to accommodate natural body movements, while those intended for bone replacement require high compressive strength and stiffness to provide adequate support.

### 4.1 Mechanical properties and porosity of natural polymer and synthetic polymer

**Fig. 13:**
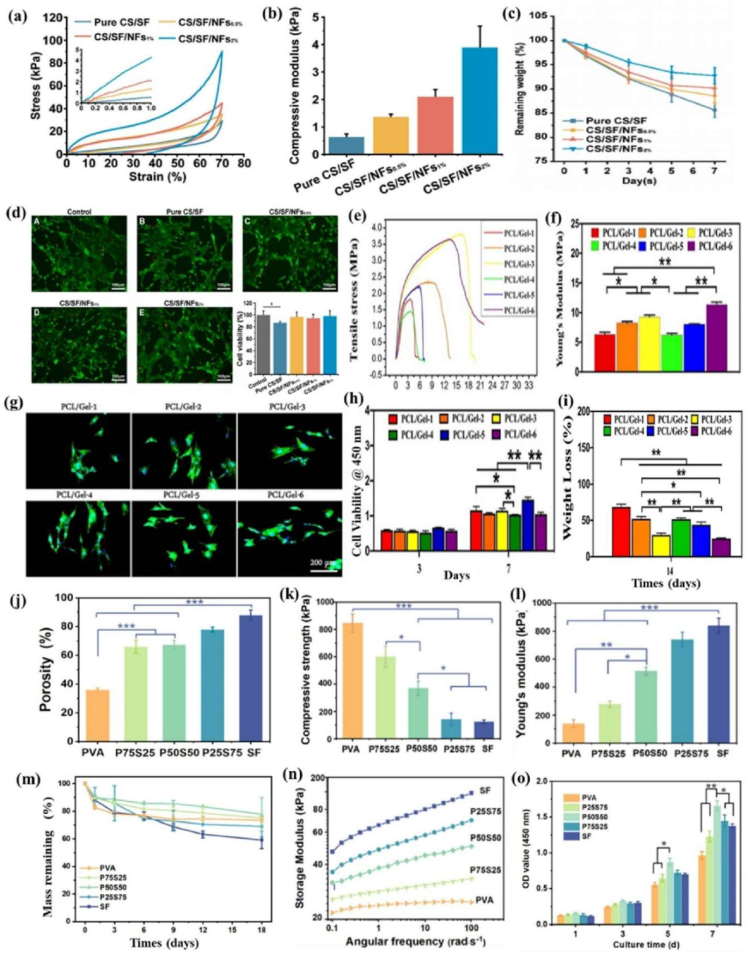
(a) Mechanical strength, (b) compressive modulus (kPa), and (c) degradation profiles of pristine CS/SF and CS/SF/NFs at different concentrations of 0.5 %, 1 %, and 2 %. (d) Biocompatibility assessment: live/dead staining images of control, pristine CS/SF, CS/SF/NFs 0.5 %, CS/SF/NFs 1 %, and CS/SF/NFs 2 %, along with quantitative analysis using the CCK-8 assay. Scale bar 100 μm. Reproduced with permission from Ref. [307], Copyright 2022, Elsevier. (e) Tensile stress (MPa), (f) Young's modulus (MPa), (g) Biocompatibility, (h) Cell viability, and (i) Degradation behaviour of PCL/Gel composite matrices with different ratios (PCL/Gel-1, PCL/Gel-2, PCL/Gel-3, PCL/Gel-4, PCL/Gel-5, and PCL/Gel-6). Microscopic scale bar 200 μm. Reproduced with permission from Ref. [308], Copyright 2021, Elsevier. (j) Porosity, (k) compressive strength (kPa), (l) Young's modulus (kPa), (m) Degradation, (n) Storage modulus (kPa), and (o) Quantitative cell viability analysis of PVA/SF formulations (PVA75:SF25, PVA50:SF50, and PVA25:SF75). Data are expressed as mean ± S.D. (n = 5). Reproduced with permission from Ref. [108], Copyright 2025, Elsevier. Statistical significance was assessed using two-way ANOVA followed by Tukey's post hoc test (∗P < 0.05, ∗∗P < 0.01, ∗∗∗P < 0.001).

The mechanical properties of biomaterials play a crucial role in the cell encapsulation matrix. Beyond structural support, mechanical strength actively influences cellular behaviour through mechanotransduction pathways, regulating key processes such as adhesion, proliferation, differentiation, and survival [33]. Importantly, different cell types display distinct sensitivities to matrix stiffness. For instance, stem cells require softer matrices to maintain pluripotency, but stiffer surroundings can encourage lineage-specific differentiation or provide the mechanical support required for structurally demanding cells such as chondrocytes. However, substrate stiffness can directly regulate stem cell differentiation. Soft materials with a stiffness of ∼0.1–1 kPa promote neurogenic differentiation, moderate stiffness of ∼10 kPa favours myogenic differentiation, and higher stiffness above 30 kPa supports osteogenic differentiation [210]. These findings highlight the need to carefully tailor biomaterial mechanical qualities to ensure construct stability as well as functional outcomes for encapsulated cells. For example, the mechanical strength of pristine CS/SF and CS/SF/NFs was evaluated at different concentrations. The mechanical strength and compressive modulus of the pristine CS/SF exhibited a compressive strength of 29.5 ± 1.3 kPa. The incorporation of different concentrations of nanofibers (NFs), added to the pristine CS/SF matrix, which concentration increases the mechanical and modulus strength as 31.8 ± 3.0 kPa (0.5 % NFs), 44.8 ± 2.7 kPa (1 % NFs), and 92.4 ± 3.2 kPa (2 % NFs) at 70 % strain (Fig. 13a and b). This improvement can be attributed to the reinforcing effect of NFs, which distribute stress more efficiently within the matrix and restrict deformation, thereby improving both stress resistance and modulus. The degradation study of pristine CS/SF and CS/SF/NFs at different concentrations (Fig. 13c). After 7 days in PBS, the remaining weights of the nanofiber increased proportionally from 85.7 % to 92.8 %. This study indicates that NFs not only reinforced mechanical properties but also delayed degradation by reducing hydrolytic susceptibility [307]. The biocompatibility tested of control, pristine CS/SF, CS/SF/NFs 0.5 %, CS/SF/NFs 1 %, and CS/SF/NFs 2 % was performed using live/dead staining revealed no significant differences between scaffold groups and the control group (Fig. 13d), suggesting that incorporation of NFs did not produce cytotoxic effects. Quantitative CCK-8 analysis confirmed this observation, with all groups maintaining >80 % viability relative to the control, although a minor reduction was noted in the 0.5 % NF group. The tensile stress and Young's modulus of PCL/Gel composites revealed a reduction in mechanical performance at higher PCL/Gel ratios, with PCL/Gel-6 exhibiting the lowest values (Fig. 13e and f). The biocompatibility of PCL/Gel-1 to PCL/Gel-6, evaluated using MC3T3-E1 cells, showed comparable cell viability after 3 days, supported by the presence of protruding filamentous pseudopodia, indicating strong cell-scaffold interactions (Fig. 13g and h). The degradation study of PCL/Gel-1 to PCL/Gel-6 composite matrices (Fig. 13i) revealed that PCL/Gel-6 exhibited the lowest degradation rate (29 %), suggesting that slower degradation aligns with tissue formation rates, allowing cells sufficient time to produce their own extracellular matrix before the scaffold disappears [308]. In the PVA/SF system with varying concentrations (75:25, 50:50, and 25:75), increasing SF content markedly enhanced the porosity from 35 % to 87 % as denoted in (Fig. 13j), emphasising the role of SF in tuning the pore network to support nutrient and oxygen transport. The PVA based mechanical testing further revealed that compressive strength increased with PVA content, reflecting the crystalline reinforcement effect of the PVA ratio (Fig. 13k and l). However, degradation analysis after 18 days of PBS immersion demonstrated a mass loss of around 40 %, primarily due to the hydrolysis of SF peptide bonds (Fig. 13m). The dynamic storage modulus of the PVA/SF composite matrix was lower than that of pure SF, likely due to PVA-induced amorphous expansion, which weakened pore water retention (Fig. 13n). Quantitative analysis of cell viability further confirmed that the SF hydrogel promoted significantly higher proliferation compared to pure PVA (Fig. 13o). This demonstrates the synergistic role of SF in enhancing both porosity and biological performance, thereby supporting the cell encapsulation matrix [108]. The mechanical properties of natural and synthetic polymer material composite matrix [298] are described (Table 3).

**Table 3:** Represented the mechanical properties of natural and synthetic polymer material composite matrix.

Natural polymer	Synthetic polymer	Descriptions (Weight ratio)	Tensile strength (MPa)	Elasticity modulus (MPa)	Elongation break
Silk fibroin	PLA	PLA/SF (75:25)	20.51 ± 3.97	0.47 ± 0.07	26.22 ± 10.64
Silk fibroin	PLA	PLA/SF (50:50)	16.49 ± 8.71	1.05 ± 0.43	17.51 ± 3.89
Silk fibroin	PVA	PVA/SF (1:1)	11.2 ± 1.9	293 ± 64	5.8 ± 0.07
Silk fibroin	P (LLA)	P (LLA)/SF (1:1)	25.78 ± 5.25	–	320.56 ± 41.68
Collagen Type 1	PCL	PCL/collagen	4.0 ± 0.4	2.7 ± 1.2	140 ± 13
Collagen Type I	P (LLA)	P (LLA)/collagen (1:1)	6.27 ± 1.38	43.99 ± 4.04	–
Gelatin	PCL	PCL/gelatin (70:30)	12 ± 1.8	180 ± 28	280 ± 62
Sodium Alginate	PEO	PEO/SA (9:1)	20.9 ± 1.6	–	–
Keratin	PCL	PCL/Keratin (90/10)	–	10.00 ± 2.05	3.00 ± 1.25
Chitosan	PCL	PCL/CS	1.78 ± 0.25	12.35 ± 1.38	31.13 ± 7.5
Elastin	PLGA	PLGA/Elastin	0.37 ± 0.83	–	2.96 ± 1.15
Sodium Alginate	PLA	PLA/SA (10:90)	4.25 ± 0.39	116.56 ± 70.2	12.10 ± 0.35

**Fig. 14:**
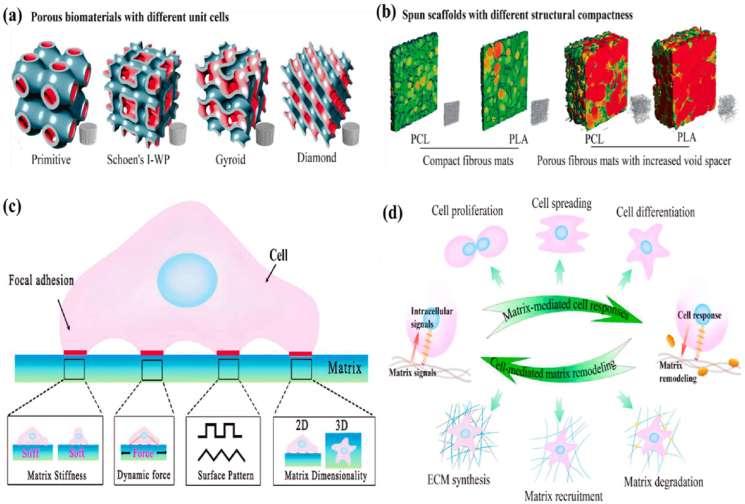
(a) Schematic diagram of biomaterials with different cellular units. Reproduced from Ref. [58], Copyright 2022, Elsevier. (b) Fibrous mats with varying degrees of structural compactness. Reproduced from Ref. [58], Copyright 2022, Elsevier. (c) Cell and biomaterial interactions of mechanical signals, including stiffness, external dynamic forces, surface patterns, and matrix dimensionality. Reproduced from Ref. [58], Copyright 2022 (d) Schematic diagram of biomaterial matrix-mediated cellular responses and cell-mediated matrix remodelling. Reproduced from Ref. [58], Copyright 2022, Elsevier.

Pore size and porosity are crucial functions of immunomodulation and supporting tissue regeneration. These structural features define the local microenvironment, directly influencing cellular adhesion, proliferation, migration, and differentiation. Low porosity typically restricts mass transport and cellular infiltration, supporting only limited adhesion and growth, whereas higher porosity enhances nutrient exchange and cell proliferation [48]. Therefore, precise regulation of porosity is balanced with mechanical integrity and biological activity. The authors Rnjak-Kovacina et al. discussed elastin scaffolds with higher porosity (34.4 ± 1.3 %) that significantly enhanced cell migration and infiltration, whereas those with lower porosity (14.5 ± 0.8 %) failed to produce comparable effects [49]. Schematic diagram of biomaterials with different cellular units (Fig. 14a). Fibrous mats with varying degrees of structural compactness demonstrate the influence of cell behaviour (Fig. 14b). However, these fibrous mats provide enhanced mechanical support but may limit cell infiltration, nutrient diffusion, and interaction with the extracellular environment. The cells and biomaterial matrix interaction of mechanical cues, including stiffness, external dynamic forces, surface patterns, and matrix dimensionality (Fig. 14c). These interactions regulate key cellular processes, including adhesion, migration, proliferation, and differentiation. Schematic diagram of biomaterial matrix-mediated cellular responses and cell-mediated matrix remodelling, as shown in Fig. 14d. A well-designed matrix promotes efficient integrin binding and cytoskeletal organization, which in turn enhances extracellular matrix deposition and tissue integration. A highly interconnected porous network enhances mass transport and waste removal, thereby improving cell viability, functionality, and host tissue integration. The larger pore sizes and higher porosity are particularly beneficial for metabolically active cells such as pancreatic islets, hepatocytes, and cardiomyocytes. In contrast, lower metabolic requirements are better supported by smaller pore sizes, which simultaneously enhance mechanical strength and structural durability. These parameters are interconnectivity in nutrient diffusion, metabolite clearance, and the long-term functional activity of encapsulated cells. Beyond metabolic support, porosity also regulates key biological processes, including cell infiltration, migration, angiogenesis, and extracellular matrix, that collectively drive host tissue integration and functional remodelling. Thus, the design of cell-encapsulating biomaterials requires a finely tuned balance between pore size, porosity, and mechanical stability. Together, these features underscore the pivotal role of porosity in advancing tissue engineering applications.

## 5 Cell-material interaction

The integration of natural and synthetic polymers offers a synergetic approach to optimizing the performance of cell capsules by balancing mechanical strength with biological activity. The natural polymers are provided a biomimetic environment conducive to cell survival and function, while synthetic polymers contribute to enhanced structural stability and tunable degradation rates [293]. The combination of these materials significantly impacts key parameters such as pore size and mechanical strength, which must be precisely controlled to facilitate the diffusion of oxygen, nutrients, and therapeutic agents while effectively excluding immune cells and large antibodies. Moreover, the microfluidic method of capsule preparation plays a pivotal role in determining capsule morphology, mechanical integrity, and encapsulation efficiency. Among these, microfluidic techniques have emerged as powerful approaches allowing for high-throughput generation of monodisperse capsules with finely controlled size and architecture. This approach also supports the creation of sophisticated core-shell structures and offers spatial control over cell distribution, improving cell viability and functional integration [294]. Furthermore, 3D bioprinting, a transformative technology used in capsule preparation, enables spatial patterning and the layer-by-layer assembly of cell-laden biomaterials, facilitating the construction of complex, heterogeneous capsule architectures that more accurately replicate native tissue environments. However, their encapsulation must be precisely tailored to either maintain stemness or direct differentiation toward specific lineages, depending on the therapeutic agent. Across all cell types, standardised cell preparation protocols are essential for preserving membrane integrity, metabolic activity, and phenotypic stability. Furthermore, the interaction between cells and materials during the encapsulation process must be carried out under sterile and tightly controlled conditions, as it plays a crucial role in enhancing functionality and supporting the clinical translation of therapeutic outcomes.

The biological performance of cell encapsulation systems is strongly influenced by the different types of cells used in their specific cellular states. The cell deviation throughout multiple stages, including proliferation, quiescence, differentiation, and senescence, results in each stage having different superior properties of the encapsulated matrix. Proliferative cells maintain high metabolic activity to support tissue regeneration and rapid integration of host environments. In contrast, quiescent cells exhibit reduced metabolic demands and temporarily exit the cell cycle, which can confer resistance to stress but may limit regenerative capacity. Cellular senescence is a multidimensional biological process that induces different cellular morphology, structure, and function. The senescence-associated secretory phenotype (SASP), which aids in tissue regeneration and immunological function, is also characterised by the release of pro-inflammatory mediators and irreversible cell-cycle arrest. Furthermore, transforming growth factor-β (TGF-β) is a crucial cytokine that plays a pivotal role in various biological processes, including migration, differentiation, and cell proliferation. However, encapsulating senescent or pre-senescent cells within biomaterial matrices can produce different biological responses than proliferative cells, impacting the regeneration potential of cell encapsulation therapies. This property has been used to deliver precise drugs, target therapeutic interventions, and modulate senescence mechanisms. Notably, senescent cells exhibit a unique antigenic profile characterised by the expression of neoantigens and aberrant autoantigens not typically present in normal cells. In addition to structural and immunological changes, senescent cells exhibit heightened metabolic activity, characterised by elevated oxygen consumption and dysregulated flux through glycolysis, lipid catabolism, and mitochondrial oxidative phosphorylation, which enables them to sustain ATP production [220].

### 5.1 Injectable hydrogel for cell matrix interaction

**Fig. 15:**
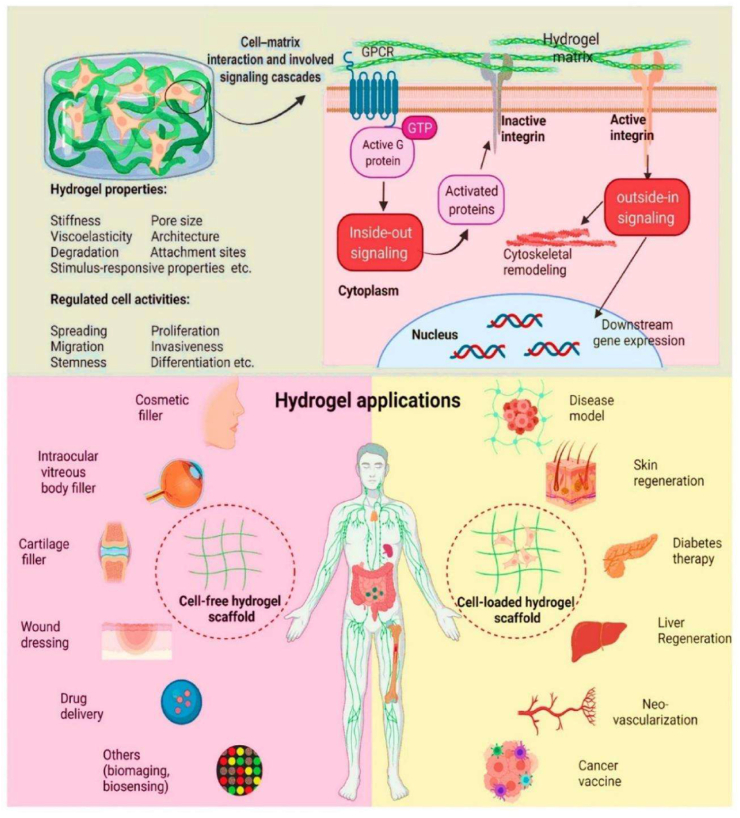
Schematic diagram of cell-matrix interaction in the biomedical applications of both cell-free and cell-loaded hydrogel. Reproduced from Ref. [250], Copyright 2021 Springer Nature.

Injectable hydrogels have emerged as a platform in translational medicine for regulating cell functional activity in various ways, including tissue engineering, drug delivery, and wound healing [87]. Typically, hydrogel materials are particularly well-suited for cell encapsulation due to their high water content, biocompatibility, and ability to simulate the extracellular matrix, which is essential for supporting cellular activities [90,91]. These materials must provide structural support while creating an environment conducive to the survival, differentiation, and function of the embedded cells. Additionally, the hydrogel material needs to be engineered to achieve optimal stiffness, porosity, and degradability, thereby facilitating cellular attachment and growth while maintaining structural integrity [92]. It can mimic the native extracellular matrix, offering a 3D structure that supports essential cellular activities such as migration, adhesion, proliferation, and differentiation, making it a valuable application in cell therapy, tissue regeneration, and localized drug delivery [88]. Biological factors increasingly support the roles of biocompatibility, mechanical strength, and tunable degradation rates. These unique properties make hydrogels especially suitable for creating hydrogel capsules in cell encapsulation, providing a supportive environment for cellular growth, nutrient diffusion, and cell-matrix interactions. The mechanical properties of hydrogels can be precisely tuned to guide cell fate: softer hydrogels tend to promote stem cell differentiation into neural or adipose lineages. In comparison, stiffer hydrogels encourage differentiation into osteogenic or chondrogenic lineages. This ability to control cell behaviour through mechanical cues provides a powerful tool for tissue engineering and regenerative medicine. In addition, incorporating biomolecules such as peptides, antibodies, or cytokines into hydrogels can release bioactive substances that enhance the regulation of cell adhesion, migration, and differentiation [93]. This enables precise modulation of cell growth, tissue formation, and immune responses. The schematic diagram illustrates cell-matrix interactions in both cell-free and cell-loaded hydrogels for biomedical applications (Fig. 15). The injectability and flexibility of hydrogels are key features that make them particularly suitable for immune-related therapies. Since injectable hydrogels can accommodate both cell-free and cell-loaded systems, they enable the creation of dynamic environments that support tissue regeneration, functional repair [95], and improved therapeutic outcomes.

Looking ahead, integrating biological cells with synthetic materials will likely lead to the development of bio-hybrid systems with applications in biotechnology, bioelectronics, and advanced robotics. Although still in the early stages, the potential applications for living cell robotics, bio-hybrid systems, and advanced regenerative therapies are promising. Furthermore, hydrogel capsules can act as protective barriers, shielding encapsulated cells from hostile environments, such as immune rejection in allogeneic or xenogeneic transplantation [89]. Additionally, the hydrogel must maintain structural integrity in physiological environments while promoting cell viability and integration with host tissue. In supporting endogenous repair, injectable hydrogels can leverage the body's innate regenerative capacity by creating a bioactive microenvironment that facilitates the infiltration of host cells and blood vessels. Moreover, these injectable hydrogel-based cell capsules offer numerous advantages for a wide range of biomedical applications.

### 5.2 Endoe cell new function of bio-hybrid robotic capsule

Endoe cells or living-cell-based robotic systems represent a new frontier in biological fields. In the context of robotics, these bio-hybrid systems can serve as soft actuators, enabling delicate and flexible movements. These robots hold immense potential in medical applications, such as minimally invasive surgeries, where they can autonomously navigate the body, identify specific tissues or structures, and perform repairs or deliver targeted therapies. Additionally, the surface modification of bioactive polymers could support immune responses and promote cell proliferation in response to environmental signals, making them ideal for biomedical applications. However, living cells combined with synthetic materials in bio-hybrid systems have the potential to unlock new therapeutic possibilities and offer innovative solutions across a wide range of biological fields. The cell capsules are defined by their cellular constituents, which play a pivotal role in determining both the therapeutic and clinical applications. This section examines the diverse cell types used in encapsulation, their sources and preparation methods, and the critical cell-material interactions that ultimately influence therapeutic efficacy. Otherwise, endoe cell bio-hybrid systems can perform a wide range of functions by integrating living cells with a synthetic material composite matrix, aiming to mimic the cellular functionality and responsiveness of living organisms while harnessing the precision and versatility of artificial materials. Currently, various therapeutic applications require the use of specific cell types, each with distinct functional properties. For instance, pancreatic islets commonly employed in the treatment of type 1 diabetes require encapsulation systems that support high oxygen permeability and precise glucose responsiveness. The mesenchymal stem cells (MSCs), known for their multipotency and robust secretion of trophic and immunomodulatory factors, are utilized in regenerative medicine [289]. In advanced therapeutic platforms, genetically engineered cells require encapsulation systems that can sustain the secretion of therapeutic agent, contain genetic material, and effectively evade immune surveillance. Similarly, induced pluripotent stem cells (iPSCs) hold great promise for personalised medicine due to their autologous origin; however, their application necessitates tightly controlled encapsulation strategies to prevent uncontrolled differentiation and teratoma formation. The development of bio-hybrid robotic smart hydrogels represents a remarkable intersection of biomaterials, robotics, and smart systems, with the potential to revolutionise fields such as biotechnology and environmental monitoring. In biotechnology, these systems can be used to monitor and modulate biological processes at the cellular level, enabling innovative diagnostic and treatment. In environmental monitoring, bio-hybrid robots could detect pollutants, pathogens, or changes in environmental conditions, offering a more adaptive and flexible approach than traditional sensors. One of the primary challenges is integrating living cells with synthetic materials to ensure seamless functionality. These challenges require significant advancements in material science, particularly in the development of hydrogel capsules and other substrates that can support the long-term survival and performance of embedded cells.

### 5.3 Artificial intelligence-based prediction of cell-material interactions

Artificial intelligence (AI) has emerged as a transformative tool in predicting and analysing cell-material interactions is a critical aspect in tissue engineering, regenerative medicine, and biomaterials design. Traditional approaches to understanding these interactions are often time-consuming and limited by the complexity of biological systems. For instance, AI used to elucidate how nanoscale surface features influence stem cell differentiation pathways by correlating them with gene expression profiles. Furthermore, generative adversarial networks and variational autoencoders are facilitating the design of innovative biomaterial structures with desired biological functionalities. Despite these advancements, AI-driven predictions face significant challenges, including the need for high-quality, standardised datasets, model interpretability, and experimental validation [299]. Moreover, AI can be used to predict and optimize interactions between cell capsules and biomaterials, especially in tissue engineering, regenerative medicine, and drug delivery systems. Machine learning (ML) and artificial intelligence are being increasingly integrated into materials science and biomedical engineering to accelerate the development of advanced biomaterials. In the context of cell-material interactions, AI offers significant advantages by enabling predictive modelling that can replace or augment traditional trial-and-error approaches. Furthermore, ML-assisted material formulation leverages large datasets and computational algorithms to identify optimal material compositions and properties that promote desirable cellular responses. For example, ML models can predict how variations in cross-linking density or surface topography affect cell adhesion, proliferation, and differentiation [300]. This data-driven approach enables researchers to rapidly screen and optimize materials for specific applications, such as tissue scaffolds and drug delivery systems. For instance, predictive models can estimate how capsule stiffness, porosity, or influence cell viability, immune modulation, and tissue integration. These insights are essential for developing cell encapsulation systems that maintain long-term functionality and biocompatibility. The AI predictions are especially useful for understanding intricate, nonlinear relationships between material parameters and design, as they suggest completely new material structures with specific biological functions [301]. Despite their potential, the implementation of artificial intelligence used cell encapsulation faced several challenges. One major limitation is the scarcity of large, high-quality, and standardised datasets required to train robust models. Additionally, ensuring the interpretability and generalizability of AI predictions remains a significant concern, especially in complex biological environments. Looking ahead, AI-assisted approaches are expected to play an increasingly central role in the personalised design of biomaterials, enabling the development of smart, adaptive materials tailored to individual patient needs. While continued advancements in data acquisition, modelling techniques, and experimental validation make machine learning a promising field, it has the potential to transform the landscape of cell-material interactions and significantly accelerate the clinical translation of biomedical applications [302]. These approaches are also being explored to design an optimised biomaterial capsule formation. The predictive capabilities not only accelerate the experimental process but also play a crucial role in the personalised design of the cell encapsulation structure.

### 5.4 Long-term biocompatibility

The long-term biocompatibility of encapsulated systems remains a crucial determinant of their clinical application. Biocompatibility requires that the capsule perform its intended function without provoking undesirable local or systemic effects in the host over extended periods. Several factors influence this property, including the choice of encapsulating material, the nature of degradation products, host immune responses, and the mechanical stability of the construct under physiological conditions [251]. Both natural and synthetic polymers have been extensively explored for encapsulation, owing to their relatively low immunogenicity and tunable degradation profiles. Despite these advances, achieving long-term biocompatibility remains one of the most challenging aspects in cell encapsulation technologies. Addressing this challenge requires, from a materials perspective, the design of next-generation biomaterials with outstanding mechanical properties, controlled degradability, and bio-inert surfaces, which are essential for maintaining capsule integrity and reducing adverse host responses. At the cellular level, recreating a supportive microenvironment through adequate nutrient delivery, oxygenation, and incorporation of bioactive compounds ensures both the survival and sustained functionality of encapsulated cells. Taken together, this integrative approach not only enhances the likelihood of clinical translation but also paves the way for robust, adaptive encapsulation systems that can maintain therapeutic efficacy over extended periods. Typically, encapsulated cells are semi-permeable polymeric membranes, which protect them from the host immune system while simultaneously allowing the exchange of nutrients, metabolites, and therapeutic molecules for superior performance in biomedical applications.

### 5.5 Scalability

The scalability of capsule production plays a pivotal role in advancing cell encapsulation technologies from laboratory research to clinical translation for industrial applications. To meet specific demands, it should be capable of producing large quantities of capsules with consistent size, shape, and functionality to fulfil the unique requirements of clinical therapies. For this purpose, several fabrication techniques have been investigated. However, microfluidics and 3D bioprinting have emerged as particularly promising because of their capacity to create highly monodisperse capsules with precisely controlled structures [269]. These approaches allow for fine-tuning of capsule geometry, porosity, and composition, which are crucial for maintaining cell viability and ensuring consistent therapeutic outcomes. Despite these advances, scaling up these technologies to achieve a high-volume ratio. Laboratory-scale methods, while offering excellent control over capsule morphology, encapsulation efficiency, and material properties, often lack the throughput necessary to meet clinical-scale requirements. Moreover, achieving cost-effectiveness and regulatory compliance further complicates the transition from small-scale research platforms to industrial-scale manufacturing. Beyond scalability, immune rejection represents another major barrier to the success of encapsulation strategies. Producing encapsulated cells in sufficient quantities for clinical use requires reproducible, standardised protocols that preserve cell viability, functionality, and uniform encapsulation. At the same time, insufficient immunoisolation and the intrinsic immunogenicity of certain biomaterials can trigger local inflammatory responses, leading to fibrosis, impaired diffusion, and ultimately reduced survival and functionality of transplanted cells. Therefore, in evaluating the long-term viability of encapsulated cell therapies, it's essential to develop encapsulation platforms that are reliable, reproducible, and clinically translatable for demand in large-scale therapeutic applications.

### 5.6 Bottlenecks of large-scale production

The large-scale production of microfluidic chips is crucial due to their significant potential in the biomedical industry. In conventional microfluidic chip fabrication through soft lithography, particularly using polydimethylsiloxane (PDMS), has become the standard for prototyping due to its low cost, optical transparency, biocompatibility, and ease of fabrication [322]. However, PDMS devices exhibit high gas permeability and have poor bonding with various substrates, which further limits their durability and usefulness in long-term biological assays, presenting some challenges, including mass production and limited mechanical stability. To overcome these issues, alternative industrial manufacturing methods have been explored. Injection moulding and roll-to-roll processing have become popular because they offer higher reproducibility and utilise thermoplastics with better mechanical strength than PDMS. Nonetheless, these methods are restricted by high costs, limited design flexibility, and material compatibility issues. In another emerging technology, high-speed bioprinting offers a novel approach to creating custom designs and simplifies the fabrication of various structures. To guarantee reproducibility and dependability for biological applications, broad acceptance still requires strong standardisation, significant cost reduction, and full regulatory approval. To facilitate extrusion and quickly solidify, preserving shape integrity after printing, bioinks must carefully balance their rheological characteristics. High-viscosity bioinks improve structural integrity and resolution but often compromise cell viability due to shear stress. Conversely, low-viscosity formulations preserve cell health but may compromise mechanical stability. Recently, high-speed bioprinting has emerged as a transformative development in this field. It enables the fabrication of complex, multicellular structures within minutes, rather than days, thereby accelerating clinical translation and facilitating real-time, intraoperative applications such as tissue repair [323]. The combination of high-resolution printing platforms with adjustable bioinks supports the creation of multi-material, biologically relevant architectures that mimic native tissue microenvironments. Therefore, these structures can be utilized in various fields, including tumor modelling, vascular regeneration, skin repair, and the development of patient-derived tissue models for personalised cancer therapy. Together, advances in microfluidic chips and high-speed bioprinting are overcoming material design and manufacturing challenges while broadening the scope of biomedical applications.

## 6 Cell encapsulated biomaterials for cancer therapy

**Fig. 16:**
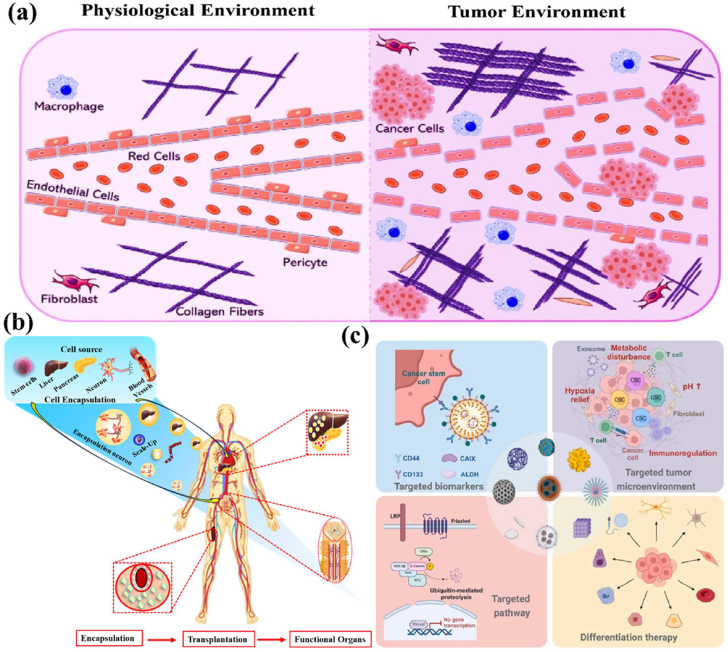
(a) Endothelial cell interactions of differentiation between physiological environment (normal cell line) and tumor environments (cancer cell line). Reproduced from Ref. [271], Copyright 2021 Frontiers in Pharmacology. (b) Cell encapsulation of functional organ implantation into the body. (c) Schematic diagram of targeted cancer stem cell (CSC) therapies in biomedical engineering, highlighting strategies such as biomarker targeting, tumor microenvironment modulation, signalling pathway inhibition, and differentiation therapy. Reproduced with permission from Ref. [227] Copyright 2025, Elsevier.

Cell encapsulation technology represents an innovative approach to cancer therapy, wherein living cells such as stem cells or immune cells are enclosed within protective capsules. This strategy has broad applications, including the treatment of diabetes, neurological disorders, liver diseases, tissue regeneration, chronic disease management, and targeted drug delivery [188]. Biomaterial-based encapsulation enhances immune responses while minimizing phenotypic changes in transplanted cells, thus preserving their functionality within the host. The encapsulating biomaterials create an optimal physical and biochemical environment that supports cellular performance and improves therapeutic efficacy. Moreover, this system allows for the controlled and sustained release of therapeutic proteins, insulin, growth factors, or hormones over extended periods [189]. Encapsulation also protects sensitive therapeutic cells from harsh post-transplantation conditions, such as oxidative stress, inflammation, or mechanical trauma, thereby enhancing their survival and viability. For example, in patients with Type 1 diabetes, this approach holds the potential to reduce or even eliminate the need for regular insulin injections. In cancer therapy, cell encapsulation involves enclosing therapeutic cell aggregates, such as immune or stem cells, within a polymeric capsule for targeted tumor treatment. This protective encapsulation not only shields therapeutic cells from the host immune system but also from the hostile tumor microenvironment. Immune cell encapsulation, involving T cells, NK cells, or dendritic cells, primarily aims at enhancing immunomodulation and enzymatic cytotoxicity to destroy tumor cells. In contrast, encapsulating stem cells, particularly mesenchymal stem cells, aids in tissue repair, modulates the tumor microenvironment, and delivers therapeutic agents. These two strategies, which synergistically encapsulate first immune cells followed by stem cell capsules, enable the precise targeting of tumor destruction. The second stem cell capsules promote tissue repair, reduce inflammation, and enhance immune responses via paracrine signalling. This combination enhances immune functionality and improves therapeutic outcomes. For instance, encapsulated immune cells, such as T cells or dendritic cells, can target and kill tumor cells more effectively. The encapsulating materials can be engineered to release therapeutic factors in response to specific stimuli within the tumor microenvironment [196]. Endothelial cell interactions and differentiation were compared between the physiological environment and tumor environment (Fig. 16a). A schematic illustrates cell encapsulation in various organs for implantation (Fig. 16b). Another schematic provides an overview of targeted cancer stem cell (CSC) therapies in biomedical engineering, highlighting strategies such as biomarker targeting, tumor microenvironment modulation, signalling pathway inhibition, and differentiation therapy (Fig. 16c). In contrast, cell-based drug delivery platforms offer several advantages, including prolonged circulation time, enhanced targeting capabilities, and the ability to overcome physiological barriers. Different types of cell encapsulation in translational medicine (Table 4).

**Table 4:** Different types of cell encapsulation in translational medicine.

Cell Type	Purpose of Encapsulation	Effect on Cell Function	Applications	Refs
T Cells	Enhancing immune therapies, protecting from immune rejection	Maintains viability and function; controlled activation	Cancer immunotherapy, autoimmune disease treatment	[175]
Natural Killer Cells	Enhancing cytotoxicity, improving persistence *in vivo*	Maintains cytotoxic activity, prevents overactivation	Cancer therapy, antiviral therapies	[177]
B Cells	Prolonging antibody secretion, modulating immune responses	Sustained antibody production, controlled immune modulation	Autoimmune therapy, vaccine development	[179]
Vascular Endothelial Cells	Supporting angiogenesis, improving vascularization	Promotes endothelial function, supports vessel formation	Tissue engineering, ischemic therapy	[184]
Stem Cells (MSCs, iPSC)	Enhancing cell survival, directing differentiation	Controlled differentiation, improved viability	Regenerative medicine, tissue engineering	[186]
Macrophages	Modulating inflammatory response, promoting tissue repair	Maintains polarization (M1/M2) and phagocytic function	Wound healing, anti-inflammatory therapies	[272]
Dendritic Cells	Enhancing antigen presentation, controlled immune stimulation	Prolonged antigen presentation, regulated immune activation	Cancer vaccines, immune modulation	[272]

Immunomodulation is the process of regulating the immune cell activity. A key approach in this context is encapsulation, which acts as a protective barrier that shields therapeutic cells from immune rejection while maintaining their survival and functional integrity. In addition, encapsulated immunomodulatory agents such as cytokines can stimulate immune activation and destroy tumor cells [320]. Otherwise, the extracellular matrix (ECM) is essential for tissue regeneration and repair in immunomodulation and is structurally supported by a well-organised network of proteins, glycoproteins, and polysaccharides. Additionally, this type of biomaterial capsule composition, which mimics the characteristics of the extracellular matrix, can promote cell adhesion and differentiation. Importantly, the ECM also regulates essential cellular processes required for tissue growth and homeostasis. Following implantation, the initial immune response is dominated by innate immune cells such as neutrophils, macrophages, and dendritic cells, whose activity strongly influences how implanted materials integrate with host tissues. The immunological perspective, encapsulating biomaterials, acts as a dynamic interface between donor cells and the host immune system. For example, hydrogels enriched with bioactive ligands can suppress pro-inflammatory signalling while promoting tolerogenic phenotypes in macrophages and regulatory T cells, thereby reducing graft rejection and enabling long-term therapeutic benefit. Together, these dual functions of immunomodulation and ECM remodelling-based encapsulation system are powerful approaches for helping effective immunoregulatory activity. These findings establish new benchmarks for designing next-generation therapeutic strategies against fibrotic diseases [321]. Moreover, encapsulation systems hold strong potential for achieving synergistic immune regulation and regenerative outcomes, paving the way for translational applications in cancer therapy, autoimmune diseases, and tissue regeneration.

### 6.1 Cell types

#### 6.1.1 Immune-cell (T cells, B cells, natural killer cells, macrophages, and dendritic cells)

**Fig. 17:**
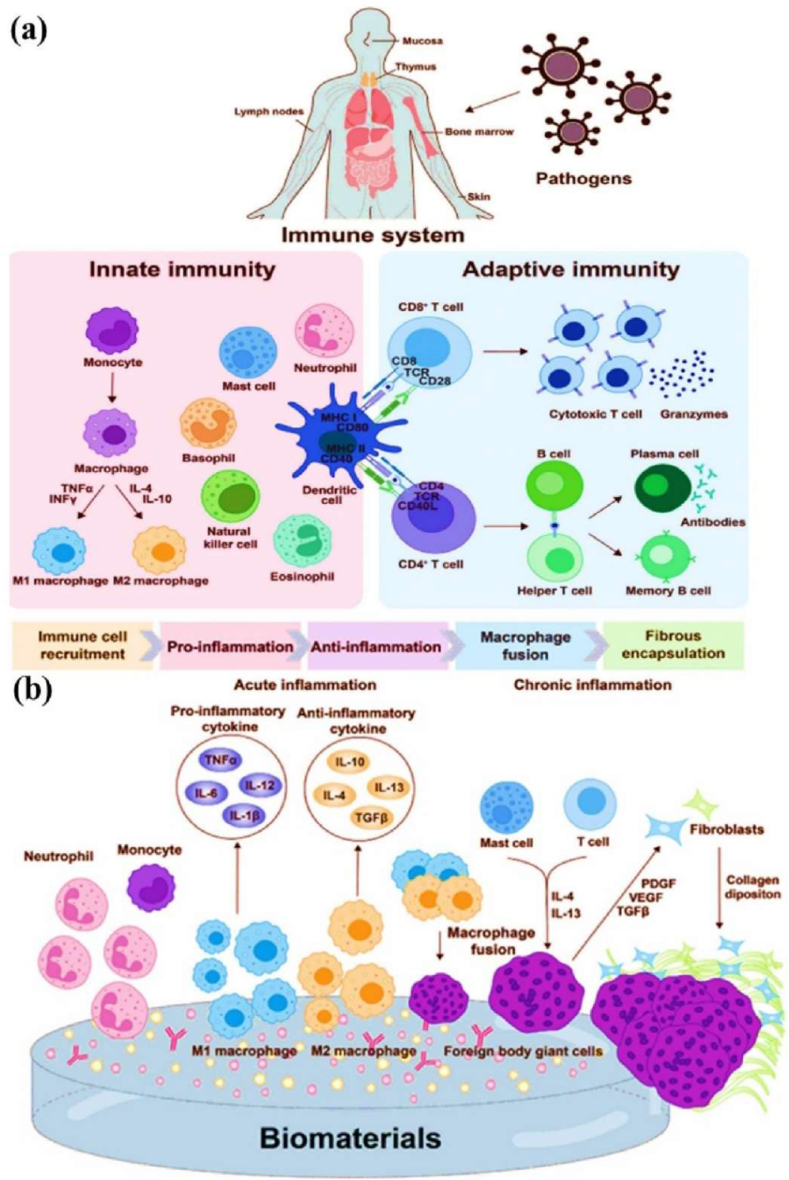
(a) The immune system consists of various immune cells that play distinct roles in immune responses. Innate immune responses are primarily mediated by macrophages, dendritic cells, neutrophils, and natural killer (NK) cells (left). In contrast, adaptive immune responses involve T cells and B cells (right). Macrophages exhibit diverse functional phenotypes, with M1 macrophages activated by IL-4 and IL-10, while TNF-*α* and IFN-*γ* activate M2 macrophages. Reprinted with permission from Ref. [272], Copyright 2025, Wiley-VCH. (b) Biomaterial-based immune cells interacted with acute and chronic inflammation processes. Reprinted with permission from Ref. [272], Copyright 2025, Wiley-VCH.

Immune cells, also referred to as white blood cells or leukocytes, are specialized cells that serve to safeguard the body against infections, foreign substances, and abnormal cells, including malignancies. The immune system consists of various immune cells that each have unique roles in immune responses (Fig. 17a). The innate immune responses are primarily mediated by macrophages, dendritic cells, neutrophils, and natural killer (NK) cells. In contrast, adaptive immune responses involve T cells and B cells. Macrophages exhibit diverse functional phenotypes, with M1 macrophages activated by IL-4 and IL-10, while TNF-*α* and IFN-*γ* activate M2 macrophages [173,174]. However, cancer cells often employ strategies to evade immune detection by creating an immunosuppressive tumor microenvironment (TME), which reduces the effectiveness of immune responses. Compared to other cell types, immune cells have distinct advantages, including high specificity, the ability to generate long-lasting immunological memory, and effectiveness against both primary and metastatic cancer. Additionally, due to their unique immunological properties, immune cells play a crucial role in modulating the TME. Their exosomes can influence the behaviour of cancer cells and the surrounding stroma, either enhancing anti-tumor immunity or contributing to immune suppression. T cells play a vital role in triggering immune responses against autoimmune diseases, infections, and cancers. Interestingly, T cells can either enhance antitumor immunity by activating other immune cells or support tumor growth through immunosuppression [175]. One of the most common tumor-infiltrating lymphocytes, T cells play a crucial role in initiating immune responses. Specifically, exosomes from cytotoxic T lymphocytes display surface molecules such as CD3, CD8, and antigen-specific T-cell receptors, which help deliver targeted cytotoxic signals to cancer cells. Chimeric antigen receptor (CAR) T cells have emerged as an innovative and highly effective method for cancer treatment due to their precise targeting capabilities. For example, mesothelin (MSLN)-targeted CAR-T cells retain essential surface molecules from their parent cells, including CARs, CD3, CD8, and TCRs. Leveraging these biological features, MSLN-targeted CAR-T cells demonstrated the ability to effectively kill triple-negative breast cancer cells in both i*n vitro* and *in vivo* studies [176]. This evidence highlights the significant antitumor potential of CAR-T cell-derived exosomes, particularly against solid tumors. In addition to their well-known function of producing specific immunoglobulins, B cells regulate immune responses through antigen presentation, costimulatory signalling, and cytokine secretion. Furthermore, B-cell-derived exosomes express essential costimulatory molecules such as CD86, CD81, and CD19, all of which play a vital role in facilitating immune activation. Additionally, natural killer (NK) cells are a critical component of the innate immune system, exhibiting functional activity against metastatic and haematological malignancies, making them a promising tool for cancer treatment. Beyond the direct use of NK cells, recent studies have highlighted the therapeutic potential of NK-derived exosomes, which demonstrate significant antitumor efficacy [177]. These exosomes are small extracellular vesicles that retain many of the functional properties of their parent NK cells. It can induce various cell death pathways in cancer cells, including endoplasmic reticulum stress-induced apoptosis, as well as both caspase-dependent and caspase-independent apoptosis. These mechanisms enable NK-derived exosomes to target and kill tumor cells selectively. Furthermore, due to the presence of cytotoxic proteins, such as interferon-gamma (IFN-γ), NK-derived exosomes can rapidly bind to recipient cancer cells, effectively inducing cytotoxicity while minimizing damage to normal tissues. Compared to NK cells, NK-derived exosomes offer several advantages. They exhibit greater stability, reduced immunogenicity, and enhanced potential for modification, making them highly versatile for therapeutic applications. The NK-derived exosomes show great potential in modulating antitumor immunity for aggressive malignancies such as melanoma, colon carcinoma, and gastric cancer [178]. Biomaterial-based immune cells interact with acute and chronic inflammatory processes (Fig. 17b). The dendritic cells (DCs), widely regarded as the most effective antigen-presenting cells (APCs), play a pivotal role in orchestrating both innate and adaptive immunity. Exosomes derived from DCs contain key immunological components, including functional antigenic complexes and T-cell costimulatory molecules, enabling them to drive specific cytotoxic T lymphocyte (CTL) responses that inhibit tumor progression. The macrophages also play a crucial role in the tumor microenvironment by activating cancer-associated fibroblasts, promoting the secretion of pro-angiogenic factors, and facilitating the transfer of signalling molecules, collectively contributing to immunosuppression. These cells exhibit functional activity of polarising into M1 or M2 subtypes [179]. The M1-polarized macrophages are characterised by the secretion of pro-inflammatory and immunostimulatory cytokines such as interleukin-12 (IL-12), interferon-gamma (IFN-γ), and tumor necrosis factor-alpha (TNF-α). These cytokines enhance immune activation and contribute to the suppression of tumors. In contrast, M2-polarized macrophages are driven by immunosuppressive cytokines, including interleukin-4 (IL-4), interleukin-10 (IL-10), interleukin-13 (IL-13), and transforming growth factor-beta (TGF-β).

**Fig. 18:**
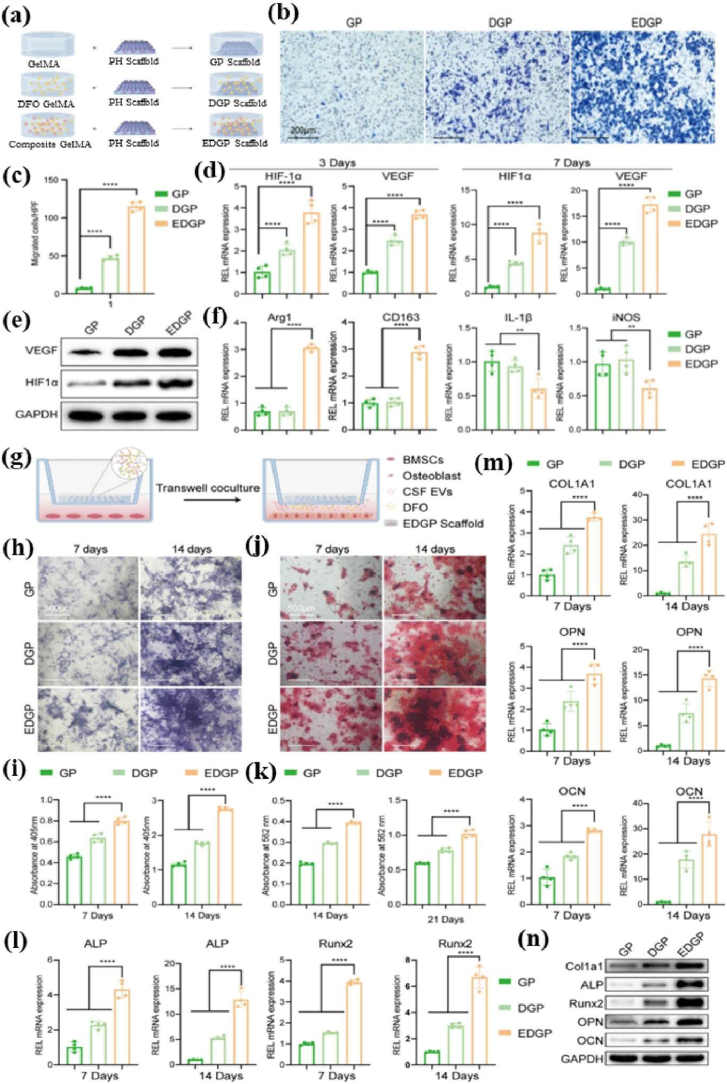
*In vitro* angiogenic evaluation of HUVECs cultured on various scaffolds: (a) schematic diagram of the synthesis of GP (GelMA hydrogel), DGP (DFO@PLGA-GelMA), and EDGP scaffolds (DFO@PLGA/EVs-GelMA). (b) HUVEC cell migration was assessed using a transwell assay. Scale bar 200 μm. (c) Quantification of transmigrated cells for each scaffold group. (d) QRT-PCR was performed for molecular assessment of angiogenic activity of HIF-1α and VEGF on days 3 and 7. (e) Western blot analysis by protein level for HIF-1α and VEGF. (f) mRNA expression levels of macrophage polarization markers (Arg1, CD163, IL-1β, and iNOS) were measured by qRT-PCR. Reproduced from Ref. [310], Copyright 2025, Elsevier. I*n vitro* osteogenic evaluation of MSCs cultured on different scaffolds: (g) Schematic of the transwell co-culture system. (h–i) ALP staining and quantitative analysis of GP, DGP, and EDGP scaffolds for days 7 and 14. (j–k) ARS staining and quantitative analysis of GP, DGP, and EDGP scaffolds for days 14 and 21. Scale bar 500 μm. (l–m) Osteogenic gene expression (ALP, Runx2, Col1A1, OCN, OPN) in MSCs analysed by qRT-PCR after 7 and 14 days. (n) Protein expression of ALP, Runx2, Col1A1, OCN, and OPN osteogenic markers was analysed by Western blot. Reproduced from Ref. [310], Copyright 2025, Elsevier. All quantitative data are represented as mean ± SD (n = 4). Statistical significance is indicated as ∗∗p < 0.01, ∗∗∗p < 0.001, and ∗∗∗p < 0.0001.

The ability of biomaterials to support immune responses in multiple biological processes. The physicochemical properties of mechanical strength, porosity, and degradability play a pivotal role in determining how immune cells respond to material functionalization. In this context, an ideal biomaterial for use in bone tissue engineering should exhibit excellent biocompatibility and synergistic interactions with surrounding tissue [309]. The *in vitro* angiogenic evaluation of HUVECs cultured on different scaffolds, including GP (GelMA hydrogel), DGP (DFO@PLGA-GelMA), and EDGP (DFO@PLGA/EVs-GelMA). The schematic diagram of fabrication of the GP, DGP, and EDGP scaffolds (Fig. 18a). The cell migration capacity of HUVECs, a critical step in angiogenesis examined by transwell assay (Fig. 18b). Quantitative analysis of transmigrated cells confirmed the superior ability of EDGP scaffolds to promote endothelial cell motility compared with GP and DGP scaffolds (Fig. 18c). Molecular assessment of angiogenic activity, including HIF-1α and VEGF expression, was performed by qRT-PCR on days 3 and 7, revealing significant upregulation of angiogenesis-related genes (Fig. 18d). Western blot analysis further validated these findings at the protein level for HIF-1α and VEGF (Fig. 18e). The mRNA expression levels of macrophage polarization markers (Arg1, CD163, IL-1β, and iNOS) were measured by qRT-PCR (Fig. 18f). Collectively, these results indicate that EDGP scaffolds not only enhance endothelial migration and angiogenic gene expression but also modulate the immune microenvironment, thereby providing a dual mechanism for promoting vascular regeneration. Furthermore, the *in vitro* osteogenic evaluation of MSCs cultured on GP, DGP, and EDGP scaffolds: Schematic representation of the transwell coculture system, illustrating interactions between MSCs and scaffold microenvironments (Fig. 18g). ALP staining and quantitative analysis of GP, DGP, and EDGP scaffolds for 7 days and 14 days (Fig. 18h and i). The osteogenic differentiation of the EDGP group highest mineralisation activity compared with other groups. ARS staining and quantitative analysis of GP, DGP, and EDGP scaffolds for 14 days and 21 days (Fig. 18j and k). The osteogenic gene expression (ALP, Runx2, Col1A1, OCN, OPN) of MSCs was analysed by qRT-PCR after 7 and 14 days (Fig. 18l and m). The protein expression of ALP, Runx2, Col1A1, OCN, and OPN osteogenic markers was analysed by Western blot (Fig. 18n). Finally, these EDGP scaffold materials have significantly enhanced angiogenesis and osteogenesis behaviour, as supported by tissue repair applications [310].

#### 6.1.2 Vascular endothelial cells

Vascular endothelial cells play a crucial role in maintaining the health and functionality of blood vessels [180]. As a selective barrier, endothelial cells regulate the exchange of nutrients and oxygen between the bloodstream and surrounding tissues, ensuring homeostasis by preventing excessive leakage and inflammation. They also play a key role in regulating blood vessel contraction and relaxation by releasing vasoactive substances [181]. For instance, endothelial cells produce nitric oxide and prostacyclin to promote vasodilation, while endothelin induces vasoconstriction. These processes are vital for maintaining proper blood flow and circulation. Beyond regulating blood flow, endothelial cells play a crucial role in hemostasis. It controls blood clotting by producing factors that maintain blood fluidity and prevent unnecessary clot formation while facilitating platelet adhesion at injury sites. This function is crucial for wound healing and preventing excessive bleeding [182]. Endothelial cells also play a pivotal role in the immune response by recruiting and activating leukocytes (white blood cells) to sites of injury or infection, thereby supporting tissue repair and helping to defend the body against pathogens. Furthermore, these cells play a crucial role in angiogenesis, which involves forming new blood vessels from pre-existing ones, a process essential for growth, healing, and adapting to changing metabolic demands. Additionally, endothelial cells regulate the transfer of solutes and macromolecules across the vascular wall, influencing tissue fluid dynamics, particularly in organs like the kidneys. Their ability to respond to various stimuli while maintaining physiological balance underscores their importance in cardiovascular tissue. Moreover, endothelial cells are multifunctional contributors to the vascular system, supporting blood flow regulation, immune response, tissue repair, and angiogenesis [183].

#### 6.1.3 Stem cells

**Fig. 19:**
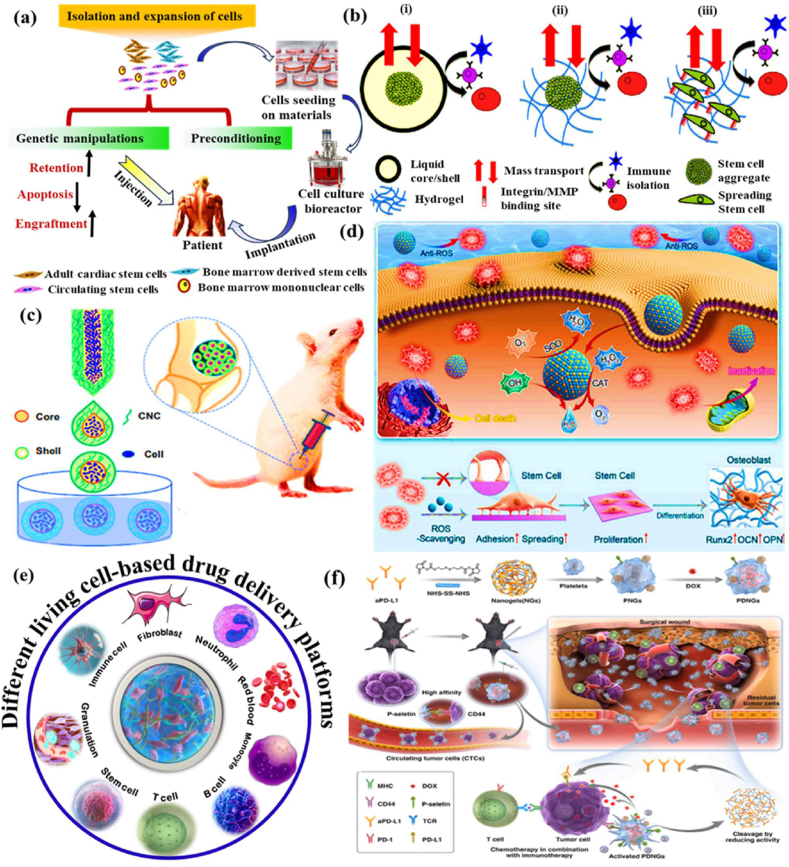
(a) Schematic diagram of stem cell isolation and expansion process for *in vitro* tissue engineering applications. (b) Schematic diagram of stem cell aggregate (i) Liquid core/solid shell encapsulation increases aggregation of facilitates the mass transit immunological isolation. (ii) Encapsulating stem cells in non-adhesive hydrogels enhances mass transit of immunological isolation. (iii) Encapsulating stem cells in sticky hydrogels containing integrin- and matrix metalloproteinase (MMP) binding sites enhances stem cell attachment while allowing for mass transit of macromolecules and immunological separation. Reproduced from Ref. [273], Copyright 2014, Bioresearch. (c) Schematic diagram of a microfluidic method for producing stem cell-loaded microcapsules for bone healing using core-shell microcapsules. Reproduced from Ref. [274], Copyright 2022, Nano-Micro Lett. (d) Schematic illustration of the extracellular and intracellular reactive oxygen species (ROS) environment, showcasing the mechanism of hMSCs functionality in cell proliferation and differentiation. Reproduced with permission from Ref. [275], Copyright 2024, Elsevier. (e) Different types of living cells used in drug delivery systems for various therapeutic applications. (f) Illustration of (anti-programmed death-ligand1) aPD-L1-crosslinked nanogels conjugated onto platelet surfaces. The polydopamine-modified PD-L1 platform demonstrated no significant impact on the viability of the decorated platelet cells. To enhance the aPD-L1 loading capacity of this platelet-based delivery system without compromising the platelets' normal function, disulfide-containing bis-N-hydroxy succinimide was employed to cross-link aPD-L1, forming uniform nanogels. These nanogels were conjugated onto platelet surfaces and cleaved in response to increased reductive activity following platelet activation. This mechanism facilitated the targeted transport of aPD-L1 to residual tumors while minimizing off-target effects during chemotherapy. Reproduced with permission from Ref. [276], Copyright 2023, Elsevier.

Stem cells play a dynamic role in regenerative medicine, particularly in the biological processes involved in tissue repair. They are classified into two generations; the first generation adult stem cells. These cells were initially used to restore hematopoietic function in patients undergoing treatments like radiotherapy or chemotherapy [184]. Among adult stem cells, mesenchymal stem cells (MSCs) are derived from bone marrow, umbilical cord, and adipose tissue. The second generation of stem cell therapies includes embryonic stem cells and induced pluripotent stem cells (iPSCs). These stem cells may be either allogeneic (derived from donors) or autologous (harvested from the patient's body). Allogeneic stem cells offer advantages such as high activity levels, rapid availability, and broad therapeutic potential. On the other hand, autologous stem cells, being derived from the patient's own body, minimize the risk of immune rejection and adverse reactions. Significantly, stem cell-based therapies have demonstrated substantial benefits over traditional interventions for various conditions, including anemia, acute cardiac disease, and Crohn's disease. Their function encompasses pathological targeting, tissue repair, anti-inflammatory responses, and immune regulation [186,187]. Additionally, the physical factors of mechanical forces and tensile strain significantly impact the functional activity of stem cells. For example, mechanical forces can promote osteogenic differentiation in mesenchymal stem cells while inhibiting adipocyte formation. Stem cell isolation and expansion process for *in vitro* tissue engineering applications (Fig. 19a). Schematic diagram of stem cell aggregate (i) Liquid core/solid shell encapsulation increases aggregation and facilitates the mass transit immunological isolation. (ii) Encapsulating stem cells in non-adhesive hydrogels enhances mass transit of immunological isolation. (iii) Encapsulating stem cells in hydrogels containing integrin and matrix metalloproteinase (MMP) binding sites enhances stem cell function while promoting mass transit of macromolecules and immunological separation (Fig. 19b). Represents the microfluidic method for producing stem cell-laden microcapsules for bone healing using core-shell microcapsules (Fig. 19c). The extracellular and intracellular reactive oxygen species (ROS) environment showcases the mechanism of human MSC functionality in cell proliferation and differentiation (Fig. 19d). Also, this process enhances cell retention viability and facilitates more effective healing responses.

## 7 Cell regulation and cell function in cancer therapy

The regulation of cellular functions is crucial for maintaining a balance between normal cells and cancerous cells within the human body. In normal cells, the cell cycle is tightly regulated, ensuring controlled growth and division. However, cancer cells often carry mutations in genes that uncontrol this process, allowing them to divide without restraint. One of the hallmarks of cancer therapies aims to reactivate apoptotic pathways or block anti-apoptotic signals [190]. Significantly, oncogenes are mutated genes that drive uncontrolled cell division, while tumor suppressor genes normally regulate cell division and promote repair. Additionally, the angiogenesis process of forming new blood vessels is a critical factor in cancer progression, as tumors rely on a blood supply to sustain growth and metastasis. Anti-angiogenic therapies aim to starve tumors by preventing the formation of new vessels. Cancer cells are also more susceptible to DNA damage caused by genetic mutations or environmental stressors. Many therapies exploit this vulnerability by inducing additional DNA damage, which cancer cells struggle to repair, ultimately leading to their death. Thus, understanding how cells regulate their functions of apoptosis, necroptosis, and ferroptosis processes [191]. By activating these pathways, it can promote tumor cell death and overcome resistance to other treatments. Metabolic regulation also plays a key role in cancer progression, especially through the c-Myc (MYC) oncogene. Moreover, MYC-driven metabolic reprogramming helps cancer cells meet the increased demands of rapid proliferation, making MYC a critical therapeutic target [192]. The tumor microenvironment (TME), a dynamic ecosystem comprising cancer stem cells, immune cells, fibroblasts, extracellular matrix, blood vessels, and signalling molecules, is crucial for tumor growth and immune evasion [193]. The TME supports cancer cell survival through mechanisms such as cellular encapsulation, where tumor cells are shielded from external threats of immune surveillance and therapeutic agents. Additionally, biomaterial capsules can create dense physical barriers that block tumor penetration. In parallel, tumors recruit immunosuppressive cells, including regulatory T cells and myeloid-derived suppressor cells, which support their immune-mediated activation [194]. Additionally, targeting these cancer therapies enhances the immune cell activation and the ability to recognise and eliminate tumor cells. This approach not only offers a traditional treatment but also holds the potential for overcoming resistance mechanisms that limit the efficacy of other therapies. To conclude, immunotherapies involving genetically engineered immune cells represent promising strategies in cancer treatment. Finally, the development of refined immunotherapies is crucial for advancing cancer treatment and improving patient outcomes.

### 7.1 Tumor microenvironment induction

The tumor microenvironment (TME) is a highly intricate and dynamic system that plays a central role in cancer progression. They are characterised by unique features such as hypoxia, acidic pH, high redox potential, elevated reactive oxygen species (ROS), overexpression of specific enzymes, and increased adenosine triphosphate levels [197]. Moreover, the biomaterial capsule has enabled the creation of TME-specific techniques that offer several advantages due to their unique qualities, facilitating effective and targeted drug delivery. However, the complexity of the TME requires diverse mechanisms and approaches to achieve optimal therapeutic outcomes. Cancer cell survival in the tumor microenvironment is closely interconnected with the extracellular matrix and stromal cells. This survival is further facilitated by a complex network of cytokines, growth factors, transcription factors, free radicals, and proteins that mediate cellular communication within the TME [17,18]. Moreover, tumor tissues are inherently heterogeneous, with cancer cells embedded in a multifaceted network of immune and stromal cells, including endothelial cells, pericytes, fibroblasts, and macrophages [198]. Two key features of the TME immunosuppression and hypoxia not only serve as hallmarks of tumor progression but also act as critical barriers to the success of cancer therapies, particularly in preventing tumor metastasis [199,200]. Immunogenic cell death (ICD) is a regulated anti-tumor immune response in which dying cells not only eliminate the tumor cells but also activate adaptive immunity through the release of danger-associated molecular patterns (DAMPs). It can be mediated by several kinds of cell death, including apoptosis, necroptosis, ferroptosis, and pyroptosis, each of which contributes uniquely to immunogenic signalling [185]. In apoptosis, DAMP release is typically limited because the process is tightly regulated and often immunologically silent. It can expose the therapeutic intervention of calreticulin (CRT), as well as the release of adenosine triphosphate (ATP) and high mobility group box 1 (HMGB1), thereby acquiring immunogenic potential. Necroptosis, a programmed necrotic mechanism that causes plasma membrane rupture and the passive release of DAMPs, such as HMGB1 and nuclear proteins are carried out by receptor-interacting protein kinases (RIPK1 and RIPK3) and mixed lineage kinase domain-like protein (MLKL). This process triggers robust inflammatory signalling and promotes dendritic cell (DC) maturation, effectively linking innate and adaptive immunity. Ferroptosis, characterised by iron-dependent lipid peroxidation and oxidative membrane damage, has also been shown to induce ICD through the release of oxidised lipid metabolites and associated DAMPs, which activate pattern recognition receptors (PRRs) on immune cells, enhancing recruitment and activation of antigen-presenting cells in the tumor microenvironment (TME) [195]. Among these, pyroptosis has recently garnered significant attention due to its highly inflammatory pathways. Also, the gasdermin protein refers to the pyroptosis and involves the formation of the plasma membrane, leading to cytoplasmic swelling, cell lysis, and rapid release of DAMPs such as ATP, HMGB1, and CRT, along with pro-inflammatory cytokines including IL-1β and IL-18. The author Qiao Yu discussed self-cascaded pyroptosis-STING initiators for catalytic metalloimmunotherapy, focusing on delivering pyroptosis-inducing agents to promote inflammatory pathways [203]. Moreover, DAMPs play a role in regulating cell death and immune activation, and their presence determines the immunogenic potential of ICD-targeted therapies. The DAMPs can be divided into two categories, including constitutive DAMPs (cDAMPs) and inducible DAMPs (iDAMPs). The cDAMPs are functionally characterised by CRT, HMGB1, ATP, and heat shock proteins (HSPs). Together, these molecules are highly supportive of producing an antitumor immune response. In contrast, iDAMPs such as tumor necrosis factor-α (TNF-α), interleukin-1 (IL-1) family cytokines, and interferons (IFNs) are actively produced in response to cell death signalling.

Organ-on-a-chip systems have recently emerged as an innovative platform for cancer research. These methods provide biomimetic microenvironments that enable the dynamic monitoring of encapsulated cell behaviour under physiologically appropriate conditions, capturing not only the primary tumor microenvironment but also metastatic niches interconnected through microfluidic networks. The incorporation of biomaterial-based cell capsules into microfluidic devices has enabled the creation of sophisticated platforms for addressing cancer treatment. The organ-on-a-chip models enable the investigation of treatment responses at both the organ and tissue levels, providing useful insights for drug screening while also deconstructing TME physiological cellular functions [13]. A significant feature of these systems is their ability to replicate the structural and functional complexity of live tissues, including inter-organ connections, transport dynamics, and tissue responses to external stimuli. However, the cancer study organ-on-a-chip platforms have demonstrated broad applications in drug screening, pharmacokinetic and pharmacodynamic analyses, toxicology testing, and the development of artificial biomimetic microenvironments [15]. Furthermore, they provide an engineering framework for integrating cells, hydrogels, and topographical cues to generate small-scale, yet functionally relevant, tissue constructs with well-defined architectures. Importantly, the convergence of organ-on-a-chip models with microfluidic encapsulation technologies introduces a new dimension to biomedical research. In particular, droplet microfluidics-based cell encapsulation enables the systematic evaluation of capsule design parameters, such as porosity, mechanical strength, and immunoisolation, within biomimetic microenvironments. Additionally, this integration enables real-time monitoring of cell encapsulation biomaterial capsules, resulting in more advanced therapeutic performance for all biomedical applications.

### 7.2 Cell delivery

Cell delivery systems control the natural capabilities of living cells to transport therapeutic agents. Various approaches are investigated for cell delivery, including injectable systems, scaffold-based delivery, and the implantation of encapsulated cells. Living cells such as red blood cells, platelets, stem cells, and immune cells have inherent biological functions, which make them well-suited for targeted drug delivery [201]. These systems overcome the barriers of traditional therapies by improving biocompatibility, extending circulation times, and navigating complex biological environments. Different types of living cells are utilized in drug delivery systems for various therapeutic applications (Fig. 19e). The PD-L1-crosslinked nanogels are conjugated to platelet surfaces. The polydopamine-modified PD-L1 (anti-programmed death-ligand1) platform demonstrated no significant impact on the viability of the decorated platelet cells. To enhance the aPD-L1 loading capacity of this platelet-based delivery system without compromising the platelet's normal function, disulfide-containing bis-N-hydroxy succinimide was used as a cross-linker to form uniform nanogels. These nanogels ensure the controlled release of the therapeutic agent, allowing it to directly interact with the tumor site. This mechanism is facilitated by targeting aPD-L1 to residual tumors while minimizing off-target effects (Fig. 19f). For example, these cells possess regenerative and natural stealth capabilities, as the CD47 receptor is expressed on their surfaces, allowing them to circulate in the bloodstream for extended periods [202]. Besides, this approach to the biological functions of biomaterial capsules using cell delivery systems is more efficient for targeted cancer therapy.

### 7.3 Red blood cells

Red blood cells (RBCs) are increasingly being investigated as natural carriers for therapeutic encapsulation due to their unique biological properties. Since they are the most common cells in the human body, their high biocompatibility, extended circulation time, and low immunogenicity make them perfect for drug and cell delivery systems [205]. Furthermore, red blood cells (RBCs) possess complement receptors on their surface, enabling them to bind to antigen-antibody complexes [206]. In RBC-based encapsulation, therapeutic agents such as enzymes, small molecules, or biomaterial hydrogels are loaded into the cells while maintaining their structural integrity. Moreover, RBC membranes can be engineered with specific ligands or receptors to direct them toward diseased tissues, such as tumors or sites of inflammation. In cancer therapy, RBC encapsulation approaches aim to transport chemotherapeutics or immunomodulators directly to the tumor microenvironment, thereby minimizing systemic side effects [207]. For instance, insulin modified with glucose analogues can be anchored to RBC membranes. The author Wang et al. [208] developed a glucose-responsive intelligent insulin delivery system using modified RBCs, where elevated glucose levels competitively displace glucose analogues on the RBC surface, triggering the controlled release of insulin and thereby helping regulate blood sugar. This strategy enables sustained release, reduced toxicity, and improved targeting compared with conventional delivery methods. Overall, RBC-based cell encapsulation represents a promising biomimetic platform that exploits the body's natural cellular machinery to achieve safe, efficient, and targeted therapeutic delivery.

### 7.4 Platelet cells

Platelet cells are non-nucleated cells derived from megakaryocytes, pivotal in hemostasis and maintaining blood vessel stability. During this process, platelets undergo significant morphological changes, absorb plasma proteins onto their surface, and facilitate the coagulation cascade, which is essential for clot formation [209]. Beyond these traditional roles, platelets have gained attention in cancer therapy due to their unique biological properties, particularly their natural ability to target immune-modulating functions. Platelets are naturally adept at homing to sites of vascular damage, surgical wounds, and circulating tumor cells (CTCs). Their surface receptors mediate adhesion to damaged vasculature and tumor cells, making them an ideal platform for targeted drug delivery. By harnessing this innate targeting ability, platelets can deliver therapeutic agents directly to tumor sites, significantly improving treatment specificity and reducing off-target effects [211]. In addition to their targeting capabilities, platelets secrete chemokines that activate T cells and other immune cells. This immune-modulating functionality positions platelets as potential facilitators of immune-based therapies. Their circulation lifespan of 8–9 days offers a pharmacokinetic advantage, providing extended delivery by maintaining biocompatibility [212]. Additionally, the platelet cell's ability to carry and release therapeutic agents in response to specific tumor microenvironment cues makes it a promising biomimetic platform. This approach has several advantages, including increasing the stability and circulation time of therapeutic agents, improving the precision of drug delivery by adhering to tumor cells and damaged tissues, reducing systemic side effects, and boosting the immune system's ability to recognise and kill tumor cells [213]. While surgery can efficiently remove the majority of a solid tumor, residual tumor cells (CTCs) that enter the bloodstream frequently cause disease progression. Furthermore, platelet cells are no longer used to mediate hemostasis because their multifunctional features have enormous potential in cancer treatment.

### 7.5 Neutrophil cells

Neutrophil cells are one of the most common forms of white blood cells in the human body. These polymorphonuclear cells play a crucial role in infection defence and innate immune response, rapidly moving to inflammation or damage to destroy pathogens via mechanisms including phagocytosis, degranulation, and the release of neutrophil extracellular traps [214]. Recently, the unique properties of neutrophils have garnered attention for their potential use in cancer therapy, particularly as drug delivery systems. Neutrophils naturally migrate toward tumor microenvironments, which are often characterised by chronic inflammation. This inherent ability, combined with their capacity to navigate complex physiological barriers such as dense extracellular matrices and immune-suppressive environments, makes them promising for targeted drug delivery. Then, neutrophils act as transport therapeutic agents, including chemotherapeutics [215] and immunomodulators, directly to tumor sites with high specificity. These properties are particularly valuable in overcoming common challenges in cancer therapy, such as drug resistance, poor penetration of therapeutic agents into tumors, and immune evasion by cancer cells. Neutrophil-mediated delivery systems have demonstrated enhanced efficacy in glioma treatment by facilitating the targeted entry of chemotherapeutic agents, inducing cytotoxicity in tumor cells, and reducing tumor size [216,217]. Moreover, neutrophil-based delivery systems offer several advantages over traditional ligand-receptor-mediated targeting methods. Additionally, surgical therapies often cause inflammation, which creates an ideal environment for neutrophils to act as carriers for delivering medications to post-operative regions, thereby enhancing recovery and eliminating tumor cells.

## 8 Flexible methods of cancer treatment

**Fig. 20:**
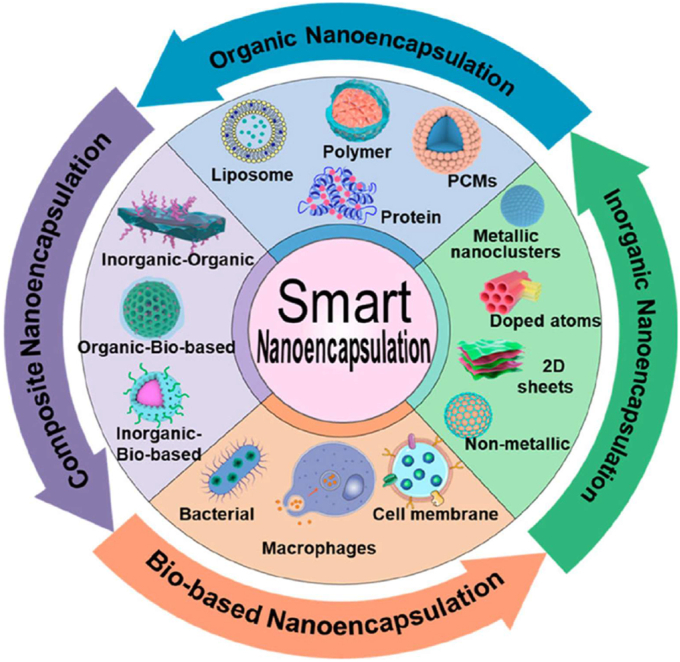
Schematic diagram of several compositions of smart nanoencapsulation strategies for cancer therapy. Reproduced from Ref. [277] Copyright 2025, Frontiers in Bioengineering and Biotechnology.

**Fig. 21:**
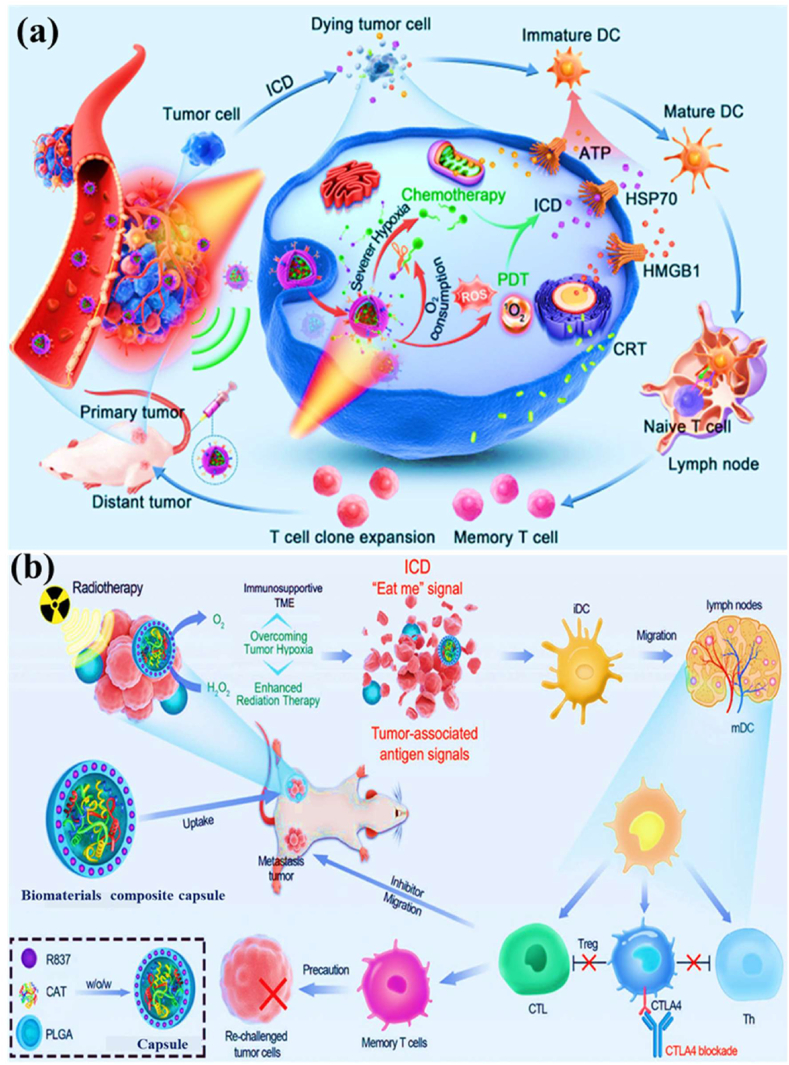
(a) Chemotherapy treatment using biomaterials to destroy tumor cells. Reproduced from Ref. [278], Copyright 2023, Nature Communications. (b) Radiotherapy treatment using biomaterial capsule to destroy tumor cells. Reproduced with permission from Ref. [279], Copyright 2019, WILEY-VCH.

Cancer treatment has undergone significant evolution in various approaches, incorporating advanced technologies to enhance the accuracy, efficacy, and adaptability of treatments targeting different types of tumor cells. The cancer cells are abnormal and no longer respond to the signals regulating normal cell function. Over time, they develop resistance to the healthy tissues, leading to rapid and uncontrolled division. As they proliferate, cancer cells outnumber the surrounding healthy cells [21]. Despite their genetic abnormalities, which make them prime targets for apoptosis (programmed cell death), cancer cells have evolved mechanisms to evade destruction. The transformation of normal cells into malignant ones occurs through a series of genetic mutations, progressing through distinct stages, including normal duct tissue, intraductal hyperplasia, intraductal atypical hyperplasia, intraductal carcinoma, and invasive carcinoma [28,29]. In the early stages, tumors do not pose a significant threat, but as they grow in size and mass, they become more dangerous, eventually damaging surrounding healthy tissues. Schematic diagram of several compositions of smart nanoencapsulation strategies for cancer therapy (Fig. 20). There are various cancer treatments available in cancer treatment, including chemotherapy, immunotherapy, radiotherapy, and photodynamic therapy. Moreover, the biomaterials have become increasingly important in cancer treatment due to their unique properties. Recently, biomaterials in drug-delivery systems have gained significant attention for their role in improving therapeutic outcomes. These materials improve pharmacokinetics, minimize systemic toxicity, and facilitate targeted delivery to tumor tissues, all of which are essential for effective cancer treatment. A biomaterial capsule was used to deliver chemotherapeutic agents and enhance radiotherapy treatment to destroy the tumor cells (Fig. 21a and b). Furthermore, combined polymer nanocarriers that are responsive to the unique tumor microenvironment (TME) have been developed for the targeted delivery of chemotherapeutics, proteins, and photosensitizers. Encapsulation technology, a cornerstone of these systems, involves packaging drugs in carriers such as nanoparticles, liposomes, micelles, hydrogels, or polymer-based capsules. This approach addresses the limitations of traditional chemotherapy, including systemic toxicity, off-target effects, and poor bioavailability. Stimuli-responsive systems release drugs selectively in response to TME-specific triggers such as acidity, enzymes, or reactive oxygen species [62]. These systems employ both passive and active targeting mechanisms. The passive targeting takes advantage of the EPR effect, enabling nanosized carriers to accumulate in tumor tissues. Active targeting, in contrast, modifies the capsule surface with ligands, antibodies, or peptides that specifically bind to cancer cells. Additionally, encapsulation enables combination chemotherapy, where multiple drugs are delivered simultaneously, thereby overcoming resistance and targeting cancer through multiple pathways. Radiation therapy, a widely used cancer treatment, utilises ionising radiation to damage cancer cell DNA and inhibit replication. However, its limitations, such as collateral damage to healthy tissues and low selectivity, highlight the need for innovative delivery methods. Encapsulating radionuclides or radiosensitizers in biocompatible carriers can enhance precision, safety, and effectiveness, significantly reduce systemic toxicity and improve targeted radiation therapy. Immunotherapy, which harnesses the immune system to identify and destroy cancer cells, has been revolutionized by advancements in monoclonal antibodies and CAR-T cells [63].

The encapsulation systems protect sensitive biomolecules such as cytokines, antigens, and nucleic acids from degradation during circulation, thereby preserving their bioactivity and enhancing therapeutic outcomes. Photodynamic therapy (PDT) combines light-sensitive drugs with specific wavelengths of light to destroy cancer cells and has been integrated with immunotherapy. This dual-action approach not only kills cancer cells through localized light irradiation but also triggers robust tumor-specific immune responses by releasing tumor-associated antigens (TAAs) and damage-associated molecular patterns (DAMPs). These responses extend beyond the treated site, targeting metastatic tumors. However, the hypoxic nature of the TME limits PDT effectiveness, as it restricts oxygen availability, which is essential for its action. To address these challenges, strategies such as oxygen-generating nanocarriers or adjuvant therapies are being explored to enhance oxygen supply and sustain anticancer immunity. Therefore, these biomaterial capsules offer a traditional treatment for enhancing therapeutic efficiency and are more effective in combating cancer diseases.

### 8.1 Model of in-vitro, ex-vivo, and in-vivo construction

The in-vitro model simulates the biological processes of living organisms. These systems replicate the key aspects of laboratory conditions, providing valuable information for various research and development applications. These assays are crucial for assessing formulation barriers in the early stages of oral drug development. Although these models only partially mimic the physiological complexity of the intestine, they remain indispensable for studying the mechanisms of drug transport. In cancer studies, *in vitro* models provide controlled environments for investigating tumor behaviour, drug responses, and ROS generation mechanisms. These studies commonly employ different approaches, including two-dimensional monolayer cultures, three-dimensional spheroid models, and organ-on-chip technologies. While two-dimensional cultures are cost-effective and widely used for high-throughput drug screening and basic cellular studies, they lack the real structural and functional complexity of tumors [219]. In contrast, three-dimensional spheroid models better replicate tumor architecture, nutrient gradients, and cell-cell interactions, making them more predictive for drug efficacy analysis. Organ-on-chip technologies further enhance the relevance of *in vitro* models by incorporating patient-derived cells and microfluidic systems to more accurately mimic tumor heterogeneity, immune interactions, and therapeutic responses [218]. For intestinal transport studies, *in vitro* models rely on biomimetic membranes of cell-based systems to evaluate the transcellular and paracellular pathways of drug transport. These models offer advantages such as precise control over experimental variables, high reproducibility, and the potential for co-culturing multiple cell types. However, limitations include their simplicity compared to actual tissue environments and their limited ability to mimic interactions with other tissues or the immune system [221].

The *ex vivo* model refers to biological experiments conducted on tissues, cells, or organs extracted from a living organism and maintained in an artificial environment. These models are widely used in cancer research as they bridge the gap between *in vitro* (cell culture) and *in vivo* (animal or human) studies [222]. The *ex vivo* systems provide a more physiologically relevant clinical setting than traditional cell cultures, while avoiding many of the ethical and logistical challenges associated with *in vivo* studies. Additionally, *ex vivo* models play a critical role in advancing cancer therapy by providing a controlled yet physiologically relevant platform for tumor behaviour and testing therapeutic strategies. This model enables the maintenance of cancer tissues or cells in a microenvironment that closely mimics *in vivo* conditions, preserving key interactions between cancer cells, stromal components, immune cells, and the extracellular matrix. Furthermore, *ex vivo* models are valuable methods for evaluating the efficacy and resistance of chemotherapeutics, targeted therapies, and immunotherapies, enabling high-throughput drug screening on patient-derived tumor slices or explants. By offering insights into tumor-immune interactions, invasion, and metastasis, these models help bridge the gap between simplistic *in vitro* systems and more complex *in vivo* studies, supporting the development of personalised and effective cancer treatments while addressing the ethical and logistical challenges of animal or human trials [223]. Correspondingly, these systems maintain the structural and functional integrity of the tissue, including features such as the mucus layer and the expression of proteins involved in drug transport and metabolism. Using excised human tissues or animal models allows for evaluating drug absorption and delivery under conditions that closely mimic the *in vivo* environment. Human tissues retain the ability to maintain their structural integrity, enzyme activity, and transporter protein expression [224], providing more accurate insights into drug behaviour across different intestinal regions. Moreover, compared to *in vitro* and *ex vivo* models, they offer a cost-effective and ethically viable alternative for studying complex biological processes in a realistic yet controlled clinical setting.

The *in vivo* method examines the inside of living organisms. These studies typically use animal models, such as mice or rats. Cancer cell lines derived from humans or animals are introduced into these models to simulate cancer growth and progression under conditions that closely mimic human disease. While human studies provide the most definitive insights into therapeutic efficacy, their high cost, time demands, low throughput, and ethical considerations limit their use to later stages of drug development [225]. In contrast, *in vitro* and *ex vivo* studies are somewhat restricted, as they only partially replicate human physiology. This limitation underscores the importance of multifactorial *in vivo* studies, which enable experimentation on intact organisms, providing insights into complex physiological interactions and pharmacokinetics. Additionally, *in vivo* cancer cell capsule studies, where cancer cells are encapsulated in biocompatible scaffolds or matrices and implanted into host organisms, provide valuable information. These capsules mimic the tumor microenvironment, offering advantages such as immune isolation, structural support, and controlled tumor growth. This approach facilitates detailed investigations into tumor size, therapeutic responses, and disease progression. Moreover, the *in vivo* studies have evaluated chemotherapeutic agents, immunotherapies, drug delivery systems, tumor evolution, angiogenesis, and metastasis, as well as the development of personalised medicine using patient-derived tumor cells [226]. Besides, this research holds immense promise for advancing new therapies and improving clinical outcomes by enhancing our understanding of cancer biology and therapeutic techniques while systematically reducing tumor size. Otherwise, different types of *in vivo* cancer models offer unique advantages and limitations. First, xenograft models involve the implantation of a human cancer cell line into immunocompromised animals, which enable tumor growth and human-specific therapeutic responses but lack functional immune systems. Second, syngeneic models utilise cancer cell lines derived from the same species as the host, such as mouse cancer cells in mice, thereby allowing for interaction with the immune system. Third, orthotopic models involve implanting cancer cells into their organs of origin, such as pancreatic cancer cells in the pancreas, to replicate the tumor microenvironment and promote metastatic behaviour. Lastly, patient-derived xenograft models involve implanting tumor tissues from patients into immunocompromised mice, preserving the heterogeneity of the original tumor and providing a more accurate model of human cancer [227].

### 8.2 Challenges and solutions of cell encapsulation

Cell encapsulation represents a promising technology in regenerative medicine for transplanted cells from the host immune system. Despite its potential, several challenges remain in achieving successful clinical translation. These include ensuring long-term biocompatibility, maintaining mechanical stability, preventing immune rejection, and developing scalable and reproducible manufacturing methods. Currently, some drawbacks of immunoisolation capsule degradation or the leakage of immunogenic molecules can provoke adverse immune responses and compromise therapeutic efficacy. To address these challenges, recent research has focused on the development of advanced biomaterials with enhanced biocompatibility and the implementation of innovative fabrication techniques that can produce uniform and mechanically robust capsules [295]. The immune-inert and immunomodulatory materials of natural polymers, polysaccharides, and synthetic biomimetic polymers are being designed to minimize protein adsorption and immune cell activation, thereby enhancing the biological activity [296]. Furthermore, encapsulated cells require a continuous supply of oxygen and nutrients to maintain viability and functionality [303]. To mitigate this, strategies for embedding angiogenic growth factors, co-encapsulating endothelial cells, and utilising porous capsule structures have been developed to facilitate host vessel infiltration and enhance long-term cell survival. Mechanical integrity is another critical factor. The encapsulation systems must strike a balance between providing sufficient structural strength for implantation and enabling controlled biodegradability to support tissue integration [304]. For clinical translation, these systems must also be produced at scale with high consistency and sterility. While microfluidic platforms offer precision in capsule fabrication, they often suffer from low throughput. In contrast, 3D printing allows for customizable architectures but still faces challenges related to resolution, biocompatibility, and cell viability.

Additionally, the smart responsiveness of biomaterial-based cell encapsulation, which is more capable of sensing pathological stimulation, has emerged as a powerful approach for regulating the immune system. For instance, pH-sensitive polymeric capsules and hydrogels can encapsulate immune checkpoint inhibitors, cytokines, or siRNAs and release them preferentially in acidic medium. The enzyme-responsive biomaterials represent another important strategy, particularly in inflammatory conditions where overexpressed enzymes, such as matrix metalloproteinases, phospholipases, or reactive oxygen species, are involved as a pathological pathway. In cancer immunotherapy, an enzyme-degradable capsule has been employed to release tumor antigens or immune agonists selectively within protease-rich TMEs, amplifying local immune responses. Similarly, enzyme-responsive scaffolds in tissue engineering have created dynamic immunomodulatory environments that regulate macrophage polarization and support tissue regeneration. Beyond pH and enzyme sensitivity, other stimuli-responsive systems have also been developed, such as redox-responsive carriers that leverage elevated glutathione levels in tumor or inflamed tissues to release therapeutic cargo. At the same time, thermo-responsive platforms can be combined with localized hyperthermia to trigger the release of immune checkpoint inhibitors. Inclusive, these smart responsive biomaterials address one of the central challenges of immunotherapy by balancing immune activation with localized immune modulation to support tumor destruction.

## 9 Cell encapsulation in regenerative medicine

**Fig. 22:**
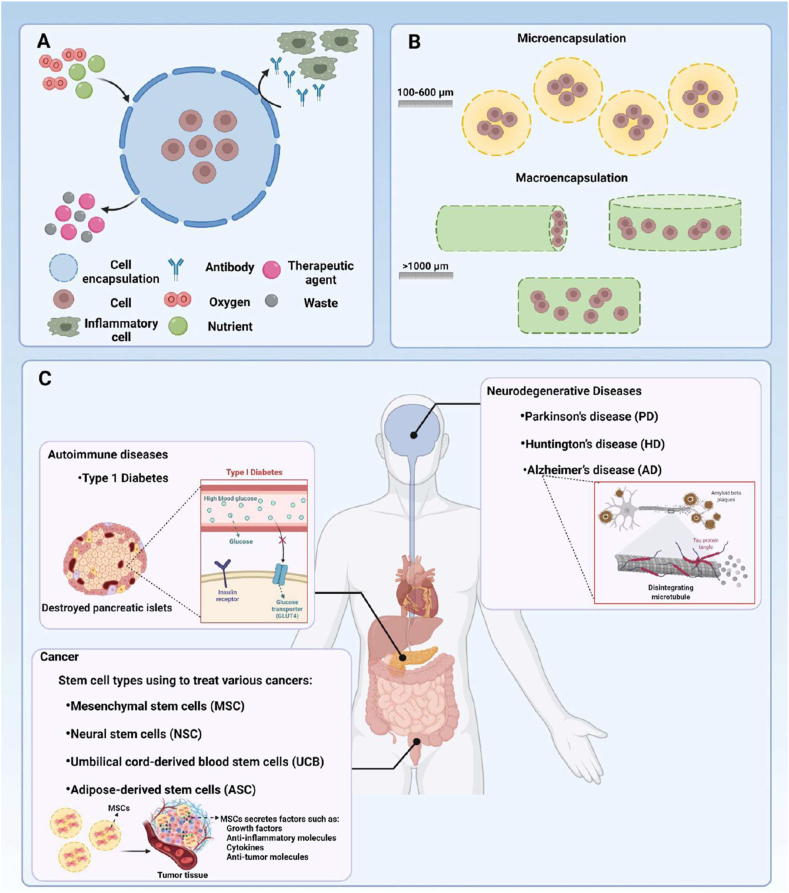
(a) Semi-permeable membrane used for micro and macrocapsules for cell transplantation. (b Schematic diagram of size-dependent microencapsulation (100–600 μm) and macroencapsulation (>1000 μm). (c) Analysing autoimmune disease treatments using various cancer stem cell types with neurodegenerative diseases. Reproduced with permission from Ref. [280], Copyright 2023, ACS.

Cell capsules play a crucial role in promoting tissue repair, stimulating wound healing, and facilitating tissue regeneration. This functionality makes them promising for different applications such as cancer therapy, tissue engineering, drug delivery, and organ regeneration. The cell capsules are primarily designed to protect transplanted cells from the host immune system, allowing them to survive, proliferate, and function within the host tissue without triggering an immune response. In tissue engineering, these biomaterial capsules are crucial for regenerating or repairing damaged tissues by introducing cells that can differentiate into specific tissues. The cell capsules have effectively addressed the challenges of cell delivery and survival by providing a protective environment for the encapsulated cells. The encapsulating stem cells within the capsule maintain their pluripotency and promote differentiation into various cell types, including cartilage, bone and nerve cells. This strategy has been explored for applications in cartilage repair, bone regeneration, and nerve tissue regeneration. A wide range of applications for cell capsules includes pancreatic islet transplantation, particularly for the treatment of diabetes. Pancreatic islet transplantation involves transplanting insulin-producing cells into diabetic patients, but the survival rate of transplanted islets is often low due to immune rejection [204]. Semi-permeable membrane used for micro and macrocapsules for cell transplantation (Fig. 22a). This membrane allows bidirectional exchange of nutrients, therapeutic agents, and metabolic waste while protecting the immune cells. This immune protective strategy is also being explored for other cell-based therapies, including gene therapy and immune cell transplantation. The schematic diagram of size-dependent microencapsulation and macroencapsulation (Fig. 22b). The analysis of autoimmune disease treatments using various cancer stem cell types with neurodegenerative diseases (Fig. 22c). Also, using encapsulated stem cells to promote the regeneration of complex organs such as the liver, heart, and lungs, researchers aim to engineer functional tissues. These cell-loaded matrices can then be used to create tissue constructs for transplantation into patients with organ failure, offering new solutions for organ regeneration [228]. Additionally, cell capsules are being explored for immunomodulation through controlled, localized drug delivery. For example, encapsulated cells can deliver anti-inflammatory agents, growth factors, or gene therapies to stimulate tissue regeneration or modulate immune responses. In some cases, the encapsulated cells themselves can be genetically engineered to produce therapeutic proteins or hormones, creating a long-term, self-sustaining drug delivery system that continuously releases these molecules at the site of injury or disease. Therapeutic applications of cancer therapy, immune modulation, and regenerative medicine in different approaches (Table 5). Overall, the versatility and functionality of cell capsules make them a powerful approach in regenerative medicine, offering solutions to some of the most significant challenges in cell therapy, tissue regeneration, and organ repair.

**Table 5:** Therapeutic applications of cancer therapy, immune modulation, and regenerative medicine in different approaches.

Therapy	Specific Area	Encapsulation Benefit	Approaches	Mechanism	Advantages	Refs
Cancer Therapy	Tumor targeted drug delivery	Improved precision, reduced off-target effects and enhanced biological activity	Drug encapsulation	Delivery of immune cells and controlled release at tumor sites	Enhanced tumor targeting, reduced toxicity and minimizes systemic side effects	[193]
Immune Modulation	Autoimmune diseases, transplantation	Immune isolation, reduced graft rejection	Combination therapies	Encapsulation of multiple agents with controlled delivery of immunosuppressive factors to modulate immune responses	Synergistic effects, reduced drug resistance and reduced immunosuppression	[199]
Regenerative Medicine	Tissue regeneration including	Rapidly promote tissue repair and enhances cell survival, promotes tissue repair	Cell transplantation with targeted drug delivery	Encapsulation protects cells from immune attack while supporting tissue integration	Improved cell viability, functionality and high precision, reduced off-target effects	[228,229]
	Bone, skin, neural, liver, vascular tissue and skeletal muscle regeneration					

### 9.1 Bone tissue repair and regeneration

**Fig. 23:**
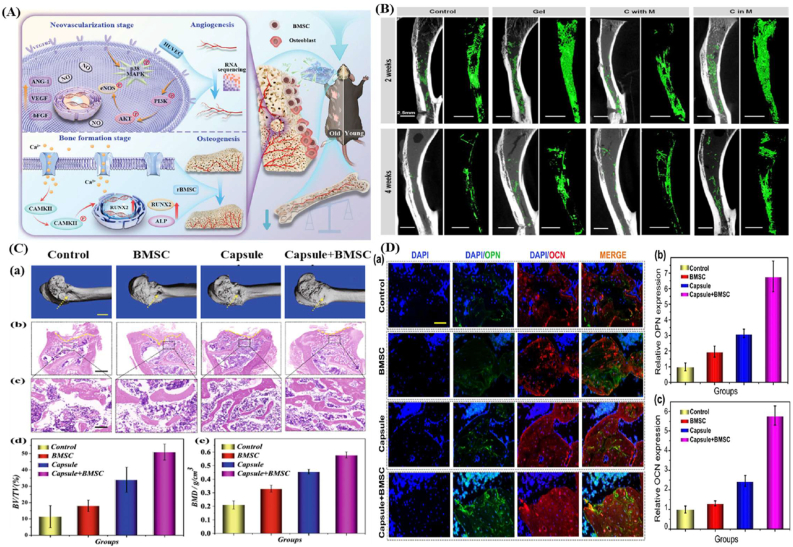
(A) Schematic diagram of the biomaterial capsule used to treat the bone regeneration of the animal model. Reproduced with permission from Ref. [281], Copyright 2025 Wiley-VCH. (B) Evaluation of bone regeneration capability using MSC-encapsulated alginate microgels *in vivo* study of 2D and 3D reconstructed micro-CT images of the tibial medullary cavity after 2 and 4 weeks post-implantation. Green regions indicate newly formed bone tissue cavity. Reproduced with permission from Ref. [245], Copyright 2020 Elsevier. (C) Micro-CT reconstruction images illustrating bone healing facilitated by the combined use of capsules and stem cells (a). Histological sections stained with H&E (b&c), and quantitative analysis of bone volume/total volume (BV/TV) and bone mineral density (BMD) across different groups: control, capsule, and capsule-BMSC (d&e). Reproduced from Ref. [274], Copyright 2022 Nano-Micro Letters. (D) Osteogenic protein expression analysis: (a) Representative immunofluorescence staining showing DAPI, DAPI/OPN, DAPI/OCN, and merged images for control, capsule, and capsule-BMSC groups. (b&c) Quantification of the relative OPN and OCN expression ratio of control, capsule, and capsule-BMSC groups. Reproduced from Ref. [274], Copyright 2022 Nano-Micro Letters.

Bone tissue engineering (BTE) is a multidisciplinary field within regenerative medicine that focuses on regenerating damaged tissue. However, bone tissue has a natural ability to regenerate effectively, but severe injuries may require extended periods. The recovery processes of bone and muscle are crucial for developing effective regenerative treatments. This field has gained considerable attention in the biomedical sector due to advancements in biomaterial capsules. These capsules provide a structured physical environment that supports tissue growth and offers mechanical support. The essential characteristics of effective biomaterial capsules include biocompatibility, biodegradability, and high mechanical strength [229]. Bone tissue engineering has recruited osteoprogenitor cells, promoting their proliferation and differentiation, facilitating extracellular matrix production, and supporting subsequent bone remodelling. The bone regeneration processes have a unique ability to self-heal through a complex process involving multiple mechanisms and cellular interactions. Schematic diagram of the biomaterial capsule used to treat bone regeneration in the animal model (Fig. 23A). The evaluation of bone regeneration capability using MSC-encapsulated alginate microgels *in vivo* study of 2D and 3D reconstructed micro-CT images of the tibial medullary cavity after 2 and 4 weeks post-implantation. Green regions indicate newly formed bone tissue cavities (Fig. 23 B). Micro-CT reconstruction images illustrate bone healing facilitated by the combined use of capsules and stem cells (a). Histological sections stained with H&E (b&c), along with quantitative analysis of bone volume/total volume (BV/TV) and bone mineral density (BMD) across control, capsule, and capsule-BMSC groups (d&e), are showed Fig. 23 C. Osteogenic protein expression analysis: (a) Representative immunofluorescence staining showing DAPI, DAPI/OPN, DAPI/OCN, and merged images for the control, capsule, and capsule-BMSC groups, (b&c) Quantification of the relative OPN and OCN expression ratios in the control, capsule, and capsule-BMSC groups (Fig. 23D). During bone remodelling, stem cell-derived osteoclasts resorb damaged bone to create space, while osteoblasts reconstruct bone tissue restoring structure [230]. Moreover, these efforts are aimed at enhancing biomaterial capsules for two primary medical applications: bone repair and joint replacement. However, the healing process can take weeks to months, depending on the harshness of the injury. The inflammatory phase of bone healing begins in the first week, characterised by the formation of a hematoma at the fracture site. During this phase, cytokines (IL-1, IL-6, TNF-α) and growth factors (VEGF, RANKL) are activated to orchestrate the inflammatory response, involving macrophages, M1/M2 cells, and fibroblasts at the injury site. Soft callus formation occurs between the second and third weeks, with angiogenesis playing a crucial role in this process. Endothelial cells, hypertrophic chondrocytes, and osteoblasts form a soft matrix stabilising the fracture. One month later, the fracture transitions from soft to hard callus formation, supported by cytokines and growth factors such as IL-6, TGF-β, and VEGF [270]. During this phase, endothelial cells, osteocytes, osteoblasts, and osteoclasts contribute to the hardening of the bone matrix. After three months, the remodelling phase begins, which is essential for the final healing and restoration of the bone's functional structure. Although capsule-based bone healing is faster, the challenges in bone grafts are commonly used to assist healing, but each type is slightly different. Xenografts and allografts are susceptible to disease transmission and complications, such as graft failure or bone absorption. The grafts often include essential elements for osteoinduction, such as bone morphogenetic proteins (BMPs) and vascular endothelial growth factors (VEGFs). TGF-β and BMPs play a crucial role in promoting osteogenesis, angiogenesis, and bone remodelling. These processes involve the coordinated activity of osteoblasts, osteoclasts, and osteocytes [231]. The biomaterial combination of cell encapsulation techniques has significantly enhanced tissue repair and bone regrowth.

### 9.2 Skin regeneration

**Fig. 24:**
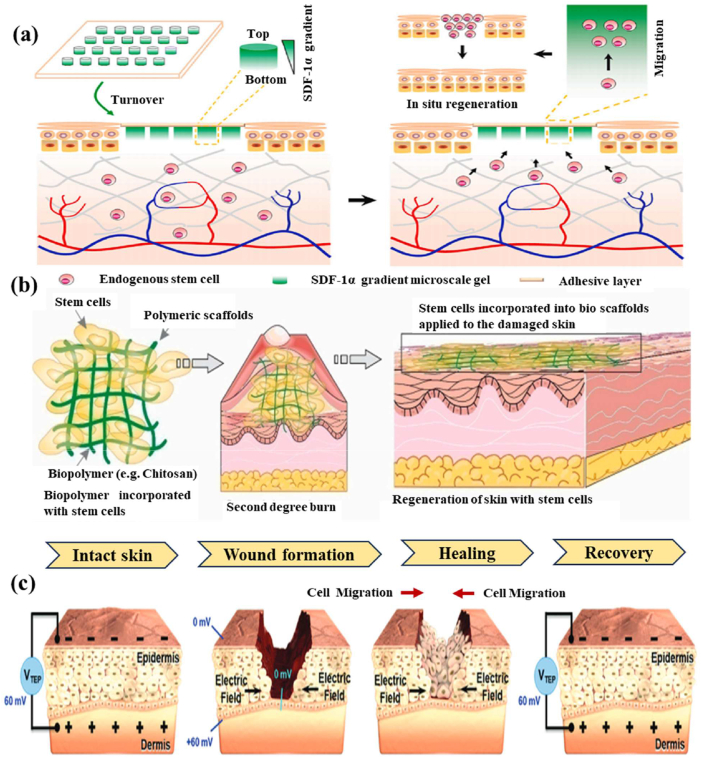
(a) A schematic illustration of a tissue engineering approach designed to accelerate wound healing using a microscale gel array patch encapsulating a defined SDF-1α gradient. Reproduced with permission from Ref. [282], Copyright 2023, Elsevier.(b) Incorporation of stem cells and polymer for enhanced skin regeneration. Reproduced with permission from Ref. [283], Copyright 2022, Elsevier. (c) Mechanism of skin regeneration facilitated by the trans-epithelial potential (TEP) during the wound healing process. Reproduced with permission from Ref. [284], Copyright 2021, Wiley-VCH.

The skin largest organ in the human body, consists of three interconnected layers: the epidermis, dermis, and hypodermis, which are sequentially arranged in an overlapping manner. Skin regeneration, which involves replacing damaged tissue with newly formed tissue, has become a cornerstone of regenerative medicine, particularly in the treatment of burns, chronic wounds, and surgical scars. A schematic illustration of a tissue engineering approach designed to accelerate wound healing using a microscale gel array patch encapsulating a defined SDF-1α gradient (Fig. 24 a). The normal wound-healing process unfolds in four stages: hemostasis, inflammation, proliferation, and remodelling [232]. Each stage of wound healing requires the coordinated action of various cell types and biologically active factors. Despite advances in traditional wound-healing methods, synthetic and natural biomaterials now play a crucial role in supporting the ECM and enhancing tissue regeneration. The treatment of small interfering RNAs (siRNAs) and microRNAs (miRNAs) regulates the complex cellular interactions during wound healing by silencing specific genes, such as MMPs, and TGFBR1, which is supported to improve the chronic wounds. Similarly, miRNAs influence critical processes like angiogenesis, fibroblast and keratinocyte proliferation, bacterial clearance, and scar management, offering significant promise for advancing wound care [233]. Moreover, skin regeneration was thought to depend solely on dermal fibroblasts and epidermal keratinocytes. It's understood as a dynamic, multifaceted process involving the cutaneous, vascular, and immune systems. Among the strategies of cell capsules has garnered attention as a cutting-edge innovation. These biomaterial-based capsules enhance healing by creating a controlled microenvironment that protects and supports cells while facilitating their delivery to damaged tissues under optimal conditions. The incorporation of stem cells and polymer for enhanced skin regeneration (Fig. 24 b). These capsules are made from biocompatible and biodegradable materials, ensuring the survival, proliferation, and functionality of the encapsulated cell. For mimicking the ECM, keratinocytes and fibroblasts of cell capsules enable the controlled release of bioactive molecules, accelerating skin repair and improving clinical outcomes in tissue engineering and wound healing, stimulating angiogenesis, enhancing collagen synthesis, and reducing inflammation [234]. Furthermore, the skin's complex, layered structure plays a critical role in the formation of distinct yet interconnected layers, including the epidermis, dermis, and hypodermis. These layers contain functional components, including appendages, capillaries, and nerve fibers, which contribute to the skin's protective capabilities [235]. Mechanism of skin regeneration facilitated by the trans-epithelial potential (TEP) during the wound healing process (Fig. 24 c). Recent advancements in tissue engineering have underscored the significance of biomaterials in skin regeneration, yielding promising clinical outcomes for patients with diabetic wounds and severe burn injuries. Finally, the biomaterial-based cell capsule therapies have been projected for treating skin defects, leveraging the versatility of polymeric materials to create scaffolds that integrate seamlessly with biological molecules and cells. These approaches hold immense promise for addressing complex skin injuries and advancing the field of regenerative medicine.

### 9.3 Neuron regeneration

**Fig. 25:**
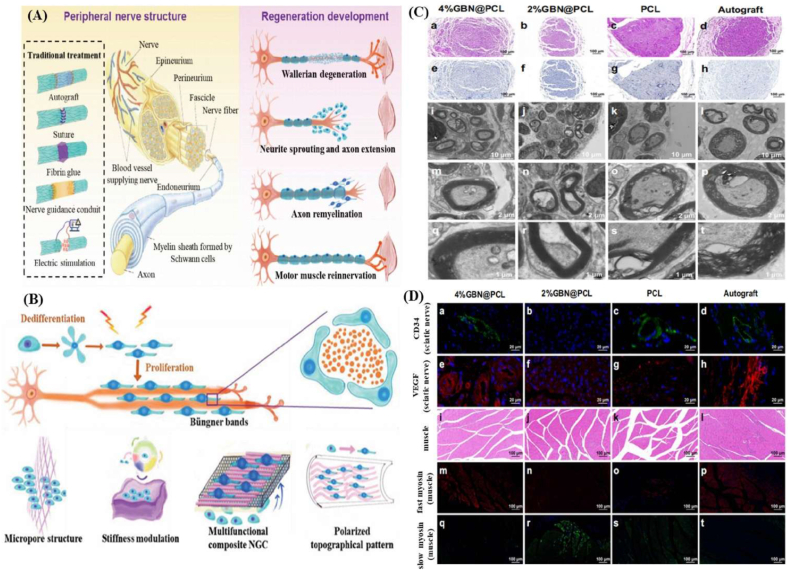
(A) Schematic diagram of the peripheral nervous system and different stages of nerve regeneration. Reproduced with permission from Ref. [286], Copyright 2024, Wiley-VCH. (B) Schwann cells dedifferentiate and proliferate to migrate to the injury site. Reproduced with permission from Ref. [286], Copyright 2024, Wiley-VCH. (C) Structural repair of peripheral nerves using graphene-based nanoscaffolds (GBN): The morphology of sciatic nerve sections was assessed using hematoxylin and eosin (HE) staining (a–d), toluidine blue (TB) staining (e–h), and transmission electron microscopy (i–t). Reproduced from Ref. [239], Copyright 2021, Nature. (D) Immunofluorescence staining of graphene-based nanoscaffolds of the regenerated nerve sections for CD34 (a–d) and VEGF (e–h), the fluorescence labelling of CD34 (green), VEGF (red), and nuclei (blue), HE staining of the gastrocnemius muscle (i–l) revealed the protective effects of GBN. Fluorescence labelling: CD34 (green), VEGF (red), and nuclei (blue). Immunostaining of the gastrocnemius muscle for fast myosin (m–p) and slow myosin (q–t). As represented, fast myosin (red), slow myosin (green), and nuclei (blue). These results indicated that relative expression levels of CD34 and VEGF in the sciatic nerves, along with fast and slow myosin in the gastrocnemius muscles, signify effective neurovascular regeneration and muscle preservation by GBN treatment. Scale bar 100 μm. Reproduced from Ref. [239], Copyright 2021, Nature.

Neuron regeneration is an emerging field utilized to treat neurodegenerative diseases, spinal cord injuries, and other neurological disorders. The nerve injuries can be classified into two categories, such as central nervous system (CNS) and peripheral nervous system (PNS). Schematic diagram of the peripheral nervous system and different stages of nerve regeneration (Fig. 25A). The PNS exhibits a remarkable regenerative capacity, primarily due to the activity of Schwann cells (SCs) dedifferentiate and proliferate to migrate to the injury site (Fig. 25B). These cells not only produce peripheral myelin but also play a critical role in nerve repair and regeneration. Upon dedifferentiation, Schwann cells align to form structures known as Bungner, which provide a supportive extracellular environment that guides and promotes directional axonal regeneration [236]. The central nervous system comprises the brain, spinal cord, ganglia, and a network of interconnected nerve cords, forming the core of the nervous system along the central axis of the body. It is essential for maintaining internal homeostasis, preserving structural and functional integrity, and enabling interaction with the external environment. Unlike the PNS, the adult CNS has a limited regenerative capacity. Axons in the CNS typically do not regenerate spontaneously, largely due to the inhibitory environment and lack of supportive cellular responses. One promising approach to promote neuron regeneration involves the use of cell encapsulation technologies. These strategies aim to enhance cell survival, proliferation, and targeted delivery to damaged neural tissues [237]. Microcapsules typically create a protective and controlled microenvironment around encapsulated cells, shielding them from immune rejection while permitting the exchange of essential nutrients, oxygen, and signalling molecules. Once implanted, the encapsulated cells can differentiate into neurons or glial cells, integrate with the host tissue, and contribute to functional repair. Structural repair of peripheral nerves using graphene-based nanoscaffolds (GBN) was evaluated by hematoxylin and eosin (HE) staining (a-d), toluidine blue (TB) staining (e-h), and transmission electron microscopy (i-t), as shown (Fig. 25C). The immunofluorescence staining of regenerated nerve sections with GBN for CD34 (a-d) and VEGF (e-h), with fluorescence labelling of CD34 (green), VEGF (red), and nuclei (blue), as well as HE staining of the gastrocnemius muscle (i-l), revealed the protective effects of GBN. Immunostaining of the gastrocnemius muscle for fast myosin (m-p) as red, slow myosin (q-t) as green, and nuclei (blue) was further confirmed muscle preservation (Fig. 25D). Collectively, these results demonstrate that the relative expression levels of CD34 and VEGF in the sciatic nerves, along with fast and slow myosin in the gastrocnemius muscles, indicate effective neurovascular regeneration and muscle preservation by GBN treatment. Moreover, the biomaterial-based capsules provide both immunoprotection and therapeutic action, facilitating the release of bioactive molecules and offering structural and biochemical cues that mimic the extracellular matrix, thereby enhancing the regenerative process [238]. In addition to fostering peripheral nerve regeneration, which is occasionally accompanied by scar tissue formation, these capsules demonstrate potential in central nervous system (CNS) repair through the release of neurotrophic growth factors, the reduction of toxic and inhibitory signals, and the support of axonal regeneration [239]. Ultimately, biomaterial-based cell encapsulation matrices demonstrate significant potential in delivering therapeutic signals and facilitating regeneration in both peripheral and central nerves.

### 9.4 Liver regeneration

Liver regeneration involves the interaction of several cell types, such as inflammatory, endothelial, hepatocyte, and hepatic stellate cells. Healthy liver cells are quiescent during mitosis, but they can quickly enter the cell cycle to restore liver function after toxic injury or resection. Both non-parenchymal and epithelial cells react in a highly coordinated manner during this regeneration process. Specifically, macrophages can support tissue regeneration, help remodel fibrosis by inhibiting the activation of hepatic stellate cells, and enhance liver regeneration. It relies on replacing the damaged tissue with the extracellular environment, which actively regulates cellular behaviour. The encapsulation of biomaterial capsules can mimic the extracellular matrix of supporting hepatocyte adhesion, metabolic activity, and specific tissue functions. The combined natural and synthetic polymer matrix exhibits highly mechanical properties, which also help to replicate the native ECM while delivering both structural and biochemical cues essential for hepatocyte cell survival [311]. By tuning physicochemical parameters such as stiffness, porosity, and degradability, these biomaterials can be optimised to support hepatocyte adhesion, metabolic functions, and functional maturation. Today, microfluidics and 3D bioprinting techniques are well established for creating cell-encapsulated systems with precisely defined, complex structures. These technologies not only maintain cell viability but also support the spatial organization of multiple liver-relevant cell types, including hepatocytes, endothelial cells, and fibroblasts, thereby fostering vascularization and seamless integration with the host tissue. Importantly, the bioactive capsule mimics physiological function by releasing growth factors and cytokines in real time, enhancing hepatocyte activity through stimulation of angiogenesis and accelerating tissue repair [312]. Beyond their structural role, biomaterial encapsulation systems also hold therapeutic potential for chronic liver failure. Their dynamic biological responsiveness under pathological conditions further strengthens their applicability. Collectively, biomaterial-based cell encapsulation offers precise control over biochemical signalling, ultimately enabling the development of implantable liver constructs capable of restoring long-term functional activity.

### 9.5 Vascular tissue regeneration

Vascular tissue regeneration is a complex structure composed of diverse cell types, all of which depend on the presence of blood vessels. The function of vascular tissue is essential for delivering nutrients, transporting oxygen, disposing of metabolic waste, and maintaining long-term activity [313]. To achieve successful vascularized tissue regeneration, a hierarchical vascular network is required that ensures continuous blood flow to various parts of the tissue. Therefore, combining biomaterial-based cell encapsulation approaches can accelerate functional recovery by modulating immune processes. One of the biggest challenges in large-scale synthetic tissues is maintaining sufficient vascularization while managing immunological responses to chronic inflammation. Despite significant advances, many vascular grafts still fail due to an inadequate number of endothelial progenitor cells (EPCs) and incomplete endothelialisation. To address these limitations, cell encapsulation techniques are increasingly being combined with bioactive compounds. Similarly, encapsulated factor-releasing cells or bioactive scaffold coatings deliver spatiotemporally controlled stimuli that mimic normal vascular remodelling. These implantations specifically promote the endothelialisation and avoid thrombosis. Besides, cell-loaded encapsulation techniques offer localized immunomodulation by secreting anti-inflammatory substances or trapping excess cytokines, thereby reducing immune suppression and supporting host tissue regeneration [314]. For example, 3D-printed vascular grafts made from elastomeric polymers, combined with pro-angiogenic cues and controlled release systems, have achieved high patency rates and robust endothelial coverage in preclinical models. Moreover, these systems enable precise modulation of immune cell activity, reducing systemic inflammation while encouraging localized regeneration. Overall, cell-encapsulated biomaterial capsules also serve as drug delivery platforms, regulating immune responses and supporting vascular tissue regeneration.

### 9.6 Skeletal muscle regeneration

In the human body, approximately 30–40 % of total mass is composed of skeletal muscle tissue. While skeletal muscle has a robust regenerative capacity following minor injuries, this capacity becomes insufficient in cases of severe trauma, volumetric muscle loss, or chronic disease, often resulting in long-term structural and functional impairments. The early phase of muscle injury is characterised by necrosis of damaged fibers, triggered by restricted blood flow, sarcolemma disruption, and calcium-dependent proteolysis. A tightly regulated immune response rapidly follows this necrotic phase. Neutrophils are the first immune cells to infiltrate the injury site, where they release proteases and reactive oxygen species to degrade cellular debris [315]. Their activity also establishes an inflammatory milieu that facilitates the recruitment of macrophages and other immune populations. The macrophages play a dual role in pro-inflammatory subsets amplify the clearance of necrotic tissue, and pro-regenerative subsets promote angiogenesis to resolve inflammation by stimulating satellite cell activation, thereby linking immune response to tissue regeneration. However, in large injuries, the regenerative potential of satellite cells is often limited by inflammatory dysregulation, fibrotic scar formation, and inadequate vascularization. To overcome these barriers, encapsulating therapeutic cells, such as satellite cells, myoblasts, or mesenchymal stem cells, within biocompatible matrices provides mechanical protection, shields cells from immune-mediated rejection, maintains cell viability, and enhances the diffusion of nutrients, oxygen, and signalling molecules. Biomaterial-based encapsulation systems are increasingly engineered to replicate the structural, biochemical, and mechanical features of native muscle. Biomaterial capsules have garnered attention as biomimetic scaffolds due to their high water content, compliance, and interconnected porosity, which support cell infiltration, vascular ingrowth, and tissue remodelling. Moreover, these systems actively modulate local inflammation, minimize fibrotic deposition, and facilitate the transmission of contractile forces across regenerating tissue by forming conformal interfaces with dynamically contracting muscle [316]. Collectively, these properties make biomaterial encapsulation platforms especially valuable for advancing skeletal muscle repair.

### 9.7 Foreign body reaction to the biomaterials

**Fig. 26:**
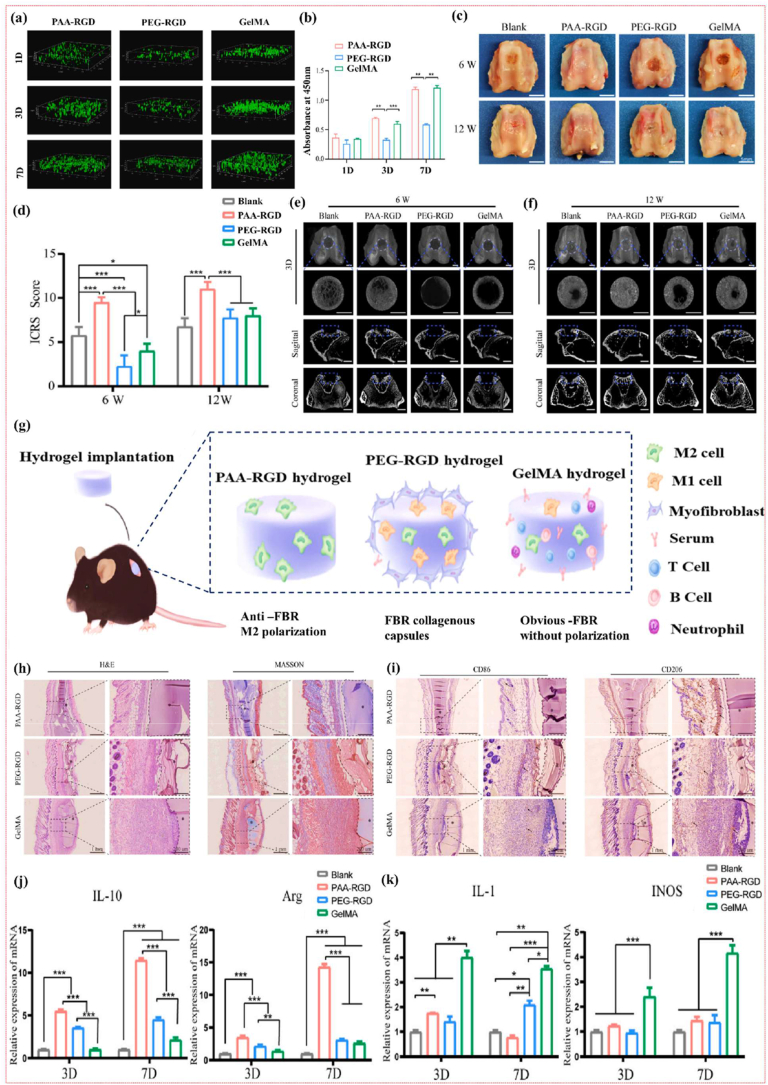
(a) Cell proliferation of 3D plot in MSCs encapsulated with PAA-RGD, PEG-RGD, and GelMA hydrogel was evaluated at 1, 3, and 7 days. Reproduced from Ref. [319], Copyright 2023, Elsevier (b) Quantitative analysis of MSCs encapsulated with PAA-RGD, PEG-RGD, and GelMA hydrogel as determined by the CCK-8 assay. Reproduced from Ref. [319], Copyright 2023, Elsevier (c) Macroscopic appearance of osteochondral tissue regeneration at weeks 6 and 12 for the treated groups: blank, PAA-RGD, PEG-RGD, and GelMA. Scale bar 5 mm. Reproduced from Ref. [319], Copyright 2023, Elsevier. (d) Bar representation graph of ICRS macroscopic assessment scores for blank, PAA-RGD, PEG-RGD, and GelMA at weeks 6 and 12. Reproduced from Ref. [319], Copyright 2023, Elsevier. (e–f) Shows the micro CT images of blank, PAA-RGD, PEG-RGD, and GelMA subchondral bone regeneration at 6 and 12 weeks. Scale bar 2.5 mm. Reproduced from Ref. [319], Copyright 2023, Elsevier. (g) Schematic diagram of a hydrogel implanted in C57/BL6 mice. (h) Histological and immunohistochemical analyses of explanted tissues after 14 days, including H&E, Masson's trichrome staining with brown arrows representing positive cells, while cell nuclei appear blue with hematoxylin. Scale bars: 1 mm and 200 μm. Reproduced from Ref. [319], Copyright 2023, Elsevier. (i) Immunohistochemical staining showed anti-inflammatory CD206^+^M2 macrophage and pro-inflammatory CD86^+^M1 macrophage responses. Reproduced from Ref. [319], Copyright 2023, Elsevier. (j–k) Quantitative analysis of macrophage polarization measured by qRT-PCR, the mRNA expression levels of M2-associated genes (IL-10, Arg-1) and M1-associated genes (IL-1β, iNOS) were measured in RAW 264.7 cells cultured with blank, PAA-RGD, PEG-RGD, and GelMA hydrogels for 3 and 7 days. Data are expressed as mean ± S.D. (n ≥ 3). Two-way ANOVA determined the statistical significance of all the quantitative images with Tukey's post hoc test. ∗P < 0.05, ∗∗P < 0.01, ∗∗∗P < 0.001. Reproduced from Ref. [319], Copyright 2023, Elsevier.

On the biological side, foreign body reaction (FBR) is one of the inflammatory responses, which encompasses different processes such as protein adsorption, acute and chronic inflammation, and collagen encapsulation [317]. Following implantation of a medical device, nonspecific protein adsorption on the biomaterial surface activates neutrophils and macrophages. These cells interact with both pro-fibrotic and anti-inflammatory mediators, marking the end-stage of the inflammatory and wound-healing response. The surface modifications of biomimetic extracellular matrix components, such as collagen, fibroin, and hyaluronic acid, could attenuate macrophage activation of immune responses towards pro-regenerative pathways. Similarly, antifouling coatings, especially poly (ethylene glycol) (PEG), are designed to reduce nonspecific protein adsorption and suppress early immune activation. Additionally, PEG susceptibility to oxidative degradation and potential immunogenicity highlight the need for more stable and less immunoreactive alternatives. However, the long-term functionality of implanted biomaterials depends on achieving a balance between mitigating harmful immune reactions, such as fibrosis and FBR, and promoting constructive immune modulation that supports integration, vascularization, and cell survival [318]. The cell proliferation of the 3D plot in MSCs encapsulated in the different hydrogels, such as Poly (amino acid) (PAA), poly (acrylic acid) functionalized with arginine-glycine-aspartic acid (PAA-RGD), polyethylene glycol-RGD (PEG-RGD), and gelatin methacryloyl (GelMA) hydrogels, was systematically evaluated at 1, 3, and 7 days (Fig. 26a). These results indicated that all the hydrogels supported high cell viability and progressive proliferation, indicating favourable cell-matrix interactions. Quantitative analysis using CCK-8 assays revealed that MSCs proliferated most effectively in PAA-RGD and GelMA hydrogels, while PEG-RGD supported relatively lower expansion (Fig. 26 b). The macroscopic appearance of osteochondral tissue regeneration at 6 and 12 weeks in the blank, PAA-RGD, PEG-RGD, and GelMA groups (Fig. 26c), with corresponding international cartilage repair society (ICRS) macroscopic assessment scores presented (Fig. 26 d). The PAA-RGD group demonstrated markedly enhanced repair, characterised by cartilage tissue formation at week 6 and complete replacement with mature cartilage tissue by week 12. In contrast, PEG-RGD and GelMA groups displayed disrupted cartilage surfaces and pronounced edema signals. Micro-CT imaging further confirmed superior subchondral bone regeneration in the PAA-RGD group compared with other groups (Fig. 26 e and f). The foreign body reaction (FBR) and macrophage polarization induced by different hydrogels were investigated *in vivo* and *in vitro*. A schematic of hydrogel implantation in C57/BL6 mice is shown in Fig. 26 g. Histological and immunohistochemical analyses after 14 days, including H&E and Masson's trichrome staining, demonstrated minimal cell infiltration and collagen deposition in the PAA-RGD group compared with PEG-RGD and GelMA (Fig. 26 h). Immunohistochemical staining further revealed anti-inflammatory CD206^+^ M2 macrophages and pro-inflammatory CD86^+^ M1 macrophages (Fig. 26 i). The qRT-PCR analysis of RAW264.7 cells cultured with the different hydrogels for 3 and 7 days showed increased expression of M2-associated genes (IL-10, Arg-1) and reduced expression of M1-associated genes (IL-1β, iNOS) in the PAA-RGD group (Fig. 26j and k). Together, these results demonstrate that PAA-RGD hydrogel not only supports robust cell proliferation and osteochondral regeneration but also modulates FBR by promoting a pro-regenerative immune response while suppressing chronic inflammation. These findings underscore the strategic potential of hydrogel biomaterials to balance cell survival, tissue integration, and immune tolerance.

## 10 Conclusion and perspective

This review discussed that cell-encapsulated biomaterial capsules are made using various methods like microfluidics, 3D printing, in situ techniques, and electrospraying self-assembly. These methods enable the creation of single-cell, multicellular, and core-shell capsules, broadening therapeutic uses. Each technique addresses specific clinical needs, whether by promoting cell longevity, enhancing cell-cell interactions, or reducing immune rejection. This adaptability makes cell encapsulation highly versatile, with applications ranging from tissue regeneration to cancer immunotherapy. Among these technologies, the microfluidics method has emerged as a powerful tool for cell encapsulation due to its biocompatibility, optical transparency, and compatibility with conventional microscopy and molecular analysis. Additionally, 3D printing has simplified the fabrication of encapsulation, facilitating the creation of physiologically relevant structures. These technologies are being increasingly adopted in biological laboratories, enabling cell-based assays suitable for biomedical applications. Moreover, cell-encapsulated biomaterial capsules are crucial in cancer research because they closely resemble the tumor microenvironment, including features like hypoxia, nutrient gradients, and cell-cell interactions. Correspondingly, in regenerative medicine, stem cells have shown great potential for tissue repair and organ regeneration due to their enhanced ability to differentiate the growth factors. Besides, these methods use capsule synthesis, which not only enables precise spatial distribution of transplanted cells but also allows for highly localized immune cell regulation.

In translational medicine, natural and synthetic polymers are essential role in supporting the high biocompatibility, cell adhesion, mechanical flexibility, and controlled degradation rates. The natural and synthetic polymer combination of the composite matrix could modulate the fibrosis and inflammation in drug delivery while regulating immune responses. The biomaterials capsule can influence fibrosis and inflammation in drug delivery, while hydrogel encapsulation prevents direct immune cell interactions. Otherwise, immunomodulatory platforms are crucial for transplantation cells, demonstrating great potential in enhancing the longevity of cellular therapies with minimal reliance on systemic immunosuppression. In addition, confirming the long-term durability of a cellular implant requires immune modulation to support the cell transplant site. Therefore, the development of biomaterial capsules is supported by regenerative medicine, and it more benefits from transdisciplinary approaches that integrate materials science, immunology, and bioengineering. Also, this empowers biomaterials to meet the specific demands of applications in both cancer therapy and tissue regeneration. In cancer therapy, encapsulated cells are being investigated for their ability to deliver therapeutic agents with greater precision, and also to improve the anti-tumor immunity. In tissue regeneration, encapsulated stem cells are supported to regenerate bone, skin, and neurons, while osteogenic cells are encapsulated in biomaterials promoting mineralisation and angiogenesis for treating fractures of osteoporosis, osteoarthritis, and explored for wound healing with neurodegenerative diseases. Furthermore, pre-vascularized capsules integrated with angiogenic factors are being developed to enhance the survival of transplanted cells. Overall, this review highlights how biomaterials combined with cell encapsulation matrices support the biological activity in cancer immunotherapy and tissue regeneration platforms, offering a broad spectrum of biomedical applications.

### 10.1 Challenges and limitations of cell encapsulation in translational medicine

Large-scale manufacturing and ensuring reproducibility remain major challenges for the clinical translation of cell encapsulation systems. Although many platforms demonstrate strong efficacy in preclinical studies, their progress is hindered by difficulties in standardizing fabrication across diverse cell types, encapsulation matrices, and multiple device formats. The 3D printing and microfluidics techniques offer high precision at small scales but face significant limitations in throughput, cost-effectiveness, and batch-to-batch differentiation for clinical production. The reproducibility of encapsulation systems is further challenged by variability in cell sources, inconsistencies in material quality, and fluctuations in encapsulation parameters. Beyond these technical hurdles, one of the most persistent challenges lies in integrating biomaterials with living cells in a way that achieves truly selective permeability. Such selectivity is indispensable for enabling the efficient exchange of nutrients, oxygen, and therapeutic molecules while simultaneously excluding immune components. A major barrier to clinical translation is the need to maintain long-term biocompatibility while minimizing adverse immune responses. Although encapsulation materials are designed to shield therapeutic cells from direct immune recognition, implantation frequently provokes host reactions. These responses often present as fibrotic encapsulation, chronic inflammation, or a progressive decline in permeability, collectively undermining cell viability and diminishing therapeutic efficacy. However, immune activation is influenced not only by the bulk composition of the material but also by more subtle factors, including porosity, degradation rate, and the mechanical properties of the encapsulation matrix. Addressing these challenges will require integrated approaches that combine biomaterial innovation with insights from immunology, precision medicine, and host tissue interaction.

### 10.2 Clinical implementation and future direction of cell encapsulation

The clinical applicability of cell encapsulation technologies has expanded in multiple fields. However, their broader adoption requires overcoming key challenges, including immune rejection, fibrotic encapsulation, and limitations in mass transport. Biomaterial-based cell encapsulation holds transformative potential by redefining how therapeutic cells interact with the host environment. Unlike conventional drug delivery systems or systemic cell transplantation, encapsulation establishes a protective yet functional niche that shields cells from immune-mediated destruction while maintaining therapeutic efficacy. The future direction of the field lies in personalised encapsulation platforms, where biomaterials are tailored to patient-specific immune profiles, metabolic needs, and disease microenvironments. For instance, capsule matrices can be customised with patient-specific extracellular matrix components or bioactive peptides to enhance cell survival and function. Emerging smart biomaterials capable of responding to inflammatory signals or mechanical stress add an extra layer of adaptability, ensuring long-term engraftment and dynamic interaction with host tissue. The personalised biomaterials are already demonstrating promise in improving treatment platforms for diabetes, neurodegeneration, oncology, and regenerative medicine by converting encapsulating structures into live, adaptable implants that are both patient-specific and disease-specific. A prominent trend in the field is the development of smart, stimuli-responsive biomaterials that dynamically respond to environmental cues, such as pH, oxygen tension, or inflammatory signals, enabling the real-time management of cell survival and function. Furthermore, encapsulated systems are likely to include synthetic biology circuits, allowing designed cells within protected niches to detect the infection biomarkers. From a translational standpoint, cell encapsulation serves not only as a passive barrier but also as an active participant in tissue integration, immunomodulation, and regeneration. The impact is represented by applications ranging from restoring pancreatic islet function in diabetes to creating neural repair niches in neurodegenerative disorders and enhancing the persistence of adoptive immune cells in cancer therapy. By integrating synthetic biology, advanced materials engineering, and precision medicine, encapsulation technologies are poised to evolve into adaptive, patient-specific living implants. These innovations significantly expand the therapeutic landscape for complex chronic diseases. Collectively, emerging trends of cell encapsulation are not only a protective strategy but also a next-generation therapeutic platform capable of delivering adaptive, durable, and personalised treatments in chronic diseases, regenerative medicine, and cancer immunotherapy.

## 11 Ethical approval and informed consent statement

This is no ethics approval and consent to participant involved in this article.

## CRediT authorship contribution statement

**Mayakrishnan Arumugam:** Writing – review & editing, Writing – original draft, Data curation, Conceptualization. **Yunyang Zhang:** Validation, Software, Formal analysis. **Ying Huang:** Validation, Resources, Formal analysis. **Ramesh Kannan Perumal:** Visualization, Validation, Formal analysis. **Ting Zhang:** Validation, Resources, Formal analysis. **Xiangdong Kong:** Visualization, Project administration, Funding acquisition. **Ruibo Zhao:** Writing – review & editing, Supervision, Project administration, Investigation.

## Declaration of competing interest

The authors declare that they have no known competing financial interests or personal relationships that could have appeared to influence the work reported in this paper.

## Abbreviations

APCs-antigen-presenting cells; AI-Artificial intelligence; ATP-adenosine triphosphate; CTCs-circulating tumor cells; CAR-T- chimeric antigen receptor T; CNS-central nervous system; CPS-capsular polysaccharides; CSC-cancer stem cell; DCs-dendritic cells; DAMPs-damage-associated molecular patterns; ECs-endothelial cells; ELP-elastin-like polypeptide; ECM-extracellular matrix; EPCs-endothelial progenitor cells; EBP-Elastin-binding protein; EPS-exopolysaccharides; FBR-foreign body reaction; FBs-fibroblasts; FDM-fused deposition modelling; FDA-Food and Drug Administration; hPSCs-human pluripotent stem cells; H&E-Hematoxylin and Eosin; iPSCs-induced pluripotent stem cells; ICIs-immune checkpoint inhibitors; ICD-Immunogenic cell death; LBL-layer-by-layer; ICRS-International Cartilage Repair Society; MSCs-mesenchymal stem cells; ML-Machine learning; MMP-matrix metalloproteinase; NK-natural killer; NF-Nanofibers; PDT-Photodynamic therapy; PEG-Polyethylene glycol; PLA-Polylactic acid; PVA-Polyvinyl alcohol; PCL- Polycaprolactone; PDMS-polydimethylsiloxane; PRRs-pattern recognition receptors; ROS-reactive oxygen species; RCD-regulated cell death; SASP-senescence-associated secretory phenotype; SMCs-smooth muscle cells; TME-tumor microenvironment; TAAs-tumor-associated antigens. TGF-β-transforming growth factor-β.
